# Broadening the catalytic region from the cavity to windows by M_6_L_12_ nanospheres in cyclizations†

**DOI:** 10.1039/d3sc02998k

**Published:** 2023-09-26

**Authors:** Meiling Xu, Bin Sun, David A. Poole, Eduard O. Bobylev, Xu Jing, Jinguo Wu, Cheng He, Chunying Duan, Joost N. H. Reek

**Affiliations:** a State Key Laboratory of Fine Chemicals, Dalian University of Technology Dalian 116024 P. R. China xjing@dlut.edu.cn cyduan@dlut.edu.cn; b Homogeneous, Supramolecular and Bio-Inspired Catalysis, Van't Hoff Institute for Molecular Sciences, University of Amsterdam Science Park 904 Amsterdam 1098 XH The Netherlands j.n.h.reek@uva.nl

## Abstract

Supramolecular cages have received tremendous attention as they can contain catalysts that exhibit confinement effects in the cavity, leading to excellent performances. Herein, we report an example wherein the catalytic region is extended from the cage cavity to the windows, and investigate its confinement effect by utilizing the Pd_6_L^Au^_12_ cage that contains rigidly fixed and isolated gold complexes at the windows. Pd_6_L^Au^_12_ exhibit three features of particular interest while assessing their properties in gold-catalyzed cyclization reactions. First, the catalysts experience a cage effect as they display higher reactivity and selectivity compared to the monomeric analogue, as a result of substrate pre-organization at the windows. Second, the metal complexes are physically separated by the cage structure, preventing the formation of less active dinuclear gold complexes making it more stable under hydrous conditions. Third, the cage windows present the characteristics of enzymatic catalysis *via* Michaelis–Menten-type mechanism analysis. This contribution presents an alternative way to engineer supramolecular catalysts through extending the catalytic region.

## Abstract 2

M_6_L_12_ nanospheres, with isolated gold sites fixed on the windows, were utilized as an example to prove that the cage windows present a confinement effect for substrate pre-organization and transition state stabilization leading to high rates.

## Graphical abstract

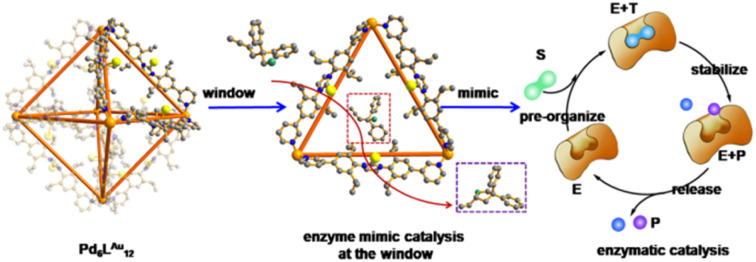

## Introduction

Over the past few decades, supramolecular chemistry has experienced tremendous developments, particularly for potential applications in drug transport,^1^ recognition and storage,^2^ sensing,^3^ and catalysis.^4^ Inspired by the ingenious protein structures of enzymes in nature providing well-defined pockets around the active sites, supramolecular coordination cages have been explored to emulate the specific microenvironment of enzymes. Several examples have demonstrated that synthetic mimics can provide an ideal reaction container to regulate the reactivity.^5^ By applying supramolecular strategies, a catalytic system could be formed in which single or multiple active sites reside in a confined micro-environment. In such a micro-environment, the orientation and rotation of substrates and catalysts can be restricted, explaining some of the unusual selectivity displayed by these encaged catalysts. In addition, the restriction of substrate and intermediate conformations within the cavity can be entropically favorable, leading to improved activity.^6^ Based on these successes, supramolecular strategies in transition metal catalysis developed as a fruitful tool for catalysis tuning, as specific activity and unprecedented selectivity can be achieved.

A powerful strategy to produce pyridine-coordinated palladium M_*n*_L_2*n*_ (*n* = 6, 12, 24) cages was pioneered by Fujita.^7^ The combination of the ditopic bispyridine building blocks and palladium complexes typically afforded self-assembled structures such as M_6_L_12_, M_12_L_24_ and M_24_L_48_,^8,9^ depending on the bend angle of the bispyridine building blocks. The M_12_L_24_ nanospheres are amongst the most widely studied, as they can be functionalized with facile endohedral or exohedral binding groups, thereby generating a confined microenvironment suited for potential applications.^10^ We previously reported the utilization of Fujita-type M_12_L_24_ nanospheres that were decorated internally with 24 gold complexes to provide systems with extremely high local concentration of gold species.^11^ It led to d_10_–d_10_ aurophilic interaction and, as a result, enhanced the reactivity in cyclization reactions.^11^ More recently, we reported that an M_12_L_24_ nanosphere was able to bind catalysts and substrates *via* hydrogen bonding interactions.^12^ In this case, sulfonated gold catalysts were strongly fixed in a guanidinium containing nanocage, and carboxylate-containing substrates were bound more weakly *via* the remaining guanidine units, providing the ability to pre-organize the substrates and the catalysts within the microenvironment. In the gold-catalyzed cyclization of carboxylate-containing substrates this pre-organization resulted in higher reaction rates. More recently, this guanidinium-functionalized nanosphere was used to accelerate reactions *via* a dinuclear mechanism, such as dinuclear Cu(i)-catalyzed cyclization^13^ and ruthenium-catalyzed water oxidation.^14^ These examples demonstrate that the generation of a high local concentration by encapsulation of multiple transition metal catalysts (and/or substrates) is an interesting new tool to control catalyst properties. Note that these examples have reported the encapsulation of transition metal catalysts that are connected *via* flexible linkers or *via* weak non-covalent interactions in the cavity, facilitating the catalysis due to the confinement effect of the cavity. However, similar nanospheres in which the metal catalysts are rigidly fixed and physically isolated on the rims of the cage windows^15^ and whether the windows could present the confinement effect in catalysis have not been well explored yet.

**Fig. 1:**
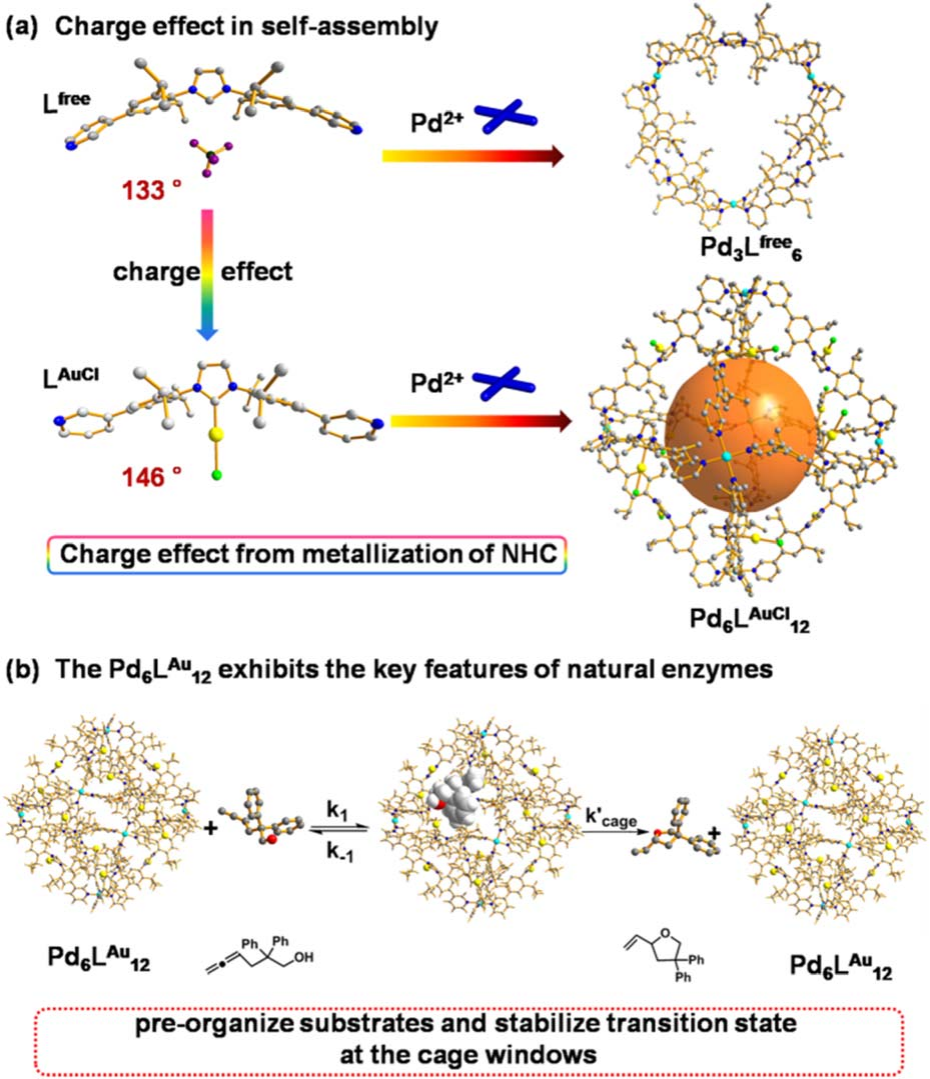
(a) Charge effect of rigid NHC metallization-triggered supramolecular configurations. (b) The Pd_6_L^Au^_12_ cage presents the features of enzymatic catalysis at the cage window for gold-catalyzed cyclization of allenol. The structures of L^free^, L^AuCl^ and Pd_6_L^AuCl^_12_ are their single crystal X-ray structures. The structure of Pd_3_L^free^_6_ is a modelled structure. Color coding: C: gray; N: dark blue; Au: yellow; Pd: light blue; Cl: bright green; B: dark green; F: violet; O: red; H: white.

Recently, Nitschke and co-workers reported a subcomponent tetrahedral cage that was functionalized with N-heterocyclic carbene (NHC) moieties in the middle of each edge.^16^ They reported a cage that contained NHC–gold complexes as part of the cage backbone and used it as a template to generate gold nanoparticles. Other literature research^17,18^ reported the construction and host guest studies of cages containing NHC moieties. However, the catalytic properties of these formed cages containing NHC were not explored. In this contribution, we report an example wherein the catalytic region is extended from the cage cavity to the windows on the cage surface area by the Pd_6_L^AuCl^_12_ nanospheres that contain rigidly fixed and physically isolated NHC–AuCl moieties at the cage window, and investigate its confinement effect of the windows and enzymatic catalytic behavior in catalysis (Fig. 1). These types of NHC–gold complexes have previously been explored in cyclization reactions^19^ of alkynes,^20^ allenes^21^ and alkenes,^22^ and then we set out to assess the catalytic performances of the Pd_6_L^Au^_12_ cage in gold-catalyzed cyclization reactions. Application of Pd_6_L^Au^_12_ nanospheres in the cyclization of allenol and hex-4-ynoic acid shows enhanced activity and selectivity. In addition, the physically separated complexes cannot form dinuclear complexes, making the Pd_6_L^Au^_12_ nanospheres more stable, especially under hydrous conditions. With this cage we have a clear example which shows that catalysis can favorably take place at the cage windows, and the systems display Michaelis–Menten kinetics, a feature also found in enzymatic catalysis.

## Results and discussion

### Synthesis and characterization of ligands and cages

The NHC-functionalized ditopic ligand was designed by embedding the rigid NHC moiety between two 3-pyridyl groups to allow coordination to palladium required for the self-assembly, leading to structures in which the NHC moiety is implemented in a rigid fashion. The pure ditopic pyridyl ligand was obtained in 2 steps with an overall yield of 85% (Fig. S1–S10†) and will be indicated as L^free^ in this paper to emphasize that it is metal free. Needle-shaped crystals of L^free^ were obtained from vapor diffusion of diethyl ether into a solution of L^free^ in acetonitrile over two weeks. The crystal structure of L^free^ shows that the ligand adopts a concave bending mode in which the imidazolium ring points inward, and the two neighboring aromatic rings display a bend angle of 133° (Fig. 4a, Tables S4 and S5†).

After treating the NHC-based ditopic pyridine compound with a stoichiometric amount of Au(tht)Cl, the gold complex of the ligand was obtained, which is denoted as L^AuCl^ (Fig. S11–S16†). Slow diffusion of isopropyl ether into the acetonitrile solution of L^AuCl^ led to square-shaped crystals. The colorless crystal of L^AuCl^ allowed solving the solid-state structure by SCXRD. The structure shows that pyridine groups were oriented differently when compared to that of L^free^ and the bend angle is rather different (146° compared to 133°), as a result of the coordination of gold on the imidazolium ring (Fig. 4a, Tables S6 and S7†). The properties of both L^free^ and L^AuCl^ in solution state were investigated by multiple spectroscopic NMR experiments and high-resolution mass spectrometry (HRMS) (all the spectral details can be found in the ESI†).

**Fig. 2:**
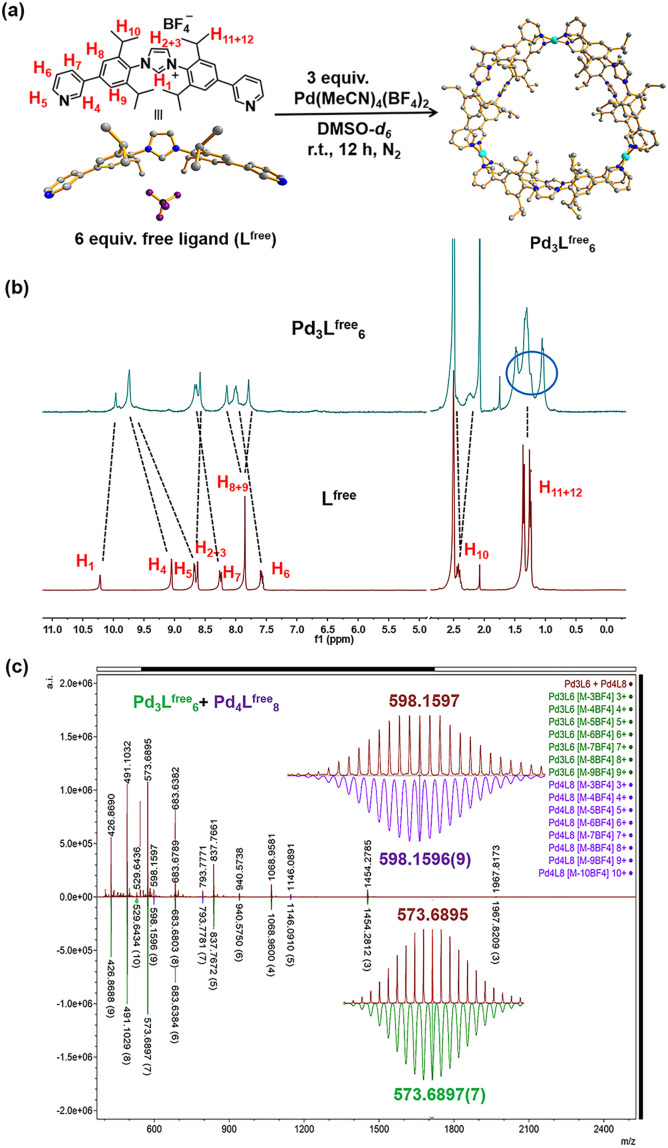
Synthesis and characterization of the gold-free cage (Pd_3_L^free^_6_). (a) Synthesis of the self-assembly of the gold-free cage (Pd_3_L^free^_6_). The structure of L^free^ is the SCXRD structure and the structure of Pd_3_L^free^_6_ is a modelled structure. Color coding: C: gray; N: dark blue; Pd: light blue; B: dark green; F: violet. (b) ^1^H NMR of the Pd_3_L^free^_6_ and free ligand (L^free^) in DMSO-*d*_6_ (at 298 K). (c) HR-CSI-MS of the gold-free cage (Pd_3_L^free^_6_ with minimal Pd_4_L^free^_8_) (red) and simulated isotopic distribution of Pd_3_L^free^_6_ [M-3BF_4_^−^]^3+^ (green) and Pd_4_L^free^_8_ [M-4BF_4_^−^]^4+^ (purple) in CH_3_CN.

These two ditopic ligands were used to make coordination cages by self-assembly using palladium as the metal source. A solution containing L^free^ and Pd(MeCN)_4_(BF_4_)_2_ with a ratio of 2 : 1 in DMSO-*d*_6_ (Fig. 2a) was stirred in a N_2_ atmosphere. After stirring vigorously for 12 h at 298 K, the light-yellow solid was collected by precipitation in excess amount of diethyl ether. Analysis of the compound by various techniques showed that the self-assembled Pd_3_L^free^_6_ cage was formed. The product was fully characterized by NMR spectroscopy and HR-MS (Fig. S17–S37†).

The relative shifts and broadened resonances of the ligand backbone in the ^1^H NMR spectrum indicated the coordination between L^free^ and palladium precursor (Fig. 2b). Compared to that of L^free^, the signals of pyridine protons H_4_ (Δ*δ* = −0.66 ppm; Fig. 2b) and H_5_ (Δ*δ* = −1.06 ppm; Fig. 2b) presented typical shifts, indicating the coordination with Pd^2+^ in line with the cage formation.^23^ The aromatic protons H_8_ (Δ*δ* = −0.30 ppm; Fig. 2b) and H_9_ (Δ*δ* = 0.06 ppm; Fig. 2b) no longer produced an identical chemical shift but split into two singlets and shifted individually, demonstrating that these protons resided in different chemical environments, because the neighboring aromatic rings could not be rotated due to the rigidity of the cage structure. Apparently, the aromatic rings are no longer able to rotate rapidly on the NMR time scale, due to the steric hindrance and rigidity experienced within the structure. Notably, the resonance signals of the isopropyl CH protons H_10_, H_11_ and H_12_ in the ^1^H NMR spectra of the cage were also split, clearly indicating the distinct chemical environment.

Diffusion-ordered NMR spectra (DOSY) in DMSO-*d*_6_ at 298 K exhibited a clear single narrow band around log *D* = −10.078 (*R*_H_ ≈ 2.61 nm, according to the Stokes–Einstein equation) (Fig. S22†). The composition of the Pd_3_L^free^_6_ cage was clearly proven by high-resolution cold spray ionization mass spectrometry (HR-CSI-MS) in acetonitrile. A single set of species with various charged states (3+, 4+, 5+, 6+, 7+, 8+ and 9+) was observed (Fig. 2c). All signals were assigned to a structure having the formula Pd_3_L^free^_6_(BF_4_)_12_ with a progressive loss of BF_4_^−^ counterions during the MS measurement (Fig. S23†). Clearly, for all of the charged states, the experimental values precisely matched with the calculated isotopic distributions (Fig. S24–S37†).

According to the structural information, we propose that this cage holds a double crown ring by combining two rings connected *via* three Pd^2+^ metal nodes. The modeled structure shows an average diameter of 26 Å (Fig. 4b), which is in line with that obtained from the DOSY NMR spectra (Fig. S22†). The top view of the structure is displayed showing the rings connected to the palladium nodes (Fig. 4b). The structure consists effectively of three small rings (Pd_2_L^free^_2_) connected to one another. The torsion angle of L^free^ in the coordination state is 139°, which is larger than its angle in the free state. The structure contained three Pd_2_L^free^_2_ rings, showing the symmetry of Pd_3_L^free^_6_, which also matched the splitting of isopropyl protons in the NMR results (Fig. 2b). A similar structure of trigonal prismatic Pd_3_L_6_ containing PF_6_^−^ was reported as the basic building block for the formation of crystal mesoporous supramolecular materials.^17^ The crystals of Pd_3_L_6_(PF_6_)_12_ crystallize in the monoclinic space group. Its framework contains three Pd^2+^ metal centers and six ligands. The calculated structure presents a triangular prism skeleton with three small rings of Pd_2_L_2_. The structural analysis of Pd_3_L_6_(PF_6_)_12_ sufficiently verifies our modeling of Pd_3_L^free^_6_(BF_4_)_12_.

**Fig. 3:**
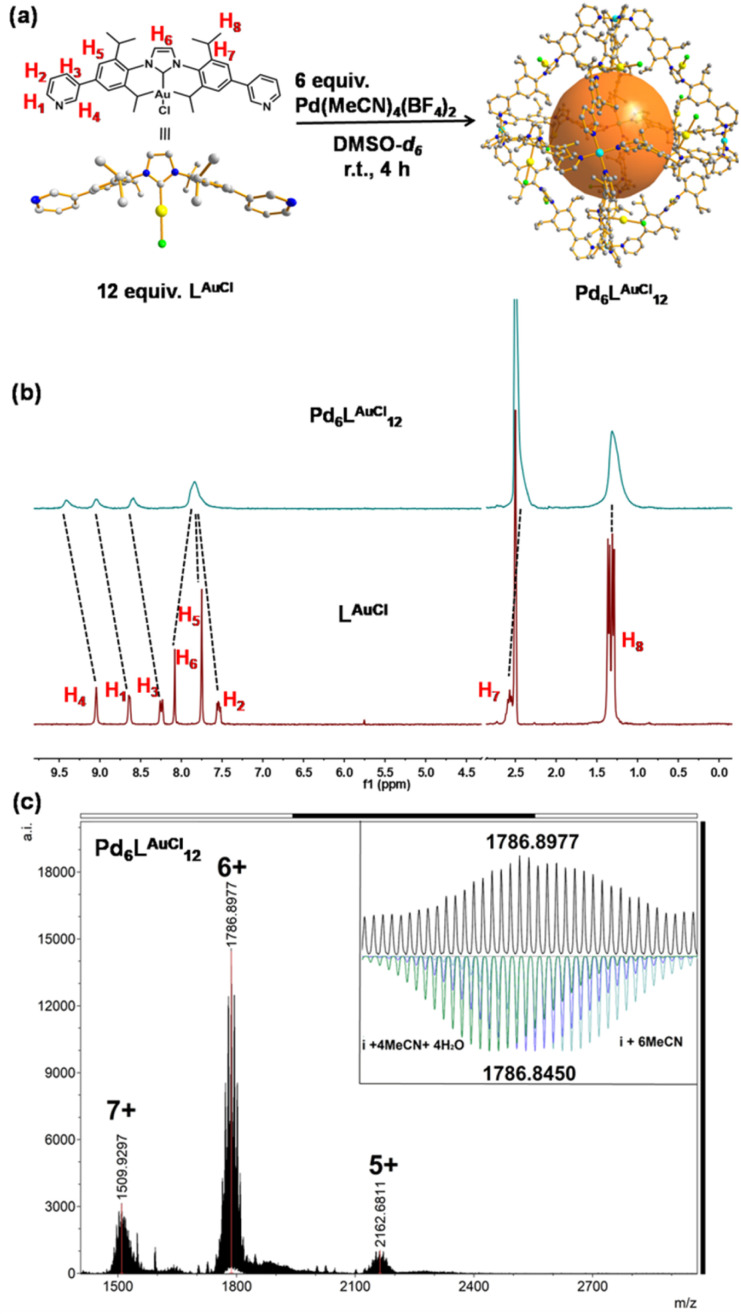
Synthesis and characterization of Pd_6_L^AuCl^_12_. (a) Synthesis of the self-assembly of Pd_6_L^AuCl^_12_. The structures of L^AuCl^ and Pd_6_L^AuCl^_12_ are obtained by SCXRD. (b) ^1^H NMR of L^AuCl^ and the Pd_6_L^AuCl^_12_ in DMSO-*d*_6_ (at 298 K). Color coding: C: gray; N: dark blue; Au: yellow; Pd: light blue; Cl: bright green; B: dark green; F: violet; O: red; H: white. (c) HR-CSI-MS of Pd_6_L^AuCl^_12_ and simulated isotopic distribution of charged species (+6) containing solvent molecules.

**Fig. 4:**
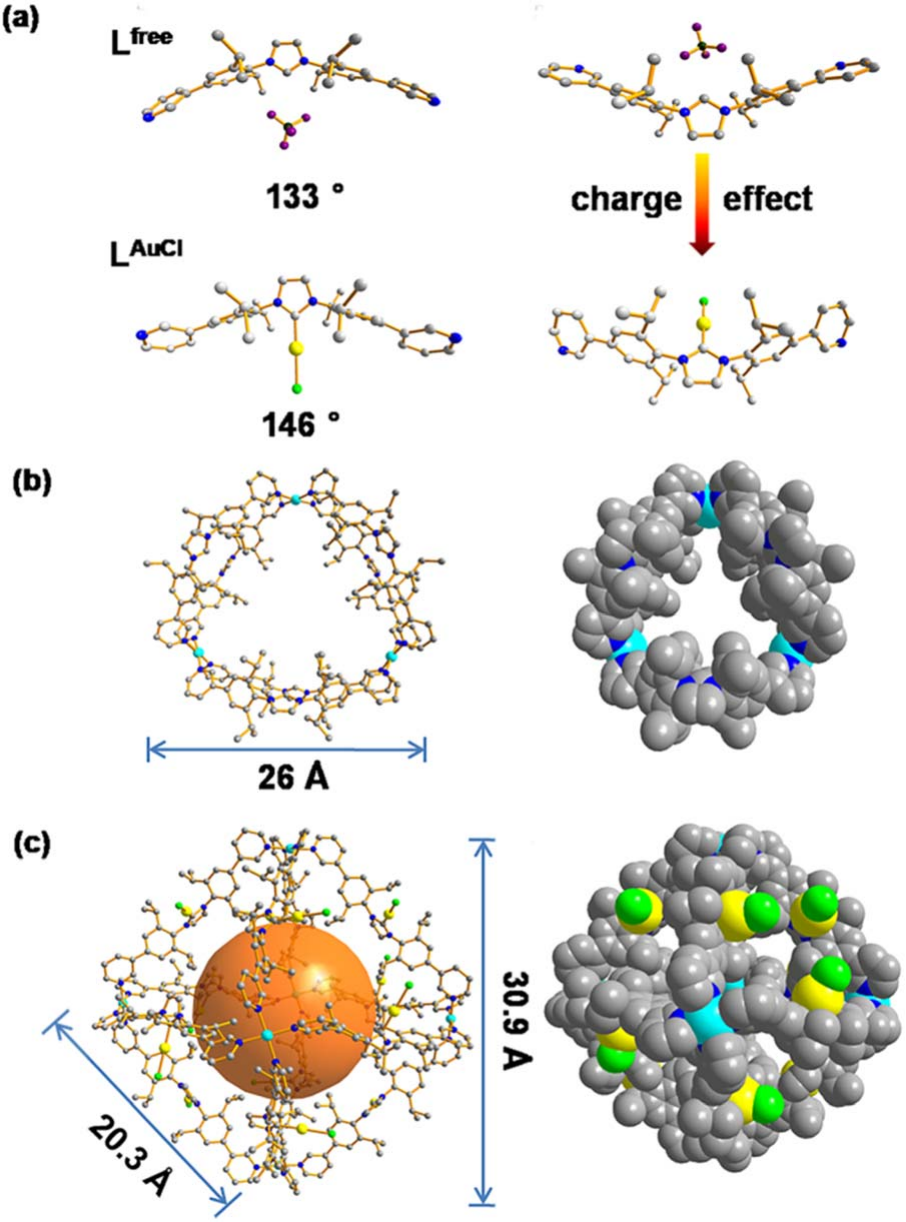
(a) Single crystal X-ray structures of L^free^ and L^AuCl^. (b) MD simulated structures of Pd_3_L^free^_6_, ball-and-stick and space-filling models. (c) Single crystal X-ray structures of Pd_6_L^AuCl^_12_, ball-and-stick model and space-filling models. Color coding: C: gray; N: dark blue; Au: yellow; Pd: light blue; Cl: bright green; B: dark green; F: violet.

A solution containing L^AuCl^ and palladium salt in DMSO-*d*_6_ was stirred at room temperature for 4 hours (Fig. 3a). A single set of slightly broadened signals was observed in the ^1^H NMR spectra (Fig. 3b and S38†). All the resonance signals were assigned with the help of 2D^1^H COSY NMR (Fig. S40–S42†). In the^1^H NMR spectrum, the resonance signals of the pyridine signals were shifted [Δ*δ* (H_1_) = −0.41 ppm; Δ*δ* (H_2_) = −0.29 ppm; Δ*δ* (H_3_) = −0.33 ppm; Δ*δ* (H_4_) = −0.36 ppm], in line with the coordination with the palladium center^23^ (Fig. 3b). Moreover, the resonance signals of isopropyl groups [H_8_] in the cage structure did not shift too much but rather broadened with respect to the signals of L^AuCl^ (Fig. 3b), indicating that a highly symmetric cage had formed. DOSY showed a single band at log *D* = −10.10 (*R*_H_ ≈ 2.75 nm, according to the Stokes–Einstein equation) (Fig. S43†), consistent with the cage size measured from the single crystal structural analyses (Fig. 4c). HR-CSI-MS measurements for the solution of the Pd_6_L^AuCl^_12_ cage in acetonitrile supported the composition of the desired cage, as a set of prominent peaks with different charge states were observed at 2162.6811, 1786.8977, and 1509.9297; this result precisely agreed with the simulated values of Pd_6_L^AuCl^_12_ with the respective amount of counterions (Fig. S44–S46†). Furthermore, a series of peaks with a certain amount of additional coordinated solvent molecules could also be recognized (Fig. S45†). The simulated isotopic distribution of the +6 charged species (Fig. 3c) clearly shows that the cage structure contains some solvent molecules, which results in partly overlapping peaks. By slow diffusion of diethyl ether vapor into an acetonitrile solution of Pd_6_L^AuCl^_12_ over three weeks, rhombus-shaped single crystals of the Pd_6_L^AuCl^_12_ cage were obtained.

Single-crystal X-ray diffraction (SCXRD) analysis shows that Pd_6_L^AuCl^_12_ crystallized in a triclinic space group (Fig. 4c, Tables S8 and S9†). The Pd_6_L^AuCl^_12_ cage presents a highly symmetric octahedron geometry, which contains six Pd^2+^ cations that occupy the vertices and are linked together by twelve L^AuCl^ ligands as the edges. For all the NHC–gold species embedded in Pd_6_L^AuCl^_12_, the bending mode is similar to the free style of L^AuCl^. However, comparing with the bend angle of L^AuCl^ (146°) in the free state of the structure, this bend in the coordination state has an even larger torsion angle (162°). We hypothesize that this larger torsion angle is derived from the twist of L^AuCl^ during its coordination to palladium. In the solid state, all the gold centers reside at the windows of the Pd_6_L^AuCl^_12_ cage instead of in the cavity. The distance between the gold atoms and the neighboring one is 10 Å. It indicates that the distance of gold–gold in the crystal structure is quite large compared to the actual distance for the aurophilic interactions between two gold atoms (approximately 3 Å), as reported in the literature.^24^ Therefore, the gold centers here in the Pd_6_L^AuCl^_12_ cage are rather isolated and exposed at the window as observed in the solid-state structure.

A similar structure of self-assembly Pd_6_L_12_ (NO_3_^−^ salt), containing NHC–AuI moieties in ligands, was also reported and applied for anion (PF_6_^−^ and BF_4_^−^) encapsulations.^18^ The crystals crystallize in a triclinic space group. The highly symmetric octahedral structure contains six Pd^2+^ metal centers and twelve ligands. The bend angle (174.1°) in the coordination state is closer to linearity than that in the Pd_6_L^AuCl^_12_ cage (162°).

### Modeling of the cage structures

**Fig. 5:**
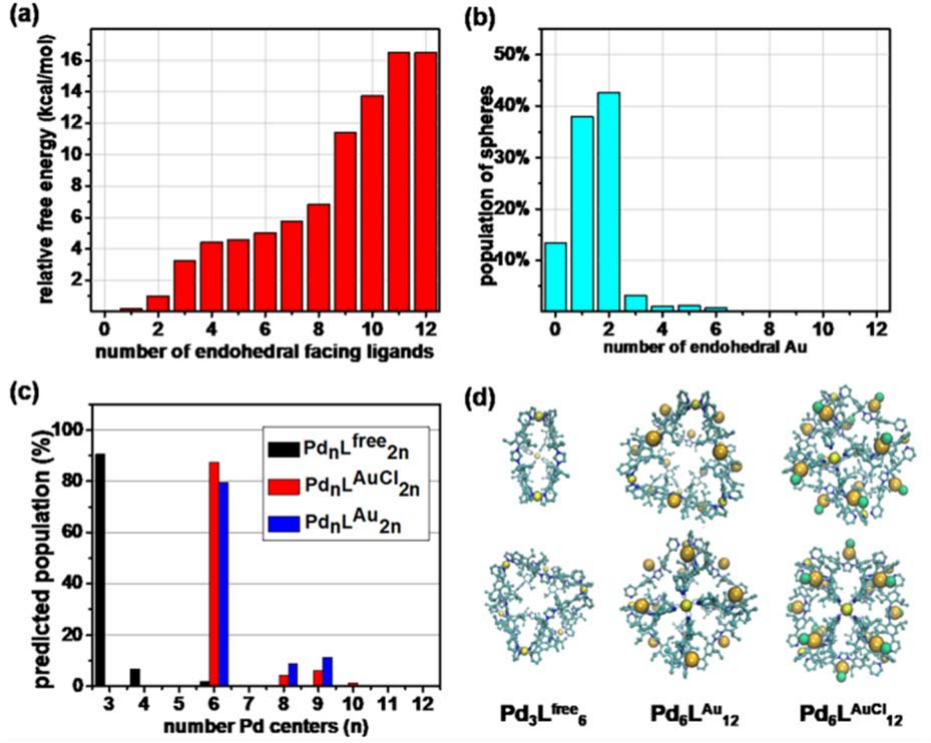
(a) Relative free energy and (b) degeneracy-corrected population estimates for Pd_6_L^Au^_12_ cages with a number of endohedral-facing ligand gold centers. (c) The predicted population of the different Pd_*n*_L_2*n*_ species formed from L^free^, L^AuCl^, and L^Au^, determined by MD simulations alongside renderings of Pd_3_L^free^_6_, Pd_6_L^Au^_12_, and Pd_6_L^AuCl^_12_ cages. (d) Renderings of majority cage products from two orthogonal viewpoints, with hydrogen atoms being excluded for clarity.

As such, Pd_6_L^AuCl^_12_ contains a unique inversion of exohedral-facing gold catalysts that are fixed on the edges of the windows and exhibits a high symmetry in the solid state (Fig. 4c). While the crystal structure shows a uniform and highly symmetric organization of the gold faces in the solid state, it is known that crystallization preferentially isolates highly symmetric structures.^25^ To understand the possible solution-state structure of Pd_6_L^AuCl^_12_ we adapted molecular dynamics simulations (MD)^26^ to assess the number of endohedral (*i.e.*, inward facing) or exohedral (*i.e.*, solvent-facing) facing ligands in Pd_6_L^AuCl^_12_. Model cages featured a varying number of endohedral-facing gold centers, where the relative energy of each configuration (Fig. 5a) could be directly used for computing the distribution of ligand orientations (Fig. 5b). The larger number of configurations accessible by spheres featuring some endo- or exohedrally facing ligands results in an entropic preference for mixed cages (Fig. 5a). Combined with the relatively low energy penalty (<2 kcal mol^−1^) of including a few endohedral ligands, our model predicts that the plurality of the Pd_6_L^AuCl^_12_ cages features 1–2 endohedral-facing gold centers (Fig. 5a), while a majority of gold sites (87%) (Fig. 5b) are exohedral and remain well separated, as confirmed by UV-vis spectroscopy^11^ (Fig. S49†) owing to the steric bulk of the cage. This was further confirmed by the crystal structure. Noticeably, gold centers in both the Pd_6_L^Au^_12_ and the Pd_6_L^AuCl^_12_ cages are located on the periphery of the cage windows rather than in the cavity itself, exposing a high concentration of isolated metal centers.

As we observed by CSI-HRMS, L^free^ forms Pd_3_L^free^_6_ double-crown ring assemblies, while gold-containing building blocks afford Pd_6_L^AuCl^_12_ octahedral cages (Fig. 2c and 3c). As reported by Fujita, a small difference in the bend angle of the bidental ligand block may result in the formation of different nanostructures.^8^L^free^ exhibits a concave binding mode with a bend angle of 133°, while L^AuCl^ adopts a convex binding mode with an angle of 146°, probably because pyridine groups in L^AuCl^ are flipped (Fig. 4a). The torsion angle of L^free^ (139°) is smaller than that of L^AuCl^ (162°). As both windows in the two cages feature similar triangular geometry, we hypothesize that this torsion angle change drives the divergent outcomes for the self-assembly of these ligands.

Intrigued by these structural differences, we employed MD^26^ simulations to understand the topological preference of L^free^ and gold-containing congeners, L^Au^ and L^AuCl^ (Fig. 5d). While the ligands feature a similar molecular structure, we observed significant differences in the charge distribution between the metalized and non-metalized ligands in their coordination states (Fig. S47†). Surprisingly, L^free^ (coordination state) possesses higher charge densities (*i.e.*, increased polarization) on the *ortho* positions of the pyridine groups compared to its gold-complexed congeners (L^AuCl^ and L^Au^) in self-assembled structures. This charge difference may account for the divergent self-assembly outcomes we have observed.

In line with our CSI-HRMS measurements (Fig. 2c), our MD^26^ simulations predict that L^free^ favorably self-assembles into double-crowned Pd_3_L^free^_6_ assemblies, with a minor population of the Pd_4_L^free^_8_ (Fig. 5c). While, the Pd_4_L^free^_8_ could not be detected by^1^H NMR (Fig. 2b) or DOSY experiments (Fig. S22†), the combination of CSI-HRMS and computational evidence supports the Pd_4_L^free^_8_ at low concentrations. In contrast, MD simulations of L^AuCl^ and L^Au^ predict the observed Pd_6_L^AuCl^_12_ and Pd_6_L^Au^_12_ octahedral cages as the dominant product of self-assembly (Fig. 5c).

### Cyclization of allenol (Pd_6_L^Au^_12_*vs.* NHC–Au^+^)

As we have already revealed, Pd_6_L^AuCl^_12_ creates a unique configuration featuring exposed and isolated gold centers on the edges of the cage. We anticipated that these structural characteristics may result in differences in catalysis compared to the mononuclear complex and also compared to previously reported structures in which the complexes are nearer to one another. Inspired by our recent precedents applied in gold-catalyzed cycloisomerizations, in which the gold complexes were furnished inside a well-defined cage *via* flexible linkers that exhibited aurophilic interactions and led to a highly enhanced reactivity, we started our studies of Pd_6_L^Au^_12_ in the cycloisomerization of allenol (1).^27^

All experiments were precisely carried out under the same benchmark reaction conditions described above and were performed in pre-dried deuterated acetonitrile under argon at room temperature for 25 h (Tables 1 and S1†). In the gold-catalyzed cycloisomerization of allenol (substrate 1), the catalytic result could in principle yield two products, the 5-membered ring (product 2) and the 6-membered ring (product 3). In all reactions, we found that the 5-membered ring (product 2) was the only product formed. With no surprise, the NHC–AuCl complex was found to have no reactivity, and even after pre-activation by AgBF_4_, the complex only displayed a low reactivity to generate product 2 in 30% yield. As expected, the Pd_6_L^AuCl^_12_ cage was completely inactive, in contrast to our previously reported flexible system, indicating that the gold complexes are well separated at the cage window, to prevent aurophilic interactions that were reported to be important in the flexible system, and the system did not give any conversions of allenol (substrate 1). Gratifyingly, the pre-activated Pd_6_L^Au^_12_ cage displayed a moderate yield of product 2 (57%) compared to the active monomeric NHC–Au^+^ catalyst, while the gold concentration in these experiments was the same and based on the ligands.

**Table 1:** Gold-catalyzed cyclization of allenol 1^a^,^b^

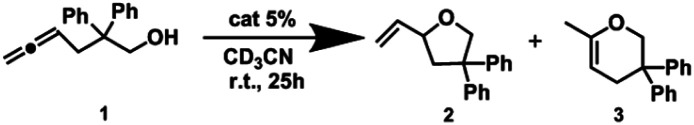				
Entry	Conditions	Conv.^b^ (%)	2^b^ (%)	3^b^ (%)
1	NHC–AuCl	0	0	0
2	NHC–Au^+^	30	30	0
3	Pd_6_L^AuCl^_12_	0	0	0
4	Pd_6_L^Au^_12_	57	57	0

a Reaction conditions: [1] = 50 μM, [AgBF_4_] = 2.5 μM, [NHC–AuCl] = 2.5 μM, [Pd_6_L^AuCl^_12_] = 2.5/12 = 0.21 μM.

b The catalytic results were calculated based on ^1^H NMR by using 1,3,5-trimethoxybenzene as an internal standard. The results are averages of 2 or 3 reactions.

Interestingly, when the catalytic reactions were performed in hydrous acetonitrile, we observed an even larger difference in reactivity between NHC–Au^+^ and Pd_6_L^Au^_12_ (Table S2†). The reactivity of the Pd_6_L^Au^_12_ cage was unaffected in the presence of water, providing a conversion of 57%, whereas catalysis with the NHC–Au^+^ complex dropped to only 13% (Table S2†). We considered monomeric NHC–Au^+^ to be deactivated by dimer formation under hydrous conditions. Spectroscopic analysis showed that hydroxy bridge dimeric complexes formed in the presence of water, in line with previous reports.^28^ The mixture of mononuclear and dinuclear gold species presented a ratio of 1 : 1 (Fig. S53†), as determined *via*^1^H NMR, and this ratio remained constant even after the addition of 50 μL of D_2_O (measured after 24 h, Fig. S54†). In contrast, the NHC–Au^+^ complexes only present in a monomeric form in an anhydrous acetonitrile system. Notably, the NHC–Au^+^ units in the Pd_6_L^Au^_12_ cage are restricted to a separated state, effectively blocking the formation of dimeric gold species. This explains the stability of NHC–Au^+^ units at the window of the cage and the associated higher reactivity in hydrous reaction environments.

To further evaluate the difference in the activity and stability between the Pd_6_L^Au^_12_ cage and NHC–Au^+^ complex, a kinetic analysis of the catalytic reaction was performed in subsequent batch reactions by the addition of a second batch of substrate. This was performed under both hydrous and anhydrous conditions (Fig. S50 and S51†). Under hydrous conditions, in the first reaction cycle, the Pd_6_L^Au^_12_ cage showed a much higher reactivity (TOF_ini_ = 1.02 h^−1^) than the monomeric analog (TOF_ini_ = 0.38 h^−1^), in line with the higher yield of product 2 obtained (Table 1). The curve of NHC–Au^+^ was flatter after 10 h than that of the Pd_6_L^Au^_12_ as the catalyst (Fig. S50†), in line with dimer formation which decreased the amount of active species. After 49 h, a second batch of substrate was added to test the catalytic performances in the second cycle. Although the rate displayed by the Pd_6_L^Au^_12_ cage (TOF_ini_ = 0.80 h^−1^) slightly decreased compared to that in the first cycle, it was still relatively high. Importantly, this rate was still higher than the rate observed for NHC–Au^+^ (TOF_ini_ = 0.24 h^−1^). This result demonstrated that the rigidity features of the Pd_6_L^Au^_12_ cage effectively blocked dimer formation and evidently stabilized the gold centers. Under anhydrous conditions, the difference in reactivity in kinetic rates between the Pd_6_L^Au^_12_ cage and NHC–Au^+^ was smaller (in the first cycle, TOF_ini_ = 1.02 h^−1^ for the Pd_6_L^Au^_12_ cage and TOF_ini_ = 0.50 h^−1^ for NHC–Au^+^). Additionally, in the experiment when a second batch of substrate was added, the rates for the two catalysts (TOF_ini_ = 0.88 h^−1^ for the Pd_6_L^Au^_12_ cage and TOF_ini_ = 0.42 h^−1^ for monomeric NHC–Au^+^) were nearly identical to those in the first batch (Fig. S51†). These observations in the experiments demonstrate the excellent stability and activity of the Pd_6_L^Au^_12_ catalyst derived from the rigidity of the cage configuration.

### Michaelis–Menten kinetics in the cyclization of allenol

**Fig. 6:**
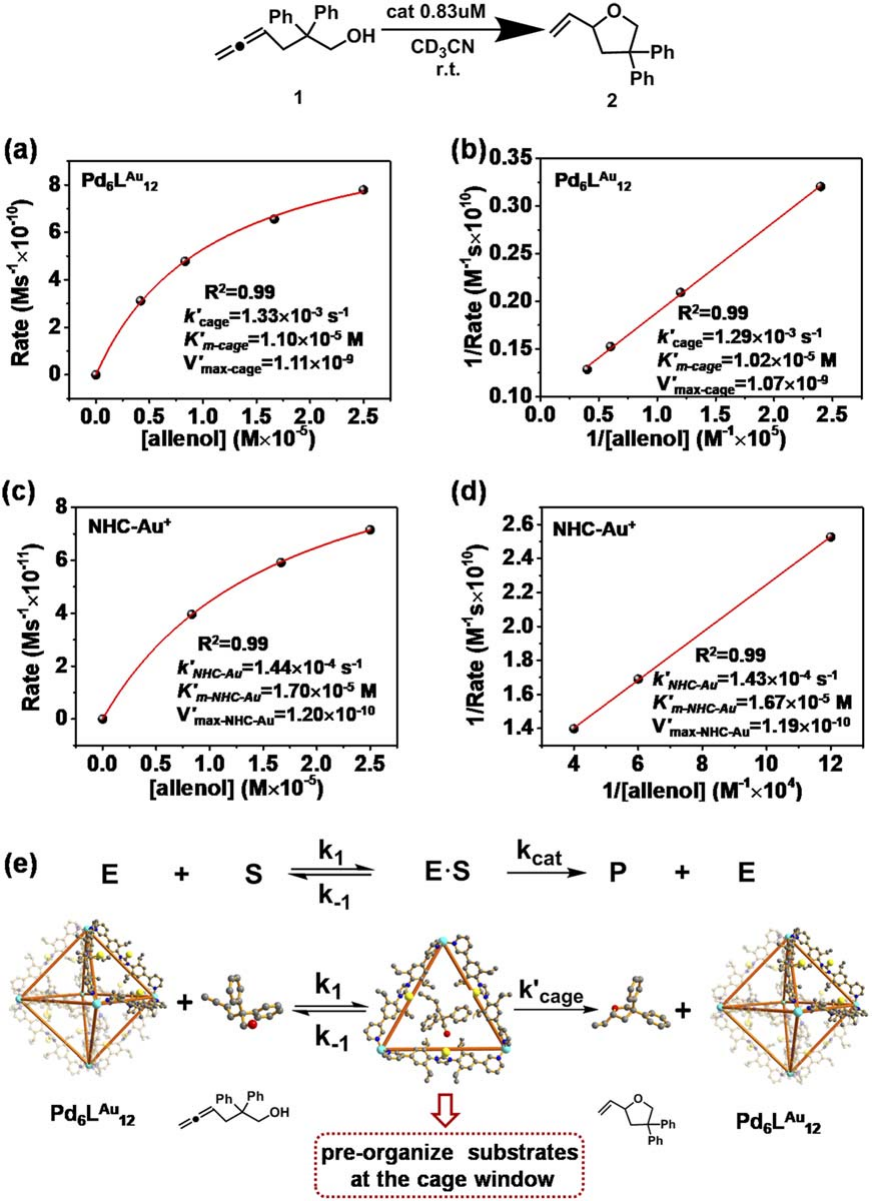
Kinetic experiments and implications in the cyclization reaction of allenol 1. (a) Kinetic analyses and (b) Lineweaver–Burk plot for the Pd_6_L^Au^_12_ cage. (c) Kinetic analyses and (d) Lineweaver–Burk plot for NHC–Au^+^. (e) The formula of the Michaelis–Menten model and the Pd_6_L^Au^_12_ cage (using the SCXRD structure as the model) exhibits the features of enzymatic catalysis in the cyclization of allenol. Color coding: C: gray; N: dark blue; Au: yellow; Pd: light blue; O: red; H: white.

We have demonstrated that this rigid catalytic system (Pd_6_L^Au^_12_) exhibited strong stability and excellent reactivity; thus, to gain a better understanding of its essential role during catalysis, Michaelis–Menten enzymatic rate constant studies^29,30^ were performed. The kinetic experiments were conducted using the method of initial rates by utilizing Pd_6_L^Au^_12_ or NHC–Au^+^ as the catalyst (Fig. 6a–d, S55–S57, S69 and S70†). The dependence on the substrate concentration showed a saturation behavior that could be linearized by plotting the double reciprocal of the substrate concentration and the rate. The Michaelis–Menten parameter 
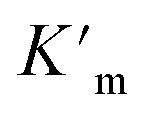
 for Pd_6_L^Au^_12_ was determined to be 1.02 × 10^−5^ M, while the 
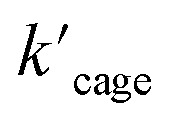
 (Michaelis–Menten enzymatic rate constant) was found to be 1.29 × 10^−3^ s^−1^ from the Lineweaver–Burk plot (Fig. 6b). These results are consistent with an overall Michaelis–Menten-type mechanism.

As expected, Pd_6_L^Au^_12_ shows a higher *k*_cat_ and a larger *V*_max_ than NHC–Au^+^ (Fig. 6a–d), which is in line with the higher conversion (Table 1). The Michaelis–Menten parameter *K*_m_
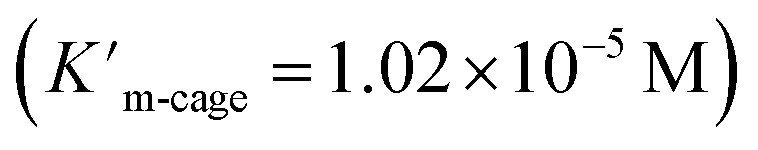
 for Pd_6_L^Au^_12_ is lower than that for NHC–Au^+^
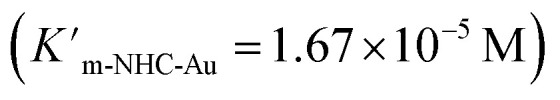
, indicating that the substrate is bound more strongly to Pd_6_L^Au^_12_.

Next, we sought experimental evidence for the binding of the substrate to the cage through NMR experiments. We conducted a set of control experiments to monitor the chemical shifts of Pd_6_L^Au^_12_ upon addition of different equivalents of allenol into the Pd_6_L^Au^_12_ solution (Fig. S73†). It was noted that the NHC–Au(i) five-membered rings in the ligands are placed in-plane of the face of the octahedron (crystal structure in Fig. 4c). In such a case, Au (i) in each NHC–Au (i) five-membered ring has two possibilities of pointing towards the inside of the triangle face, producing stereoisomers. As the rotation of the five-membered ring would be fast, the stereoisomers are not observed by NMR at room temperature. It is consistent with the NMR spectra of the Pd_6_L^Au^_12_ binding substrate (Fig. S73†). In line with the binding of the substrate, the signal of Pd_6_L^Au^_12_ exhibits upfield chemical shifts (approximately 0.01 and 0.02 ppm) in the aromatic region. In addition, the CH proton of the isopropyl group of Pd_6_L^Au^_12_ shows an upfield shift of 0.1 ppm. Importantly, a similar set of NMR experiments using the mononuclear NHC–Au^+^ complex showed no change in the chemical shifts of the NHC–Au^+^ in the presence of the substrate (Fig. S74†). It demonstrates that the substrate binds to Pd_6_L^Au^_12_, showing the relevance of the molecular cage structure. Taking all the results together, Pd_6_L^Au^_12_ possesses the ability of substrate pre-organization at the cage windows, which leads to more efficient catalytic conversion. The comparison of 
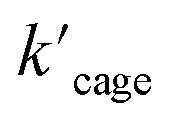
 and 
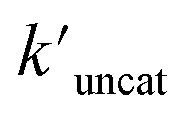
 (the rate constant for the uncatalyzed reaction, 
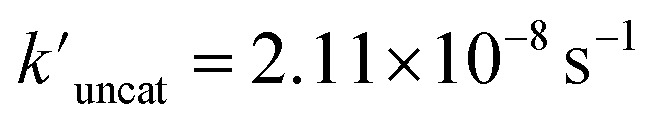
= 2.11 × 10^−8^ s^−1^) was also taken into consideration and provided an overall acceleration (6.11 × 10^4^) (Fig. S58†) comparable with the previously reported supramolecular catalysis.^30^

### Cyclization of hex-4-ynoic acid (Pd_6_L^Au^_12_*vs.* NHC–Au^+^)

Next, we further extended the catalytic studies in the intramolecular cyclization of hex-4-ynoic acid (substrate 4)^31^ (Tables 2 and S3†) to probe the cage effect on the selectivity of the reaction. Without pre-activation by AgBF_4_ to abstract chloride, both the NHC–AuCl (Table 2, entry 1) and the Pd_6_L^AuCl^_12_ cage (Table 2, entry 3) were inactive. When NHC–AuCl was treated with AgBF_4_, a full conversion of substrate 4 was achieved in 10 minutes. Both the 5-membered ring product 5 (40%) and 6-membered ring product 6 (60%) were formed in a ratio of 5/6 = 0.7 (Table 2, entry 2). Interestingly, after the pre-activation of Pd_6_L^Au^_12_ this catalyst also led to a full conversion of substrate 4 in 10 min. However, the yield of the 5-membered ring product 5 increased to 54%, whereas the yield of product 6 was 45%. In the presence of the Pd_6_L^Au^_12_ cage, the 5/6 ratio was calculated as 1.2 (Table 2, entry 4), compared to 0.7 found for the monomeric catalyst. Clearly, the improved ratio of 5/6 is controlled to some extent by the space constraint of the cage windows.

**Table 2:** Gold-catalyzed cyclization of hex-4-ynoic acid^a^

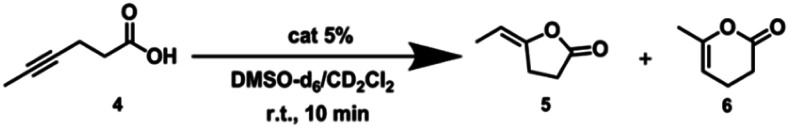					
Entry	Conditions	Conv.^b^ (%)	5^b^ (%)	6^b^ (%)	5 : 6
1	NHC–AuCl	0	0	0	—
2	NHC–Au^+^	100	40	60	0.7
3	Pd_6_L^AuCl^_12_	0	0	0	—
4	Pd_6_L^Au^_12_	100	54	45	1.2

a Reaction conditions: [4] = 50 μM, [AgBF_4_] = 2.5 μM, [NHC–AuCl] = 2.5 μM, [Pd_6_L^AuCl^_12_] = 2.5/12 = 0.21 μM, DMSO-*d*_6_/CD_2_Cl_2_ = 1 : 3, the total reaction volume was 0.6 mL, 298 K.

b Catalytic reactions were calculated based on ^1^H NMR by using durene as an internal standard, and all the reactions were performed for 2–3 runs.

### Michaelis–Menten kinetics in the cyclization of hex-4-ynoic acid

**Fig. 7:**
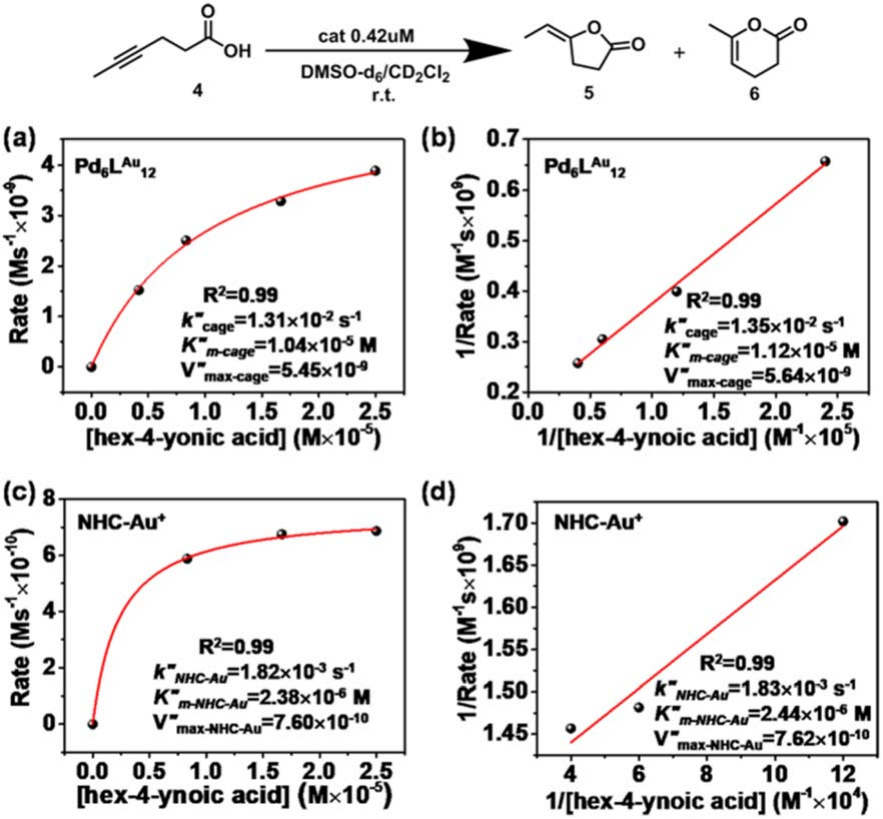
Kinetic experiments and implications in the cyclization reaction of hex-4-ynoic acid. (a) Kinetic analyses and (b) Lineweaver–Burk plot for the Pd_6_L^Au^_12_ cage. (c) Kinetic analyses and (d) Lineweaver–Burk plot for NHC–Au^+^.

Also for the cyclization of hex-4-ynoic acid, we monitored the reaction kinetics *via* the method of initial rates and modeled the results by Michaelis–Menten kinetic analysis^29,30^ (Fig. 7a–d, S59–S67, S71 and S72†). The Pd_6_L^Au^_12_ cage also displayed Michaelis–Menten kinetic behaviour for this substrate (Fig. 7a and b). The corresponding plotting of the double reciprocal of the substrate concentration and the rate afforded an estimation for 
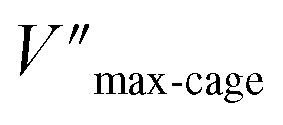
 (5.64 × 10^−9^) and 
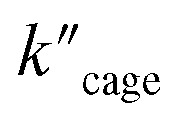
 (1.35 × 10^−2^ s^−1^) 
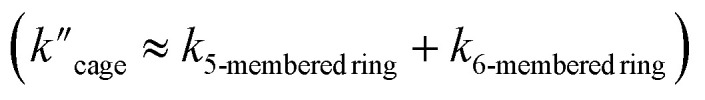
 (Fig. 7b and Fig. S65–S67†). We observe that the *k*_cat_ and *V*_max_ for both products 5 and 6 are estimated as the same, whereas the *K*_m_ for product 5 is lower than that of 6 when utilizing Pd_6_L^Au^_12_ as the catalyst (Fig. S66 and S67†). The data indicate that the cage effect has an influence on the affinity of the intermediate that leads to product 5, which is in line with the selectivity shown in Table 2.

Pd_6_L^Au^_12_ shows a higher *k*_cat_ and a larger *V*_max_ than NHC–Au^+^ (Fig. 7a–d) as shown in the benchmark reaction of allenol cyclization, also in line with the high conversion in Table 2. Interestingly, Pd_6_L^Au^_12_ shows a larger *K*_m_ (1.12 × 10^−5^ M) than NHC–Au^+^ (2.44 × 10^−6^ M), which indicates that the substrate is more strongly bound to NHC–Au^+^ than the cage, whereas Pd_6_L^Au^_12_ shows a higher *V*_max_ (5.64 × 10^−9^) than NHC–Au^+^ (7.62 × 10^−10^). The data suggest a transition state stabilization effect by utilizing Pd_6_L^Au^_12_. The comparison of 
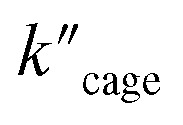
 and 
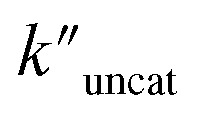
 (the rate constant for the uncatalyzed reaction, 
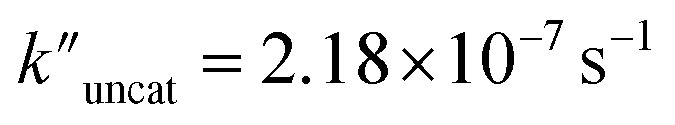
) was also taken into consideration, showing an enhanced order of 10^4^ (6.19 × 10^4^) (Fig. S68†).

## Conclusions

In summary, we report an example to extend the catalytic region from the cage cavity to the windows by utilizing Pd_6_L^Au^_12_ cages that contain physically isolated and exposed gold centers fixed rigidly at the windows. Pd_6_L^Au^_12_ displayed enhanced reactivity and high selectivity compared to the monomeric analogue in cyclization reactions. The rigidly fixed gold complexes with the Pd_6_L^Au^_12_ cage cannot interact with each other, thus prohibiting the formation of hydroxy bridged dinuclear complexes, which usually represent a dead end. As a result, the Pd_6_L^Au^_12_ cage is a more stable catalyst under hydrous conditions compared to the monomeric analogue that was demonstrated to form the less active dinuclear complexes. The Pd_6_L^Au^_12_ cage is able to bind and thus pre-organize allenol at the windows of the cage as judged by NMR experiments. Pd_6_L^Au^_12_ shows another key enzymatic feature of transition state stabilization in the cyclization of hex-4-ynoic acid. These lead to Michaelis–Menten-type kinetics when Pd_6_L^Au^_12_ is used as the catalyst, typical features of enzymatic catalysis. This substrate pre-organization and transition state stabilization explain the higher reaction rate displayed by the Pd_6_L^Au^_12_ cage catalyst. This is an example of how a molecular cage presents confinement effects at the windows, and we also report that with these design elements enhanced reactivity, selectivity and stability can be achieved. This contribution extends the catalytic region of the supramolecular cage from the cavity to the windows on the cage surface area, paving a new avenue to innovate bioinspired catalysts, as well as enable deeper understanding of supramolecular catalysis.

## Author contributions

Meiling Xu, Xu Jing, Chunying Duan and Joost N. H. Reek conceived the project and designed the experiments. Meiling Xu carried out the experiments, collected and interpreted the data. David A. Poole III contributed the calculations. Eduard O. Bobylev contributed the mass experiments. Jinguo Wu and Cheng He solved and refined all the X-ray single-crystal structures. Meiling Xu, Bin Sun, David A. Poole III, Eduard O. Bobylev, Cheng He, Chunying Duan and Joost N. H. Reek cowrote the paper. All authors discussed the results and commented on the manuscript.

## Conflicts of interest

There are no conflicts to declare.

## Supplementary Material

SC-014-D3SC02998K-s001

SC-014-D3SC02998K-s002
