# Supplementary material for: Broadening the catalytic region from the cavity to windows by M6L12 nanospheres in cyclizations
Source: Chem Sci. 2023 Sep 26;14(42):11699–707. doi: 10.1039/d3sc02998k (PMC10619639; doi:10.1039/d3sc02998k)
Supplement: SC-014-D3SC02998K-s001 [file SC-014-D3SC02998K-s001.pdf]

Electronic Supplementary Material (ESI) for Chemical Science.
This journal is © The Royal Society of Chemistry 2023

Supporting Information

Broadening the catalytic region from the cavity to windows by the M_6L_{12} nanospheres in cyclizations

Meiling Xu,[a] Bin Sun,[b] David A. Poole III,[b] Eduard O. Bobylev,[b] Xu Jing,*[a] Jinguo Wu,[a] Cheng He,[a] Chunying Duan,*[a] and Joost N. H. Reek.*[b]

[a]State Key Laboratory of Fine Chemicals, Dalian University of Technology, Dalian 116024, P. R. China.

[b]Homogeneous, Supramolecular and Bio-Inspired Catalysis, Van't Hoff Institute for Molecular Sciences, University of Amsterdam, Science Park 904, Amsterdam 1098 XH, The Netherlands.

Email: *xjing@dlut.edu.cn, *cyduan@dlut.edu.cn, *j.n.h.reek@uva.nl

Supplementary methods :

All the reactions were carried out under the atmosphere of Ar/N_2 unless otherwise stated. Solvents were distilled by conventional methods. All reagents were purchased from commercial suppliers and used without further purification. NMR spectra were measured on the Mercury 300, Bruker AMX 400, and Inova 500, with working frequencies of 300/400/500 MHz for ^{1}H NMR, and 75/100/125 MHz for ^{13}C NMR, respectively. ^{1}H NMR spectra were referenced to solvent residual signal (2.50 ppm for DMSO-d_6, 1.94 ppm for CD_3CN and 5.32 ppm for CD_2Cl_2). ^{13}C NMR chemical shifts are reported relative to deuterated solvents (39.52 ppm for DMSO-d_6). 2D DOSY NMR experiments were performed on a DRX 300 spectrometer with temperature and gradient calibration prior to the measurements, and the temperature was controlled at 298 K during the measurements. Mass analysis were collected on an AccuToF-LC, JMS-T100LP Mass spectrometer (JEOL, Japan) (for ESI) and AccuToF-GCv-4G, JMST100GCV Mass spectrometer (JEOL, Japan) (for FD). CSI-MS measurements were recorded on a UHR-ToF Bruker Daltonik (Bremen, Germany) maXis, an ESI-ToF MS capable of resolution of at least 40.000 FWHM, which was coupled to a Bruker cryospray unit. Detection was in positive-ion mode and the source voltage was between 5 kV and 6 kV. The flow rates were 280 µL/hour. The drying gas (N_2) was held at -35 ℃ and the spray gas was held at -40 ℃. The machine was calibrated prior to every experiment via direct infusion of the Agilent ESI-ToF in the low concentration tuning mixture, which provided an *m/z* range of singly charged peaks up to 2700 Da in both ion modes. NHC-AuCl was purchased from Sigma Aldrich. Compound **A**,[1] allenol[2] and hex-4-ynoic acid[3] were synthesized according to literature procedures. The calculations of cage structures were conducted with Molecular Dynamics (MD) simulations.[4]

S1. Synthesis and characterization of ligands (L^{free} and L^{AuCl})

Synthesis of compound B:

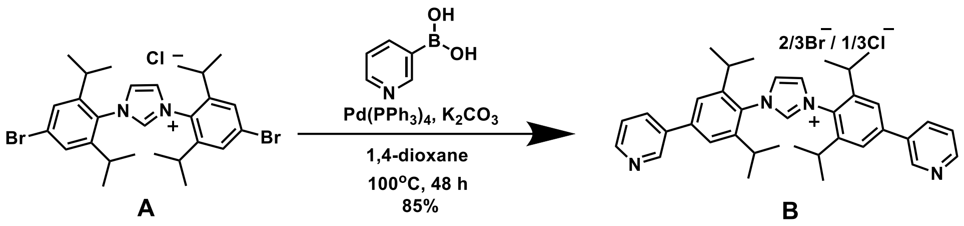

To a solution of **A** (1.00 g, 1.72 mmol), $Pd(PPh_3)_4$ (0.400 g, 0.34 mmol) and 3-Pyridineboronic acid (0.630 g, 5.15 mmol) in degassed 1,4-dioxane (10 mL), 2.6 mL of degassed K_2CO_3 aq. (2M) was added. Then the mixture was stirred at 100 ℃ for 48 h under N_2. After cooling down to room temperature, 10 mL water was added, and the suspention was evaporated under reduced presure. The crude product was purified by chromatography on silica gel (CH_2Cl_2/CH_3OH = 5:1) to give compound **B** as a white powder in a yiled of 85%. ^1H NMR (400 MHz, DMSO-d_6, ppm) δ 10.26 (s, 1H), 9.05 (d, *J* = 2.4 Hz, 2H), 8.67 (dd, *J* = 4.7, 1.5 Hz, 2H), 8.64 (d, *J* = 1.5 Hz, 2H), 8.27 (t, *J* = 2.0 Hz, 1H), 8.24 (t, *J* = 2.0 Hz, 1H), 7.85 (s, 4H), 7.57 (dd, *J* = 7.9, 4.8 Hz, 2H), 2.42 (q, *J* = 6.8 Hz, 4H), 1.36 (d, *J* = 6.8 Hz, 12H), 1.26 (d, *J* = 6.8 Hz, 12H). ^{13}C NMR (100 MHz, DMSO-d_6, ppm) δ 149.30, 148.12, 145.75, 140.76, 139.35, 134.84, 134.62, 129.86, 126.34, 123.91, 123.32, 28.96, 24.00, 23.00. HRMS (ESI^+) calc. for $(C_{37}H_{43}N_4)^+$ 543.3488, found as 543.3458.

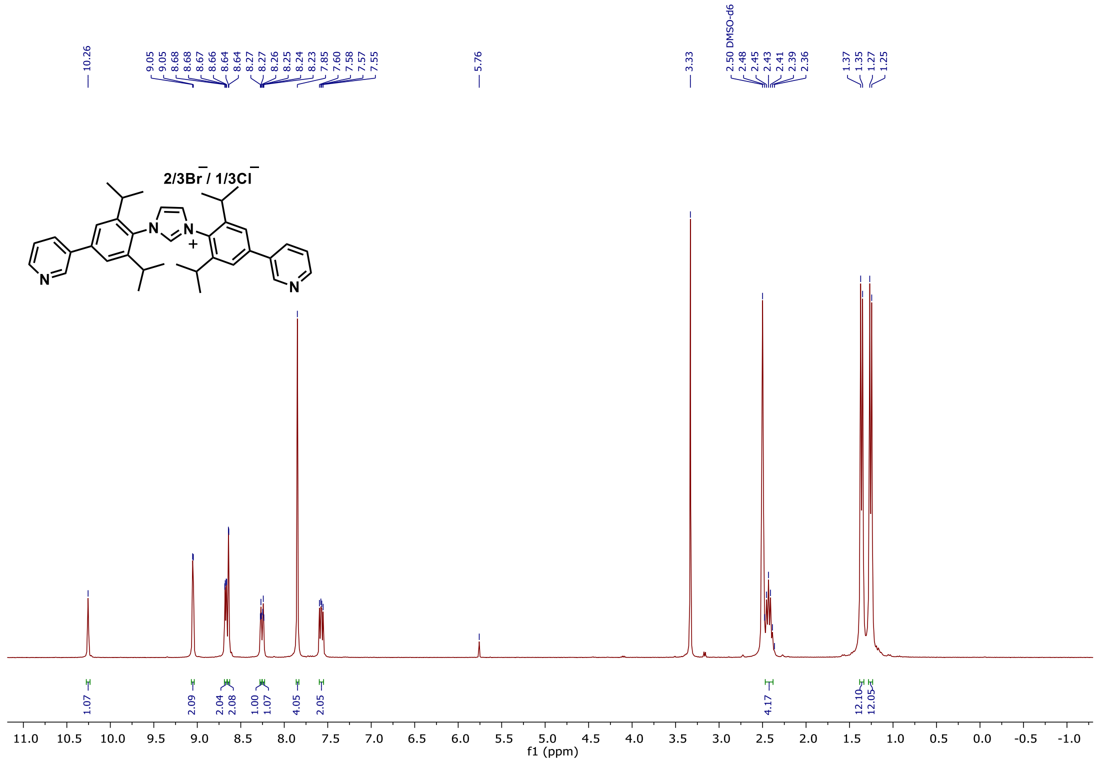

Figure S1. ^{1}H NMR spectrum (400 MHz, 298 K) of compound **B** in DMSO-d_6.

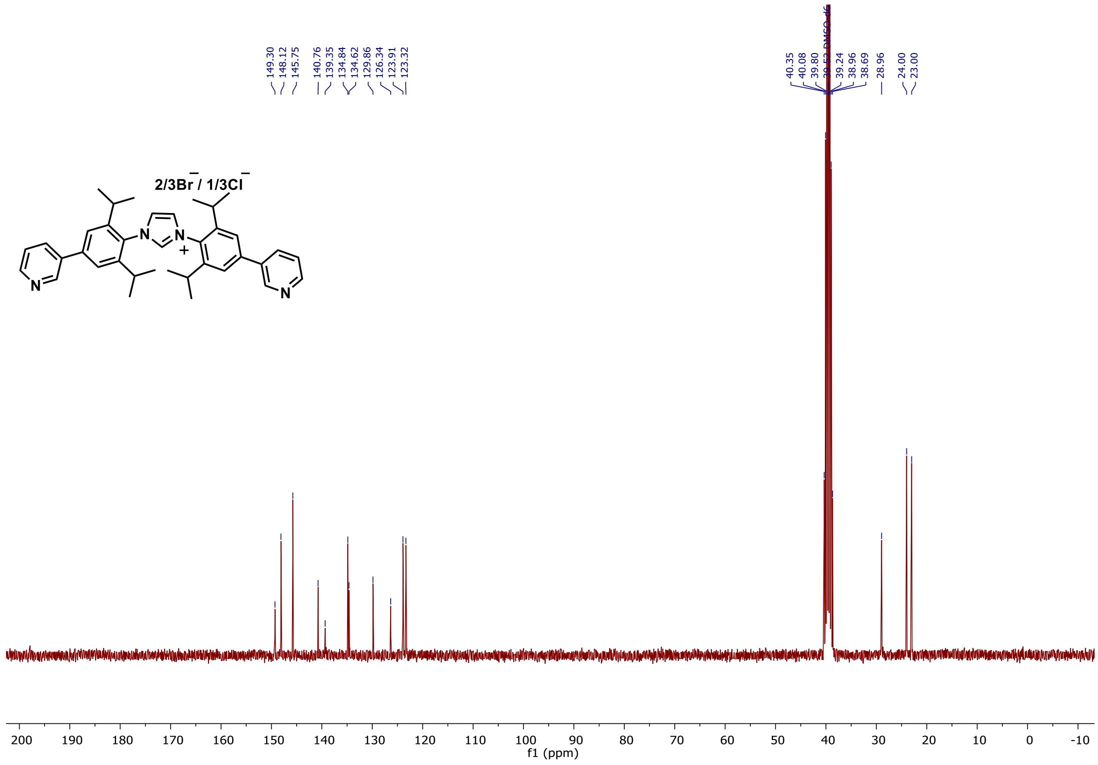

Figure S2. ^{13}C NMR spectrum (100 MHz, 298 K) of compound **B** in DMSO-d_6.

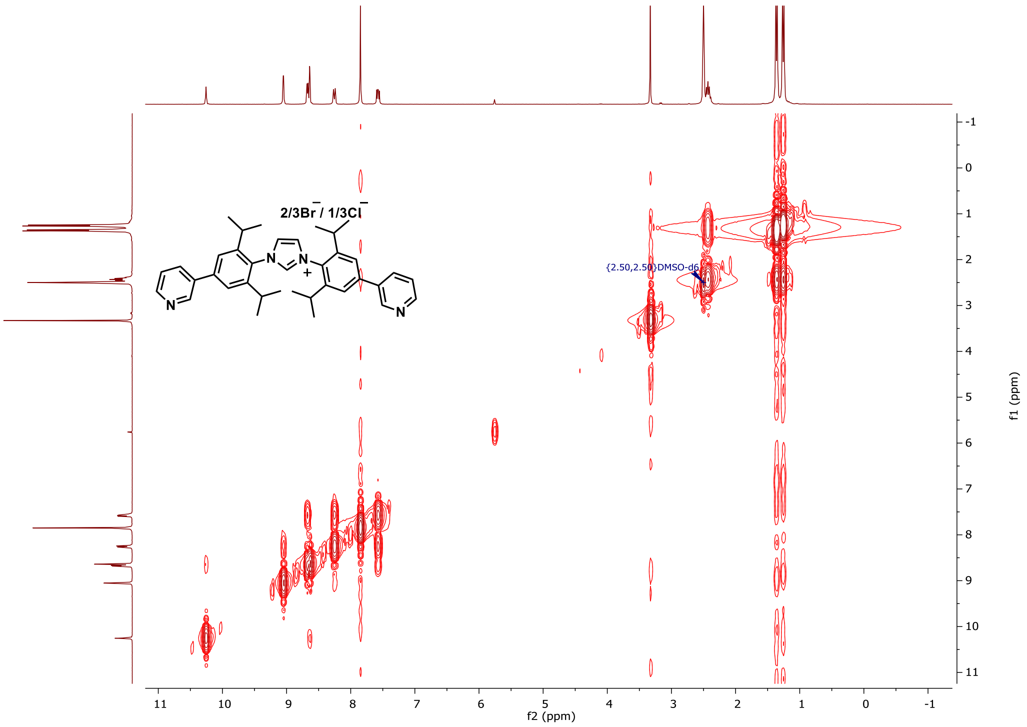

Figure S3. ^{1}H-^{1}H COSY spectrum (400 MHz, 298 K) of compound **B** in DMSO-d_6.

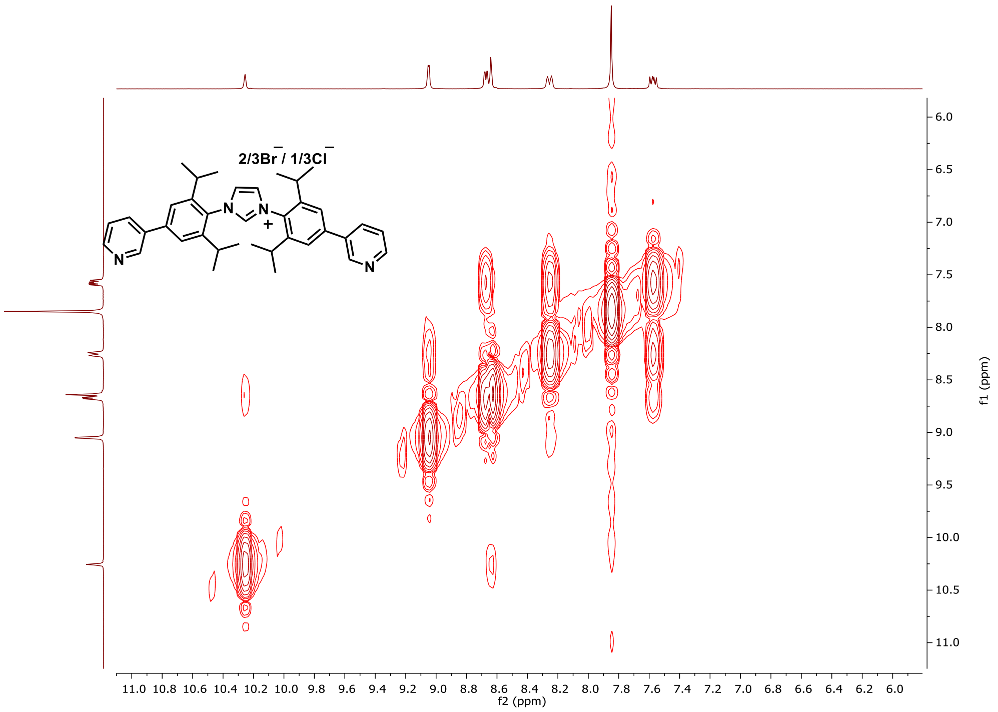

Figure S4. Aromatic area of ^{1}H-^{1}H COSY spectrum (400 MHz, 298 K) of compound **B** in DMSO-d_6.

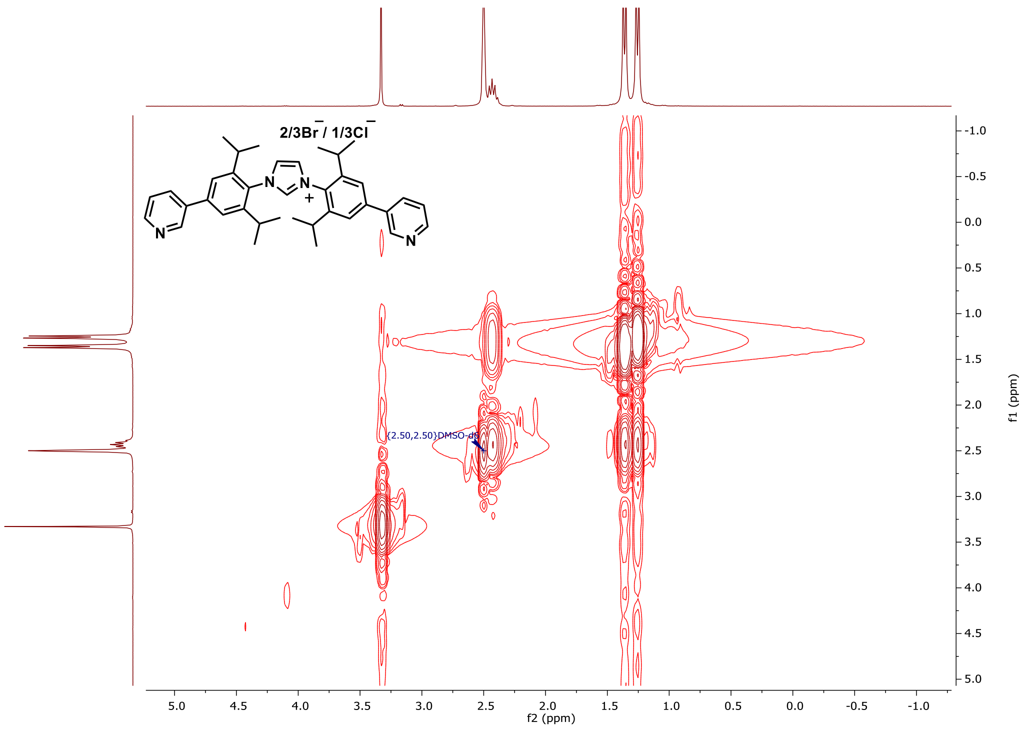

Figure S5. Aliphatic area of ^1H-^1H COSY spectrum (400 MHz, 298 K) of compound **B** in DMSO-d_6.

Synthesis of free ligand (L^{free}):

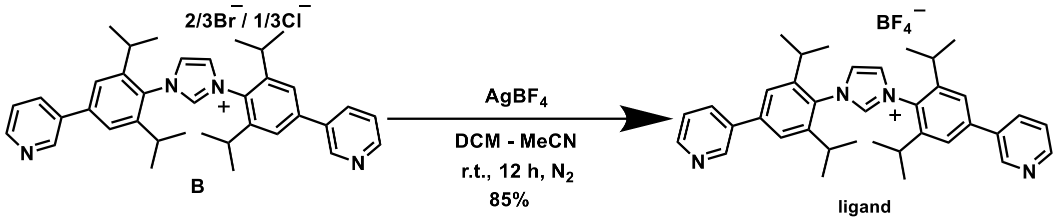

The free ligand (**L^{free}**) was synthesized by exchanging the counter ion with silver salt. In a dried schlenk flask, the suspension of compound **B** (0.100 g, 0.16 mmol) and $AgBF_4$ (0.320 mg, 0.16 mmol) in a mixed solvent of CH_2Cl_2 and CH_3CN (1:1, *v/v*, 4 mL) was stirred at room temperature under N_2. After 12 h, the reaction mixture was filtered, and the filtrate was dropwised into 50 mL diethyl ether. The precipitation was collected and dried under vacuum to give the desired product **L^{free}** as a white powder with a yield of 85%. ^{1}H NMR (300 MHz, DMSO-d_6, ppm) δ 10.22 (s, 1H), 9.05 (s, 2H), 8.67 (d, J = 4.5 Hz, 2H), 8.62 (s, 2H), 8.25 (d, J = 8.0 Hz, 2H), 7.85 (s, 4H), 7.57 (dd, J = 7.8, 4.8 Hz, 2H), 2.42 (dt, J = 13.4, 6.6 Hz, 4H), 1.36 (d, J = 6.7 Hz, 12H), 1.25 (d, J = 6.8 Hz, 12H). ^{13}C NMR (75 MHz, DMSO-d_6, ppm) δ 149.35, 148.16, 145.79, 140.80, 139.37, 134.90, 134.66, 129.88, 126.34, 123.97, 123.38, 28.99, 24.04, 23.02. HRMS (ESI$^+$) calc. for $(C_{37}H_{43}N_4)^+$ 543.3488, found as 543.3471.

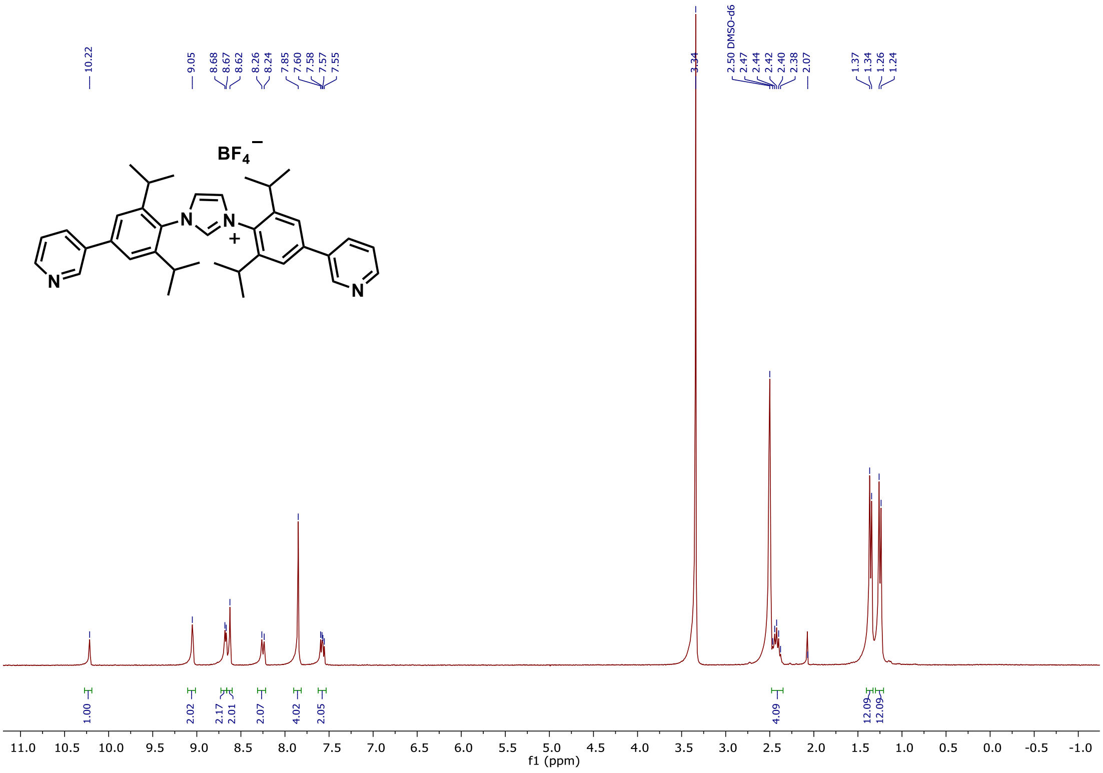

Figure S6. ^{1}H NMR spectrum (300 MHz, 298 K) of $\mathbf{L}^{\mathbf{free}}$ in DMSO-d_6.

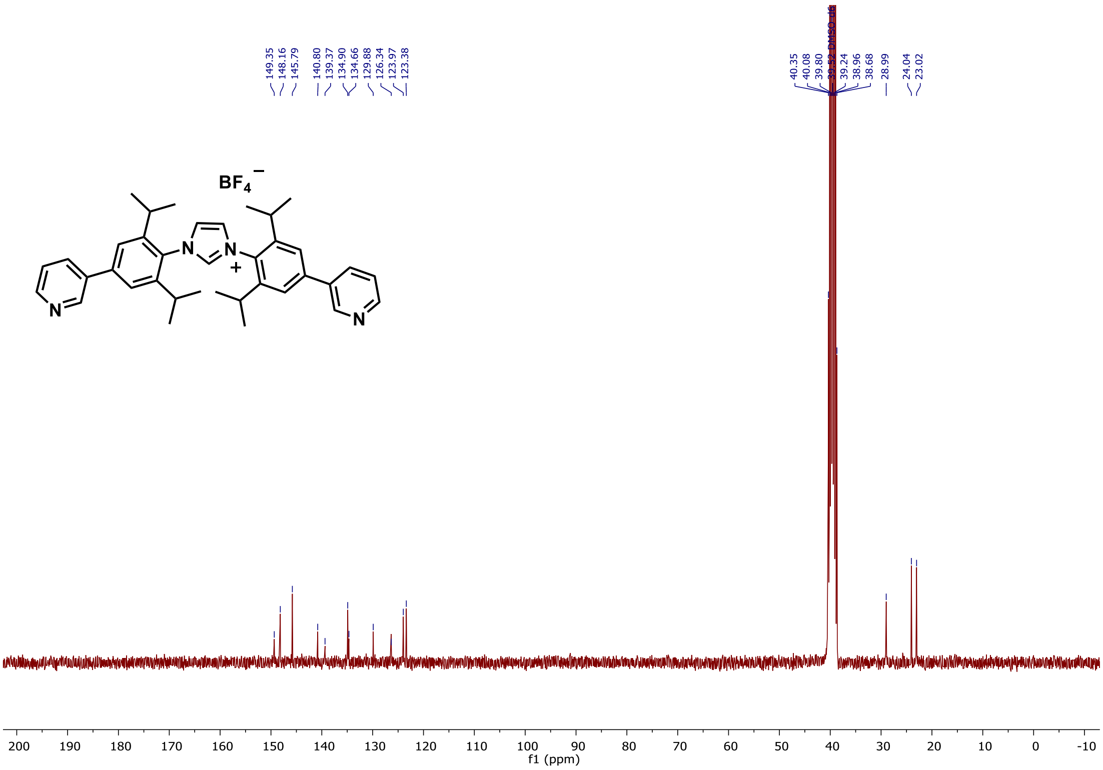

Figure S7. ^{13}C NMR spectrum (75 MHz, 298 K) of $\mathbf{L}^{\mathbf{free}}$ in DMSO-d_6.

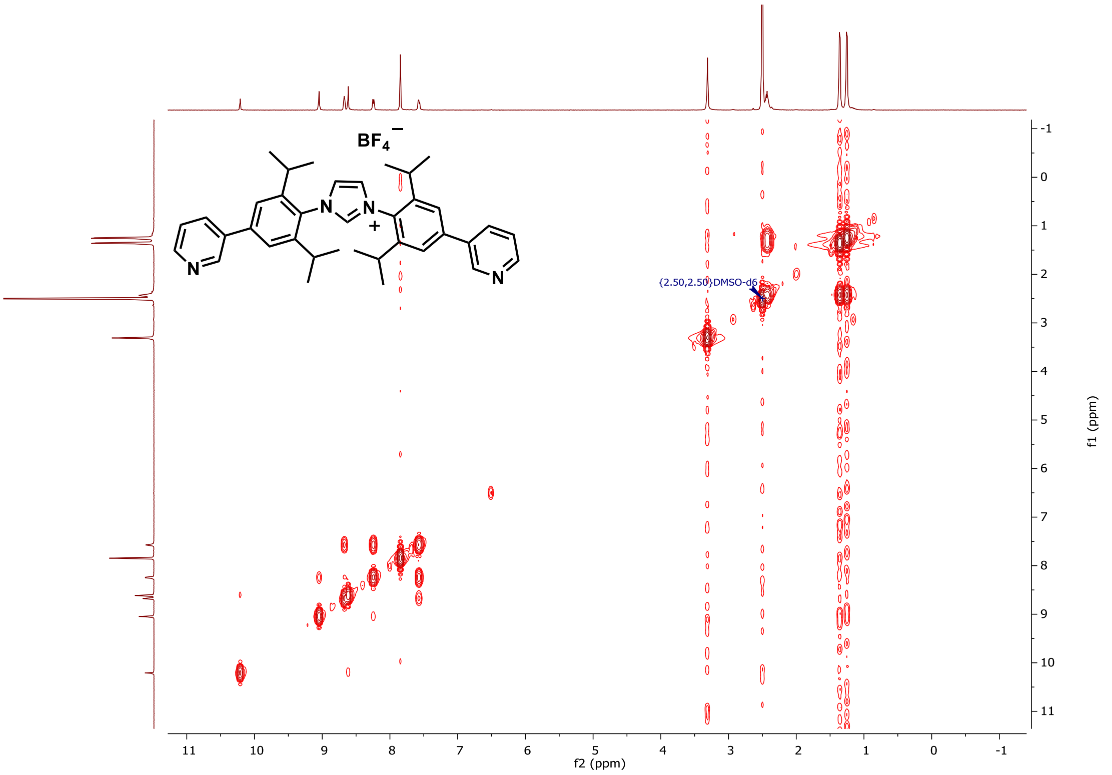

Figure S8. ^{1}H-^{1}H COSY spectrum (400 MHz, 298 K) of $\mathbf{L}^{\mathbf{free}}$ in DMSO-d_6.

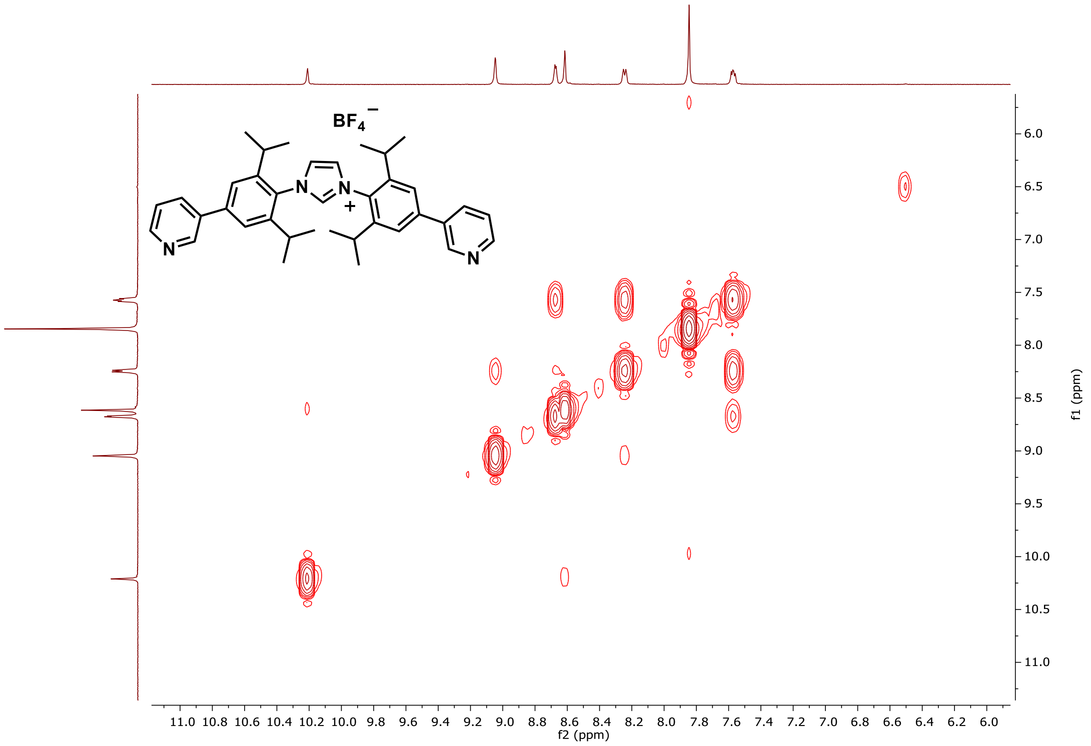

Figure S9. Aromatic area of ^{1}H-^{1}H COSY spectrum (400 MHz, 298 K) of $\mathbf{L}^{\mathbf{free}}$ in DMSO-d_6.

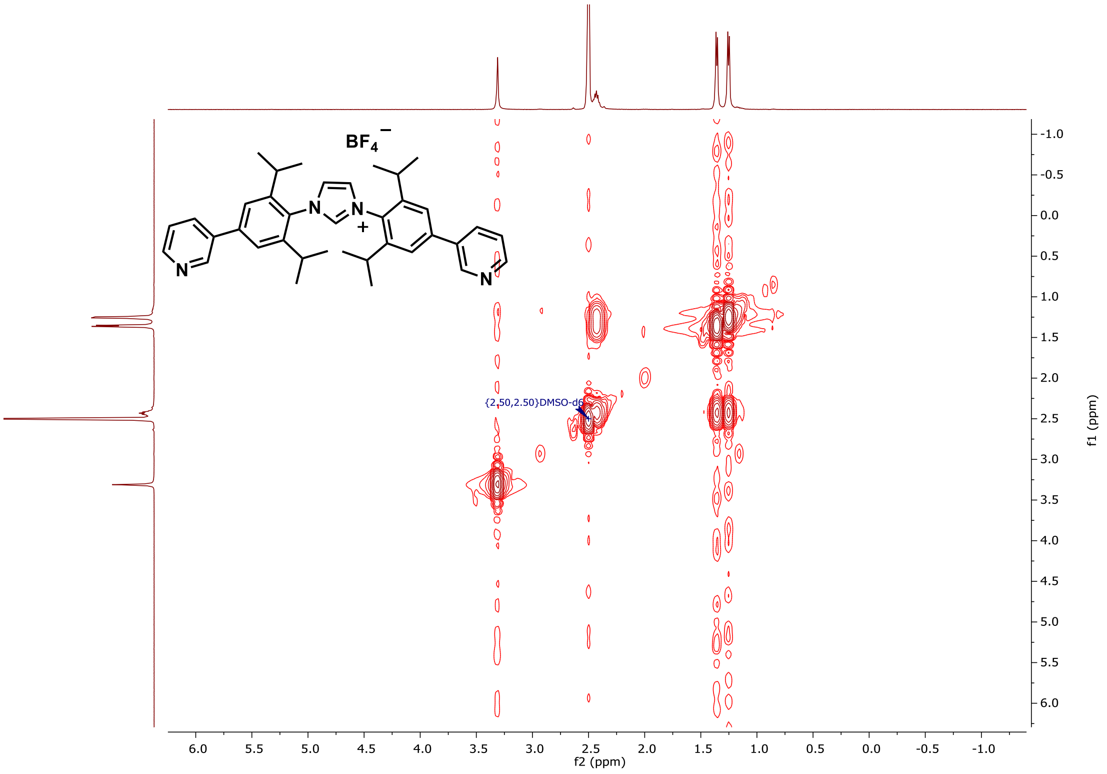

Figure S10. Aliphatic area of ^{1}H-^{1}H COSY spectrum (400 MHz, 298 K) of **L^{free}** in DMSO-d_6.

Synthesis of Au ligand :

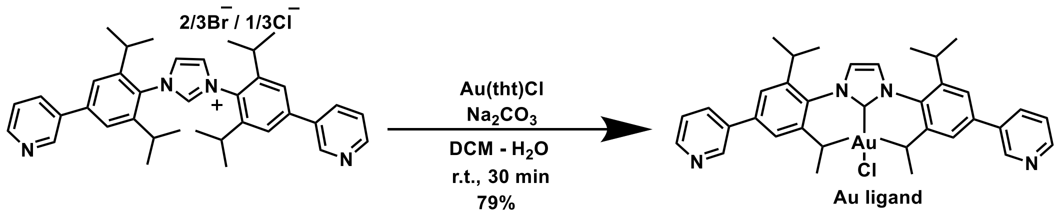

In a flask, compound **B** (0.050 g, 0.08 mmol) and Au(tht)Cl (0.026 g, 0.08 mmol) was dissolved in 7.2 mL CH_2Cl_2, then 3.52 mL of Na_2CO_3 aq. (0.130 g, 1.23 mmol) was added. The mixture was stirred vigorously for 30 min at room temperature. The mixture was fitrated, and the filtrate was precipited in diethyl ether. Then the solid was collected and dried under reduced pressure to give $\mathbf{L}^{\mathbf{AuCl}}$ as a white powder in a yiled of 79%. ^{1}H NMR (300 MHz, DMSO-d_6, ppm) δ 9.04 (s, 2H), 8.64 (d, *J* = 4.0 Hz, 2H), 8.25 (d, *J* = 7.8 Hz, 2H), 8.08 (s, 2H), 7.75 (s, 4H), 7.54 (dd, *J* = 7.6, 4.9 Hz, 2H), 2.64 – 2.53 (m, 4H), 1.33 (dd, *J* = 16.0, 6.7 Hz, 24H). ^{13}C NMR (75 MHz, DMSO-d_6, ppm) δ 176.06, 148.96, 148.07, 146.28, 139.24, 134.74, 124.70, 123.89, 122.78, 38.69, 28.68, 23.95, 23.41. HRMS (ESI$^+$) calc. for $(C_{37}H_{43}AuN_4 + K)^+$ 780.3322, found as 780.3340.

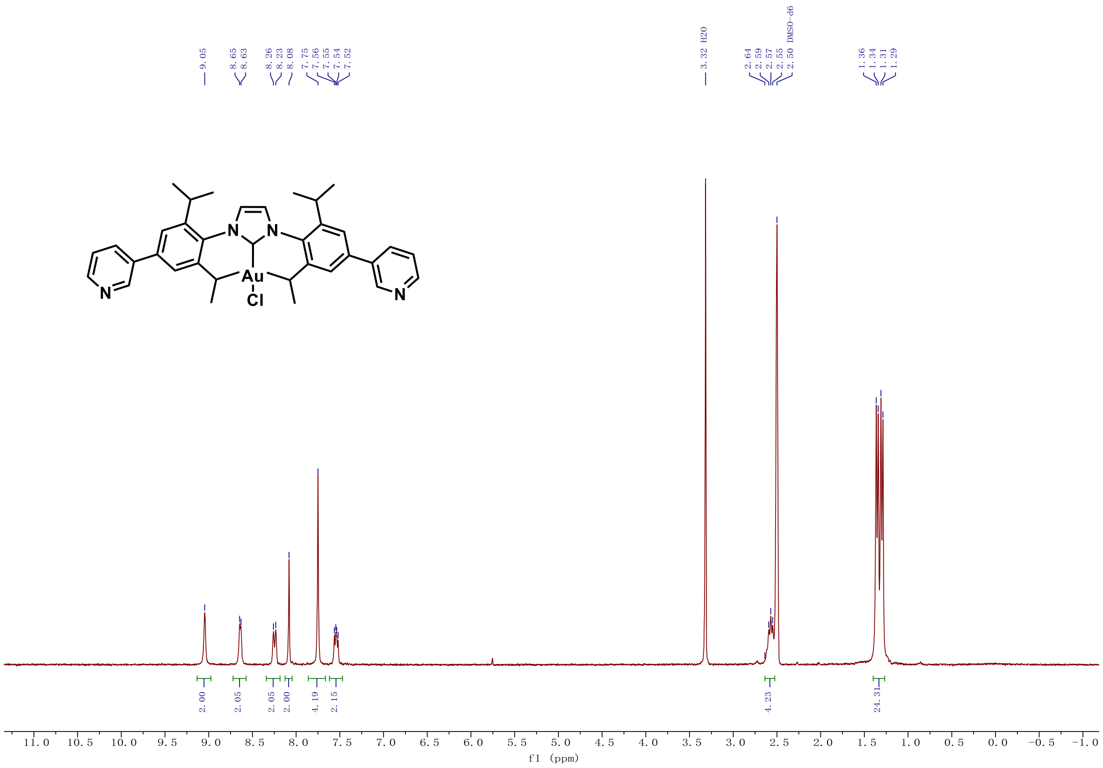

Figure S11. ^{1}H NMR spectrum (300 MHz, 298 K) of $\mathbf{L^{AuCl}}$ in DMSO-d_6.

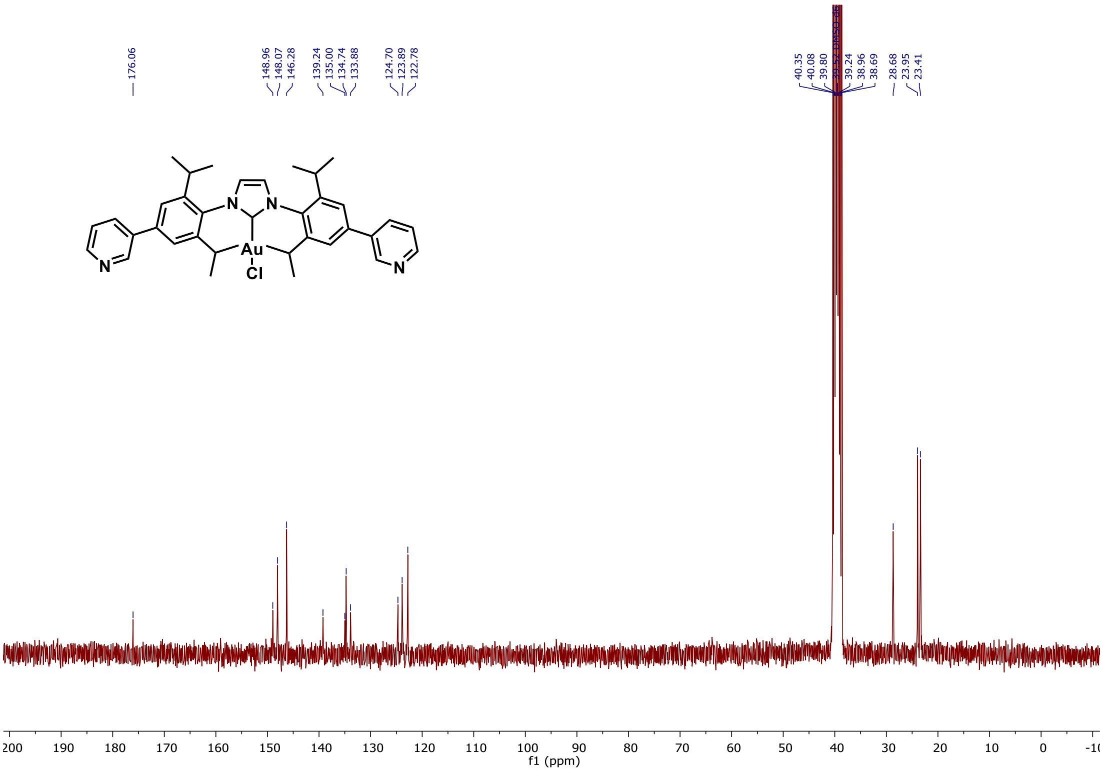

Figure S12. ^{13}C NMR spectrum (75 MHz, 298 K) of $\mathbf{L^{AuCl}}$ in DMSO-d_6.

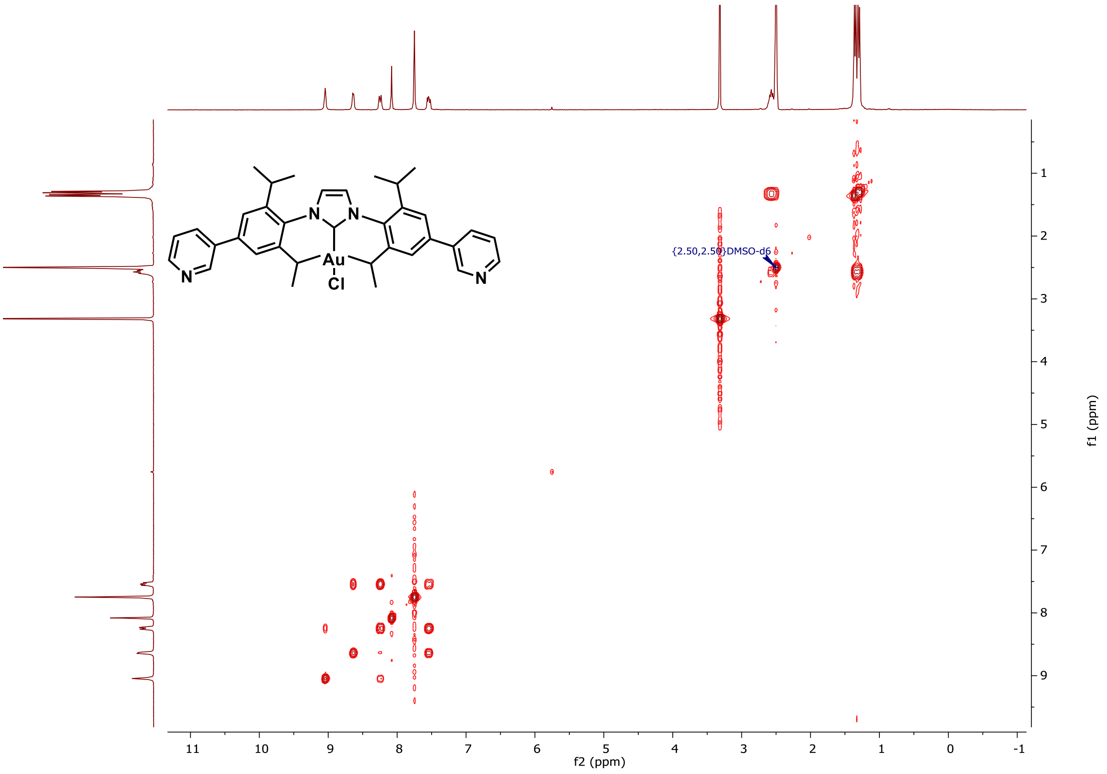

Figure S13. ^{1}H-^{1}H COSY spectrum (300 MHz, 298 K) of **L**$^{\mathbf{AuCl}}$ in DMSO-d_6.

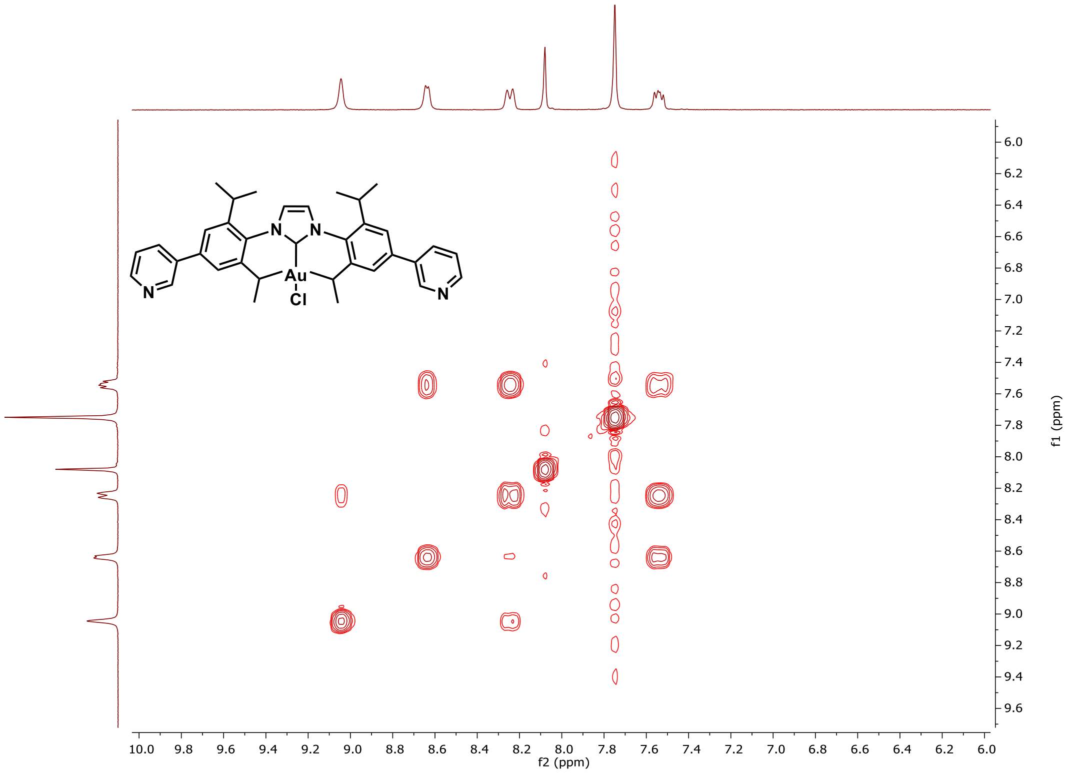

Figure S14. Aromatic area of ^{1}H-^{1}H COSY spectrum (300 MHz, 298 K) of **L**$^{\mathbf{AuCl}}$ in DMSO-d_6.

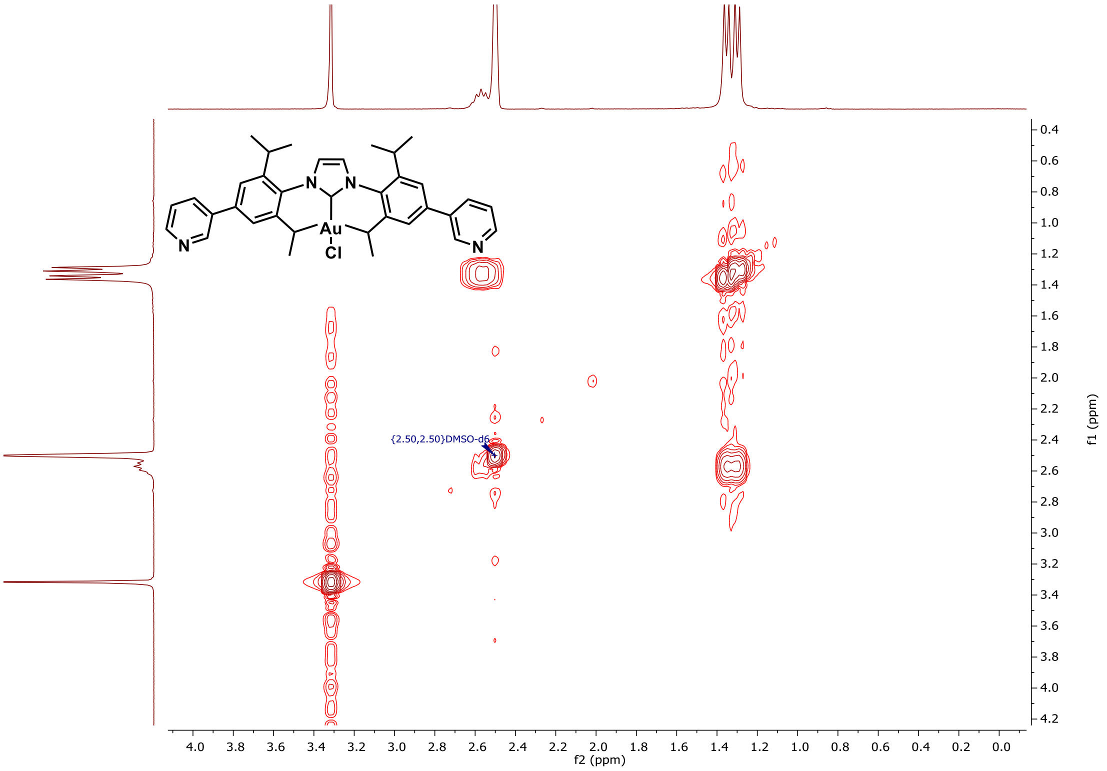

Figure S15. Aliphatic area of ^{1}H-^{1}H COSY spectrum (300 MHz, 298 K) of $\mathbf{L}^{\mathbf{AuCl}}$ in DMSO-d_6.

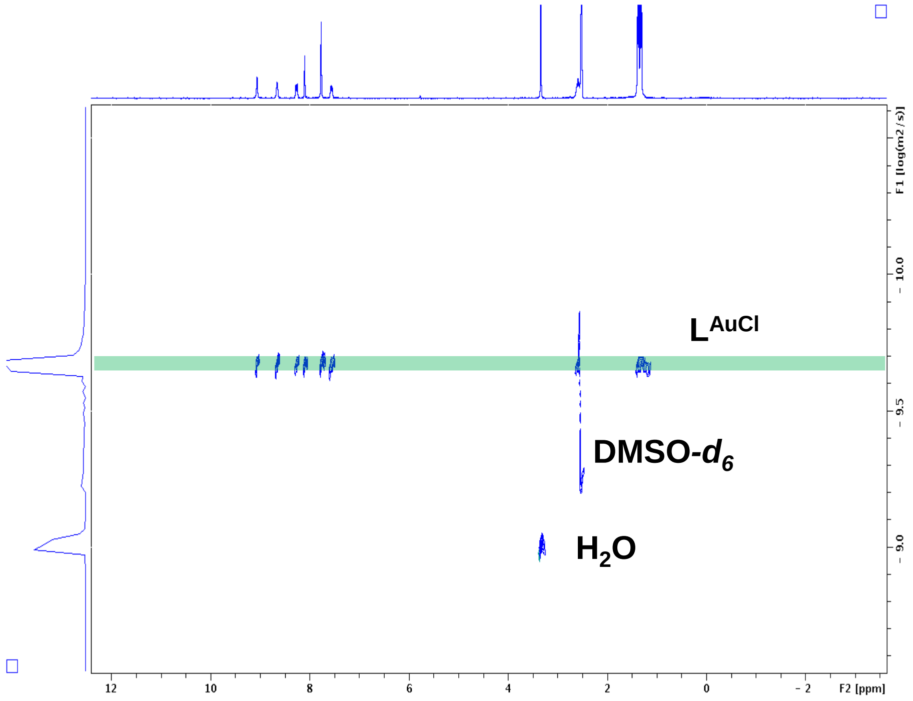

Figure S16. ^{1}H DOSY spectrum (300 MHz, 298 K) of $\mathbf{L}^{\mathbf{AuCl}}$ in DMSO-d_6 (log D = -9.70, R ≈ 1.12 nm).

S2. Synthesis and characterization of $Pd_3L^{free}_6$ and $Pd_6L^{AuCl}_{12}$

Synthesis of $Pd_3L^{free}_6$:

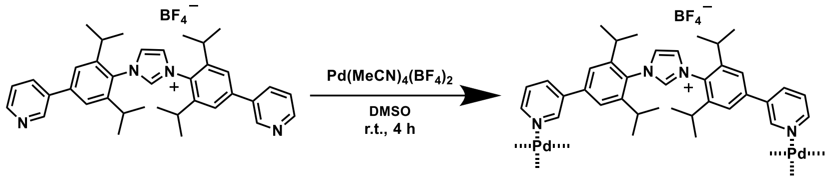

A solution containing $Pd(MeCN)_4(BF_4)_2$ (4.442 mg, 0.01 mmol) and ligand ($\mathbf{L}^{free}$) (12.610 mg, 0.02 mmol) in 2.0 mL DMSO-d_6 was vigorously stirred at room temperature for 4 h to give a yellow solution. The empty cage ($Pd_3\mathbf{L}^{free}_6$) was then collected by precipitation in excess amount of diethyl ether, dried over vacuum and obtained as an air stable light-yellow solid (86%). ^{1}H NMR (300 MHz, DMSO-d_6, ppm) δ 9.97 (s, 1H), 9.75 (s, 4H), 8.66 (d, J = 8.0 Hz, 2H), 8.59 (s, 2H), 8.15 (s, 2H), 8.00 (s, 2H), 7.80 (s, 2H), 7.80 (s, 2H), 2.24 (s, 4H), 1.49 (s, 6H), 1.31 (s, 12H), 1.06 (s, 6H). ^{13}C NMR (126 MHz, DMSO-d_6, ppm) δ 150.57, 148.82, 146.68, 146.00, 137.51, 131.07, 127.55, 123.74, 123.34, 118.06, 39.02, 29.23, 29.00, 24.42, 23.60, 23.11, 22.79, 22.50. ESI-MS (m/z): 1454.2785 (calc. for $[Pd_3(\mathbf{L}^{free})_6\ 9BF_4]^{3+}$ 1454.2812), 1068.9581 (calc. for $[Pd_3(\mathbf{L}^{free})_6\ 8BF_4]^{4+}$ 1068.9600), 837.7661 (calc. for $[Pd_3(\mathbf{L}^{free})_6\ 7BF_4]^{5+}$ 837.7672), 683.6382 (calc. for $[Pd_3(\mathbf{L}^{free})_6\ 6BF_4]^{6+}$ 683.6384), 573.6895 (calc. for $[Pd_3(\mathbf{L}^{free})_6\ 5BF_4]^{7+}$ 573.6897), 491.1032 (calc. for $[Pd_3(\mathbf{L}^{free})_6\ 4BF_4]^{8+}$ 491.1029), 426.8690 (calc. for $[Pd_3(\mathbf{L}^{free})_6\ 3BF_4]^{9+}$ 426.8688). DOSY NMR (DMSO-d_6, 298 K, log D = -10.078, R ≈ 2.61 nm).

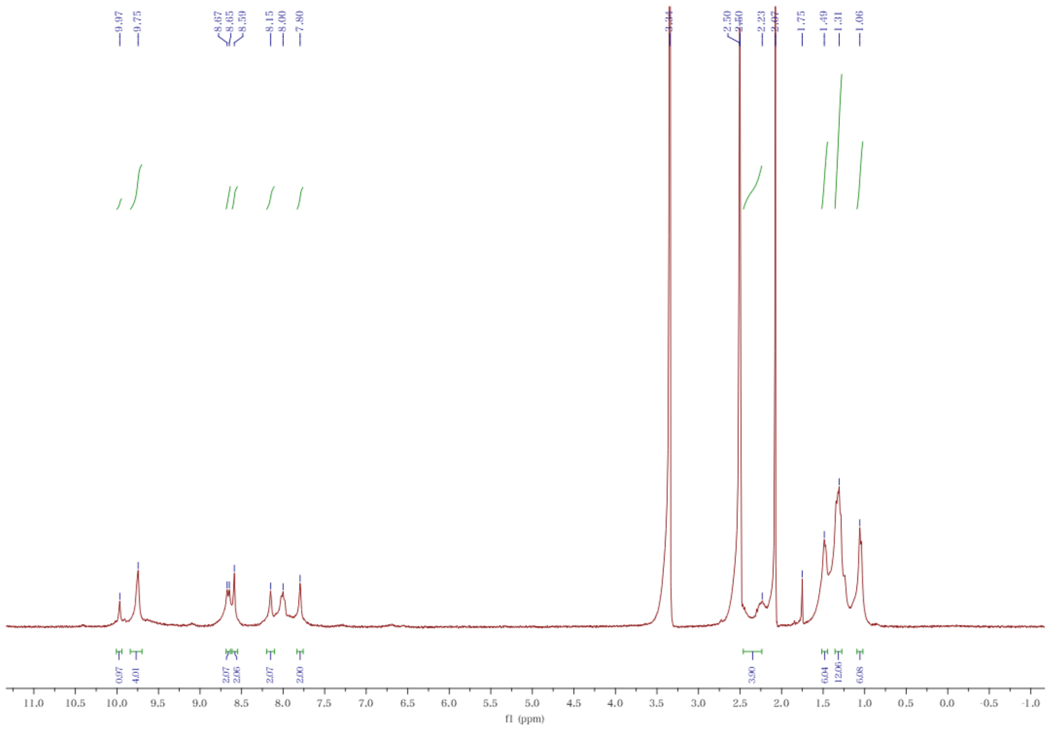

Figure S17. ^{1}H NMR spectrum (300 MHz, 298 K) of $Pd_3\mathbf{L}^{\mathbf{free}}_6$ in DMSO-d_6.

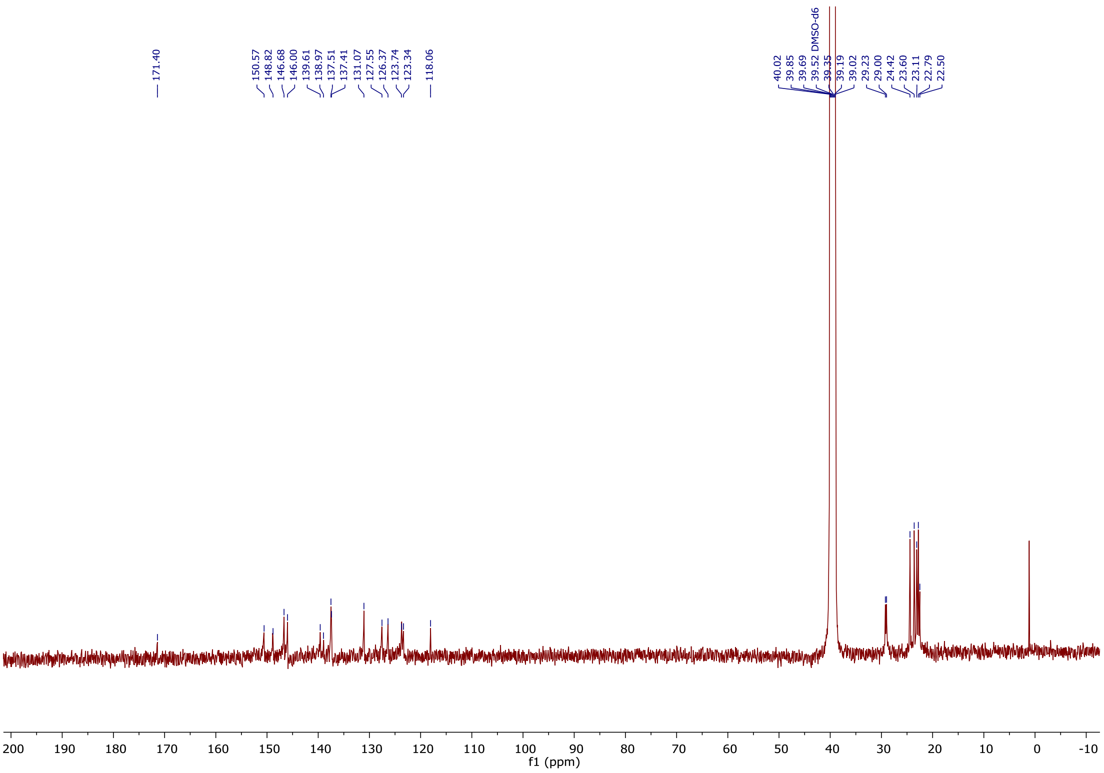

Figure S18. ^{13}C NMR spectrum (126 MHz, 298 K) of $Pd_3\mathbf{L}^{\mathbf{free}}_6$ in DMSO-d_6.

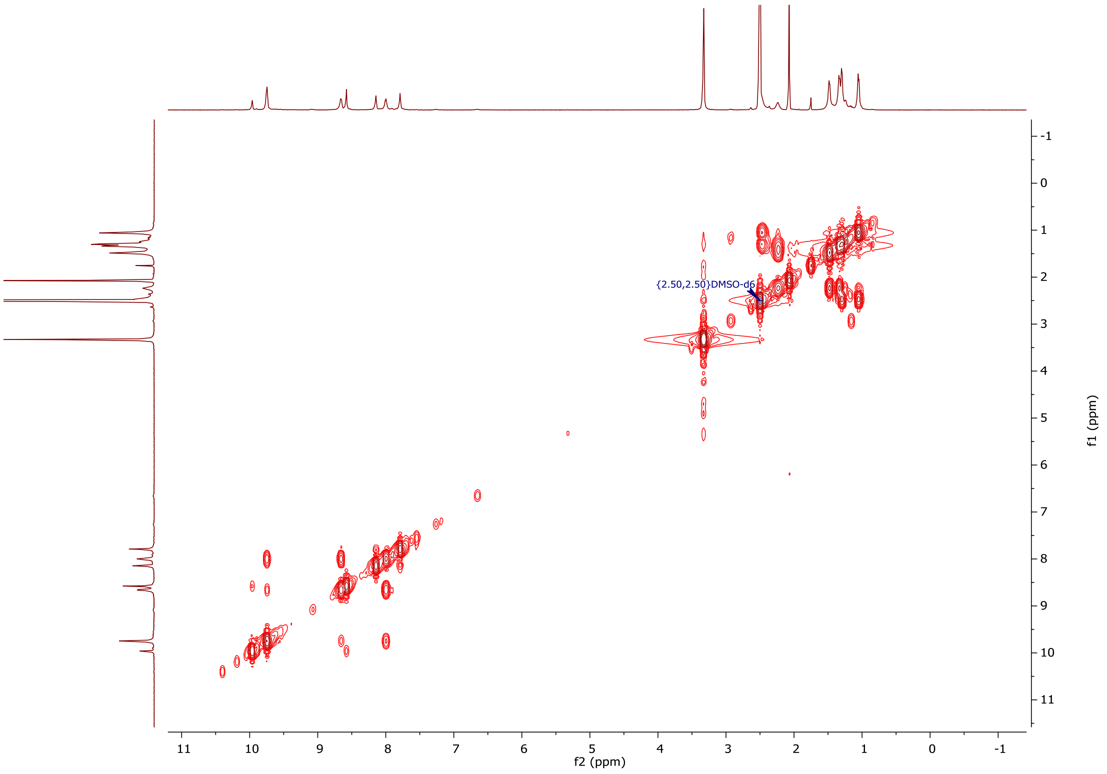

Figure S19. ^{1}H-^{1}H COSY spectrum (400 MHz, 298 K) of Pd_3**L**$^{\mathbf{free}}{}_6$ in DMSO-d_6.

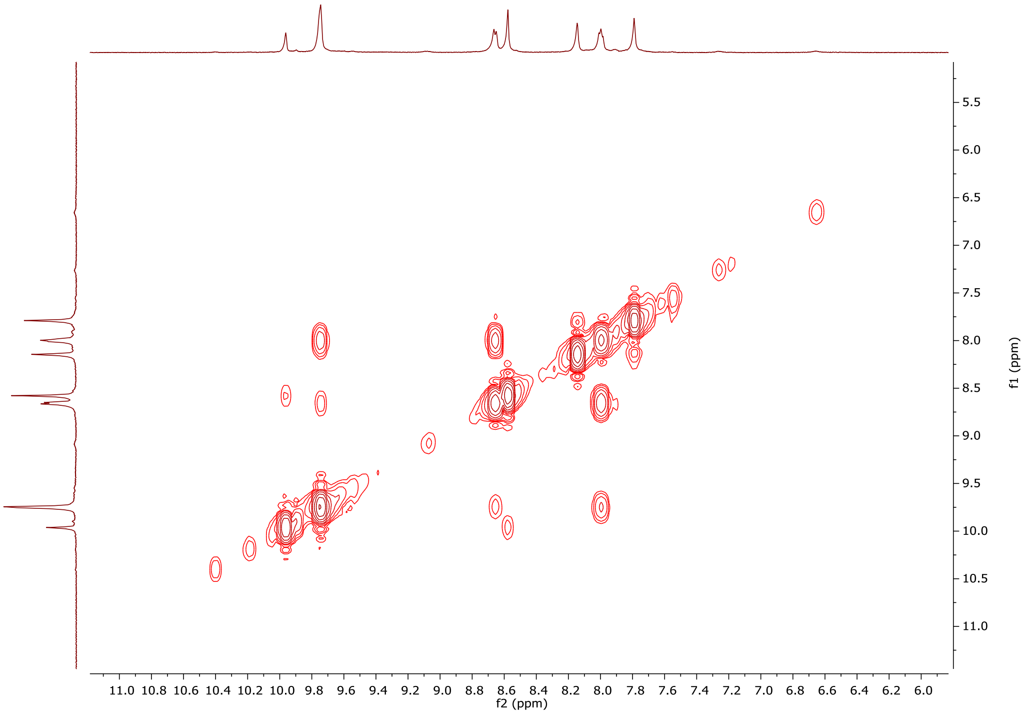

Figure S20. Aromatic area of ^{1}H-^{1}H COSY spectrum (400 MHz, 298 K) of Pd_3**L**$^{\mathbf{free}}{}_6$ in DMSO-d_6.

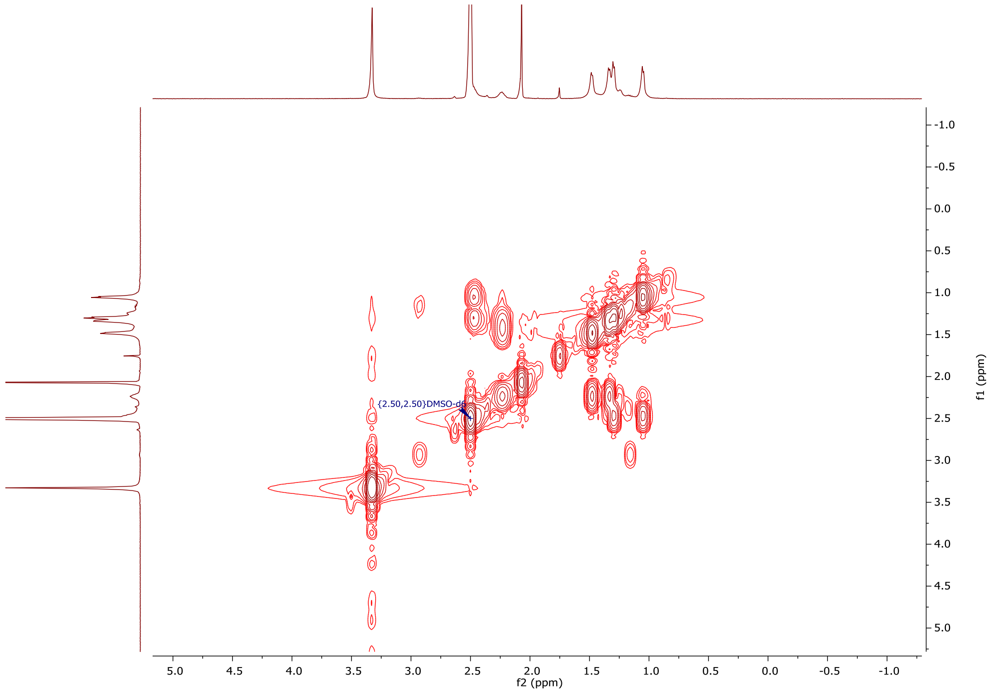

Figure S21. Aliphatic area of ^{1}H-^{1}H COSY spectrum (400 MHz, 298 K) of Pd$_3$**L**$^{\mathbf{free}}{}_6$ in DMSO-*d*$_6$.

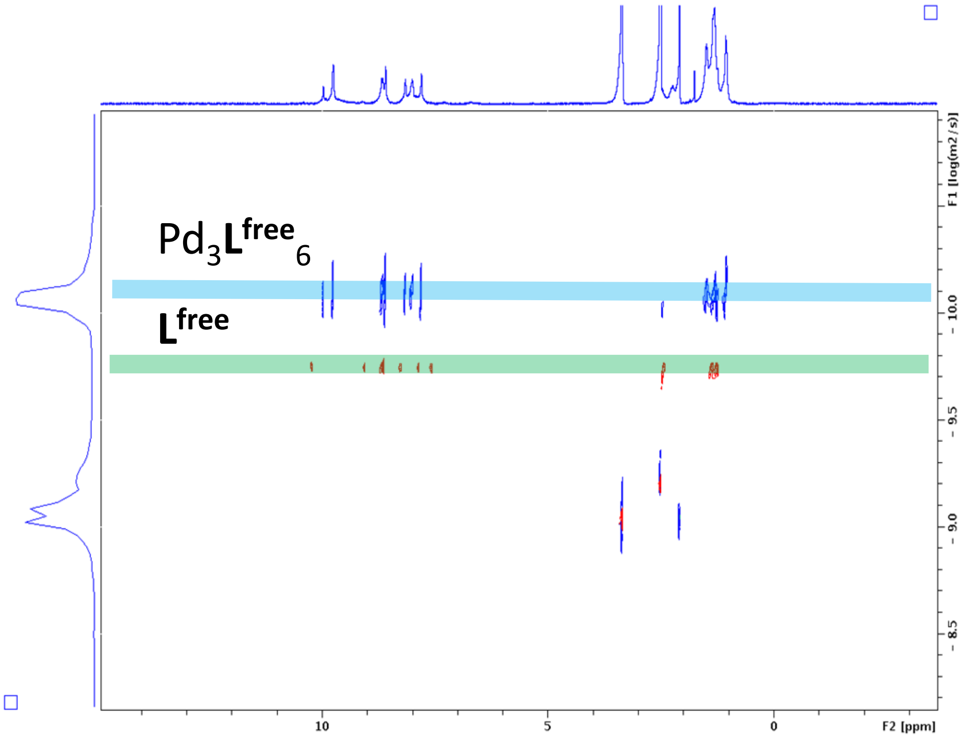

Figure S22. Overlapped ^{1}H DOSY spectrum (300 MHz, 298 K) of Pd$_3$**L**$^{\mathbf{free}}{}_{\mathbf{6}}$ and **L**$^{\mathbf{free}}$ in DMSO-*d*$_6$.

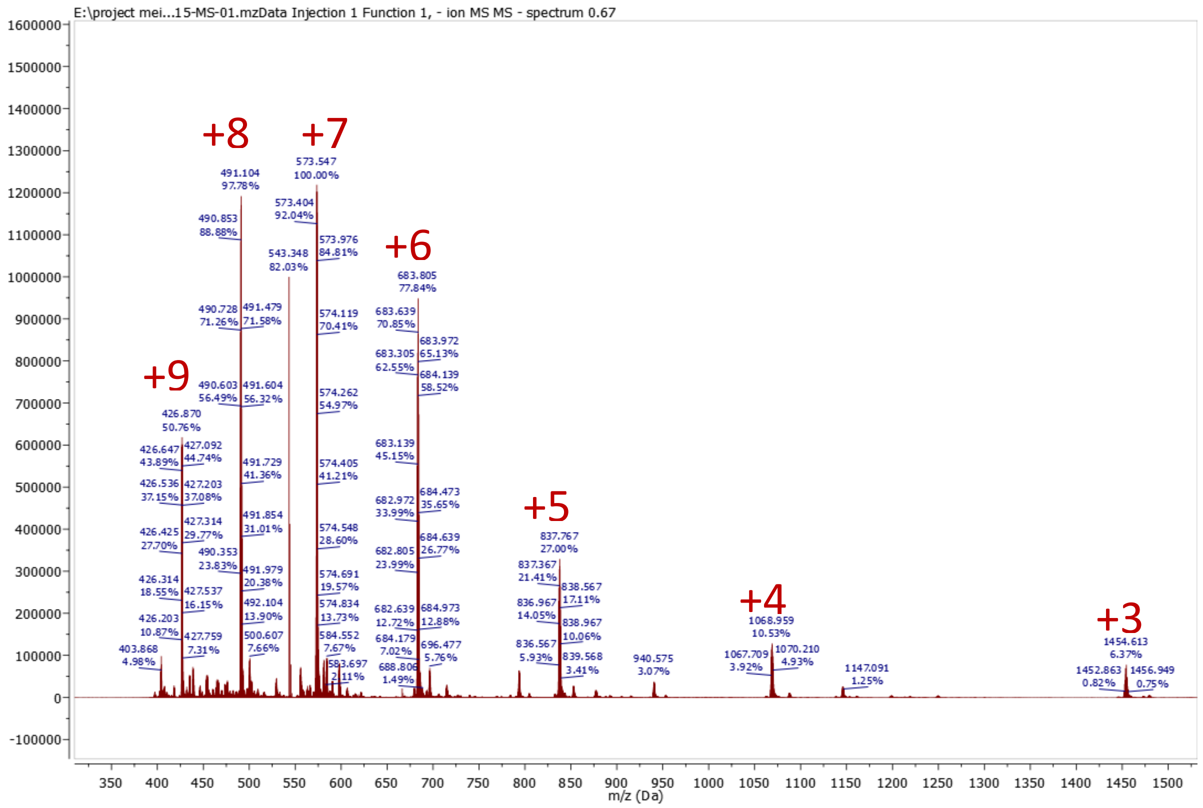

Figure S23. Mass spectrum of $Pd_3\mathbf{L}^{\mathbf{free}}_{\mathbf{6}}$ with minimal amount of $Pd_4\mathbf{L}^{\mathbf{free}}_{8}$.

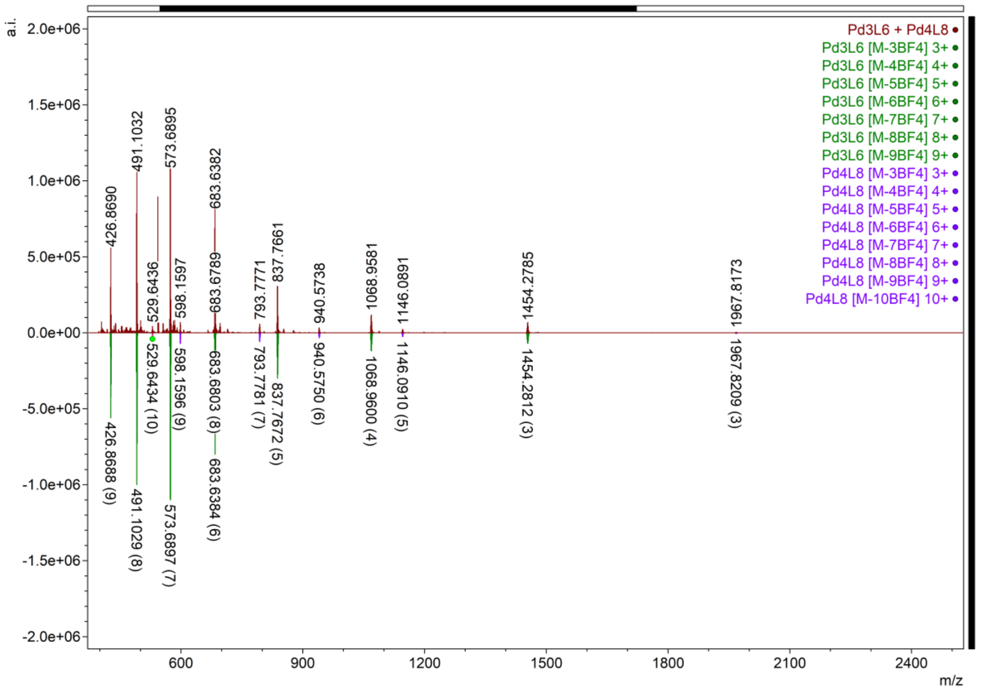

Figure S24. Full mass spectrum of $Pd_3\mathbf{L}^{\mathbf{free}}_{\mathbf{6}}$ with minimal amount of $Pd_4\mathbf{L}^{\mathbf{free}}_{\mathbf{8}}$ in CH_3CN.

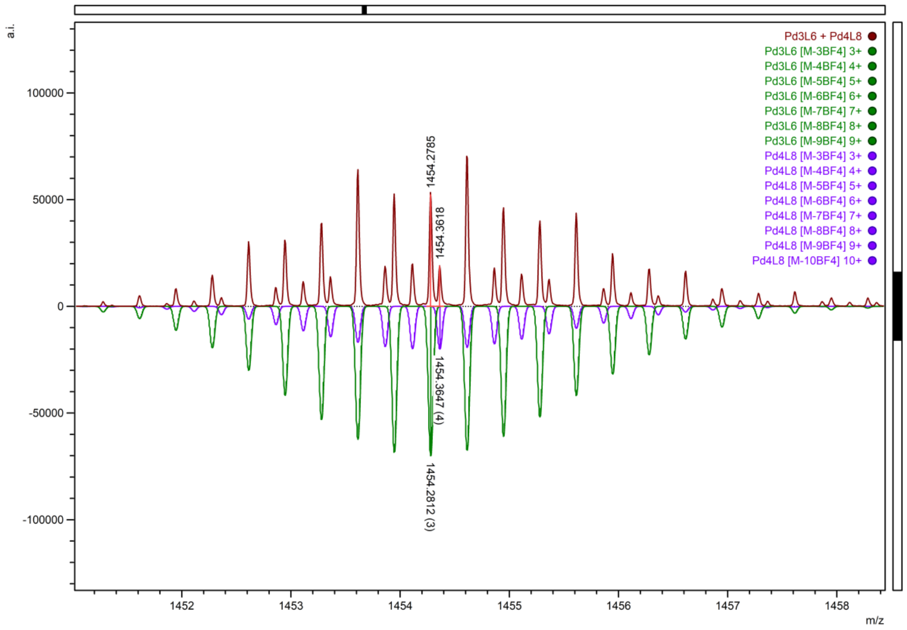

Figure S25. Observed charged species $Pd_3\mathbf{L}^{\mathbf{free}}_6[M\text{-}3BF_4^-]^{3+}$ (red), $Pd_4\mathbf{L}^{\mathbf{free}}_8[M\text{-}4BF_4^-]^{4+}$ (red) and their coorsponding simulated spectra ($Pd_3\mathbf{L}^{\mathbf{free}}_6[M\text{-}3BF_4^-]^{3+}$ (green), $Pd_4\mathbf{L}^{\mathbf{free}}_8[M\text{-}4BF_4^-]^{4+}$ (purple)) in CH_3CN.

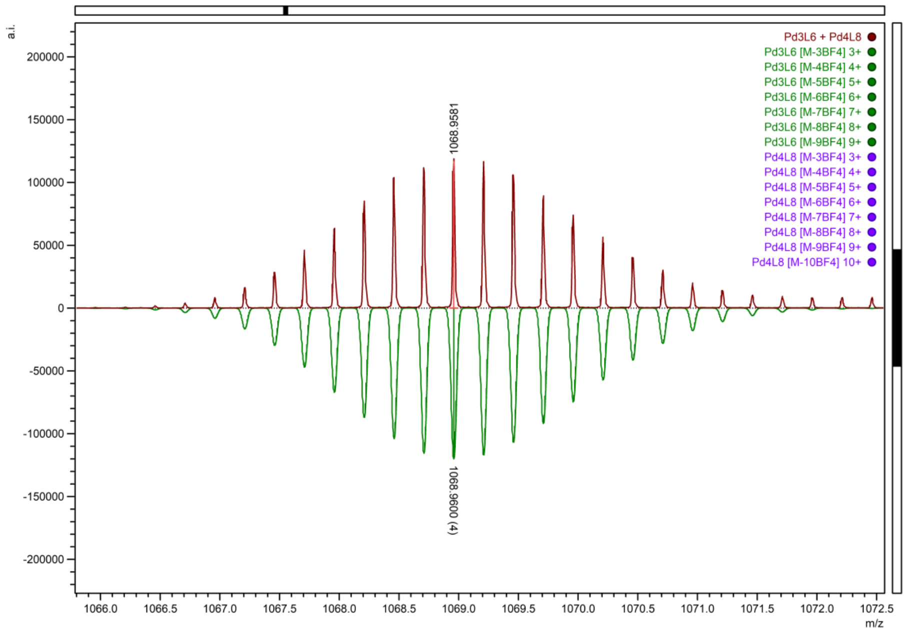

Figure S26. Observed charged species $Pd_3\mathbf{L}^{\mathbf{free}}_6$ $[M\text{-}4BF_4^-]^{4+}$ (red) and simulated isotopic distribution (green) in CH_3CN.

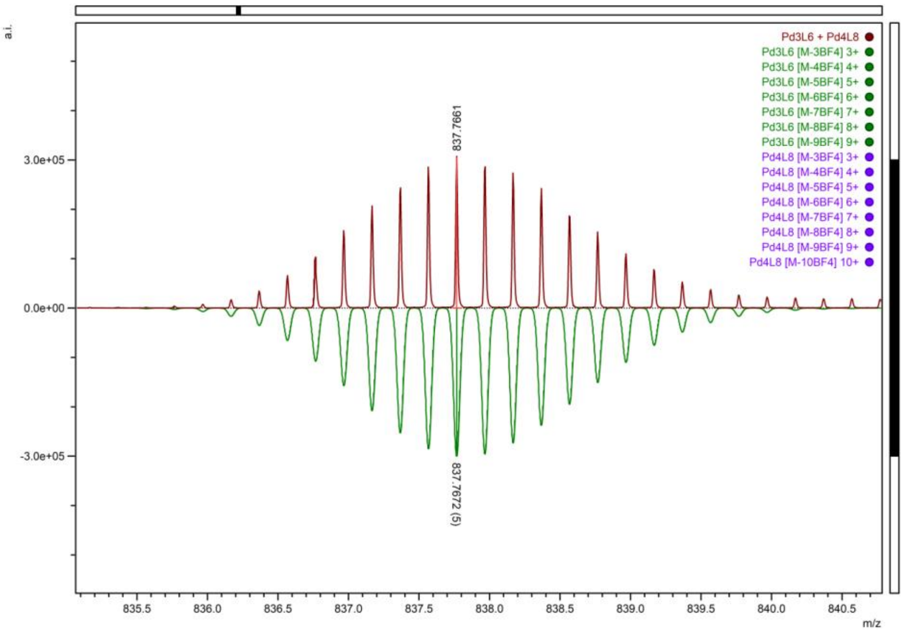

Figure S27. Observed charged species $Pd_3\mathbf{L}^{\mathbf{free}}_6$ $[M\text{-}5BF_4^-]^{5+}$ (red) and simulated isotopic distribution (green) in CH_3CN.

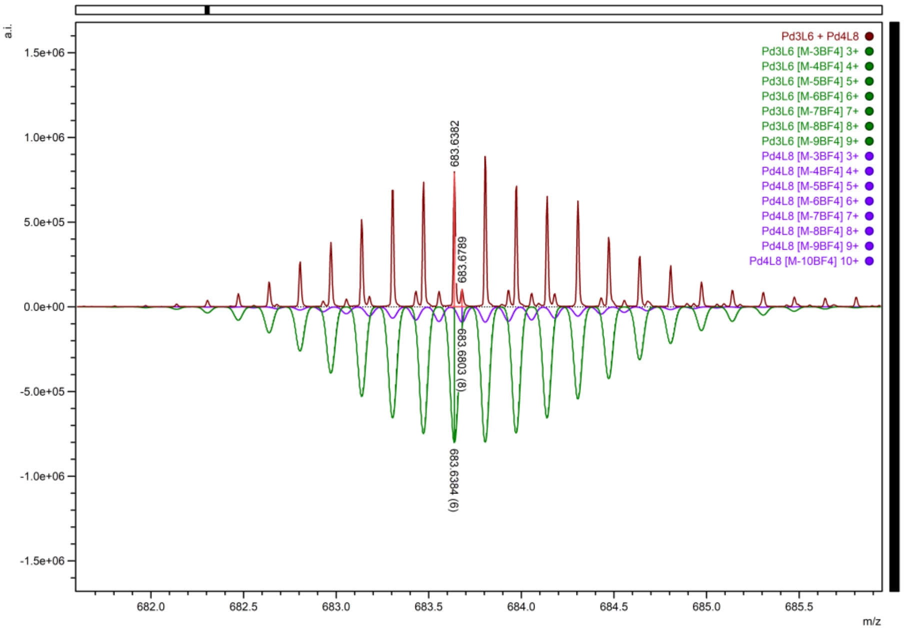

Figure S28. Observed charged species $Pd_3\mathbf{L}^{\mathbf{free}}_6[M\text{-}6BF_4^-]^{6+}$ (red), $Pd_4\mathbf{L}^{\mathbf{free}}_8[M\text{-}8BF_4^-]^{8+}$ (red) and their coorsponding simulated spectrum ($Pd_3\mathbf{L}^{\mathbf{free}}_6[M\text{-}6BF_4^-]^{6+}$ (green), $Pd_4\mathbf{L}^{\mathbf{free}}_8[M\text{-}8BF_4^-]^{8+}$ (purple)) in CH_3CN.

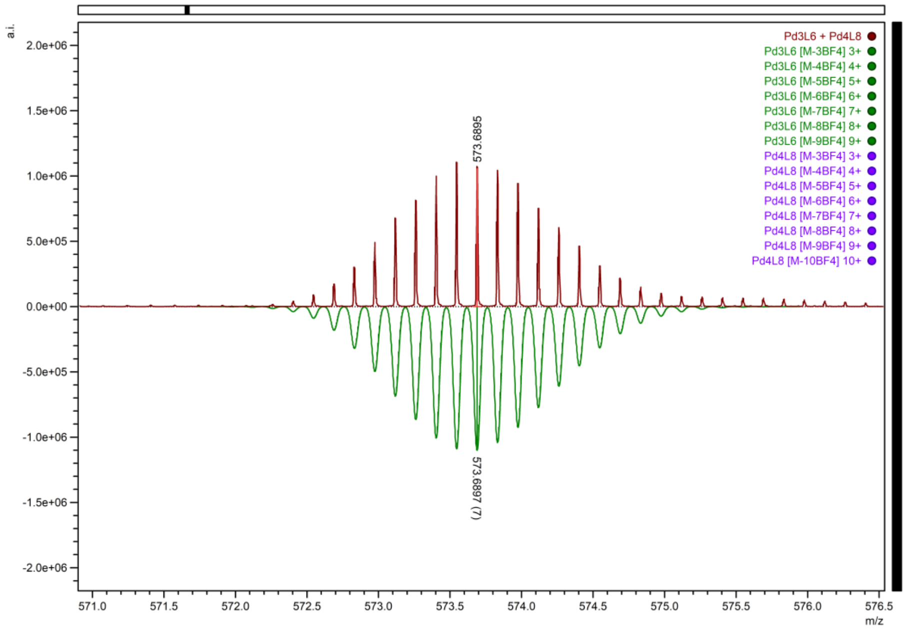

Figure S29. Observed charged species $Pd_3\mathbf{L}^{\mathbf{free}}_6[M\text{-}7BF_4^-]^{7+}$ (red) and simulated isotopic distribution (green) in CH_3CN.

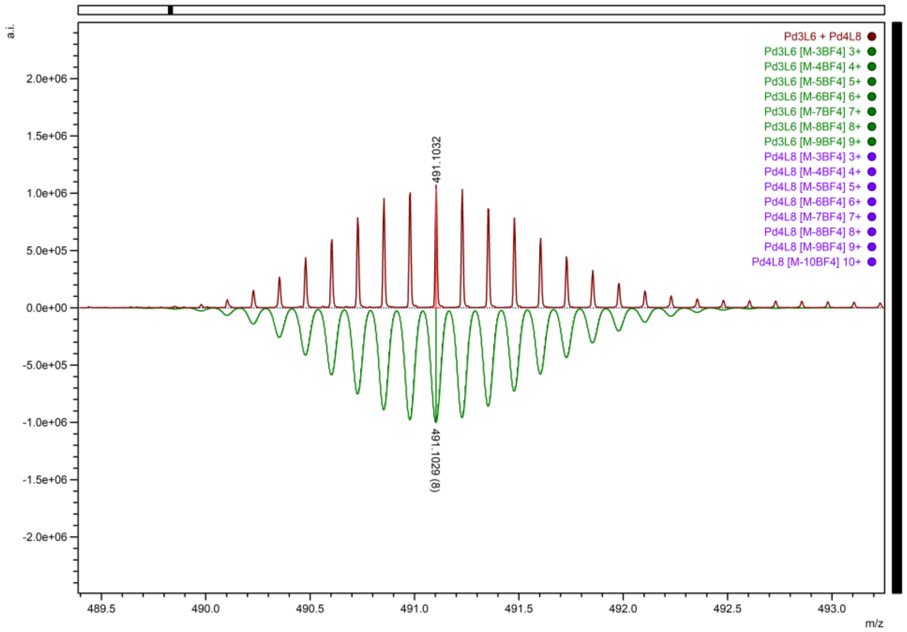

Figure S30. Observed charged species $Pd_3\mathbf{L}^{\mathbf{free}}_6[M\text{-}8BF_4^-]^{8+}$ (red) and simulated isotopic distribution (green) in CH_3CN.

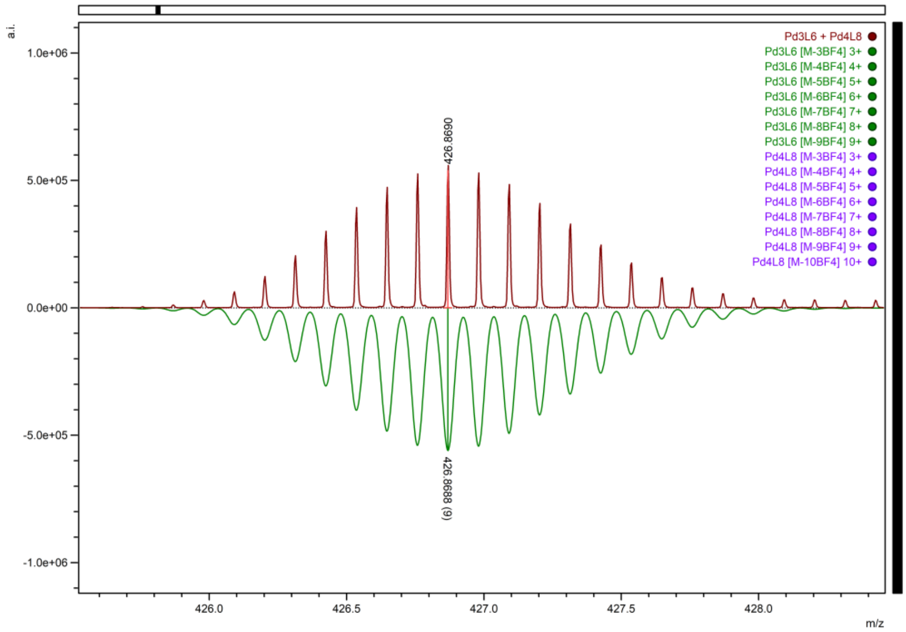

Figure S31. Observed charged species Pd_3**L**$^{free}{}_6[M\text{-}9BF_4^-]^{9+}$ (red) and simulated isotopic distribution (green) in CH_3CN.

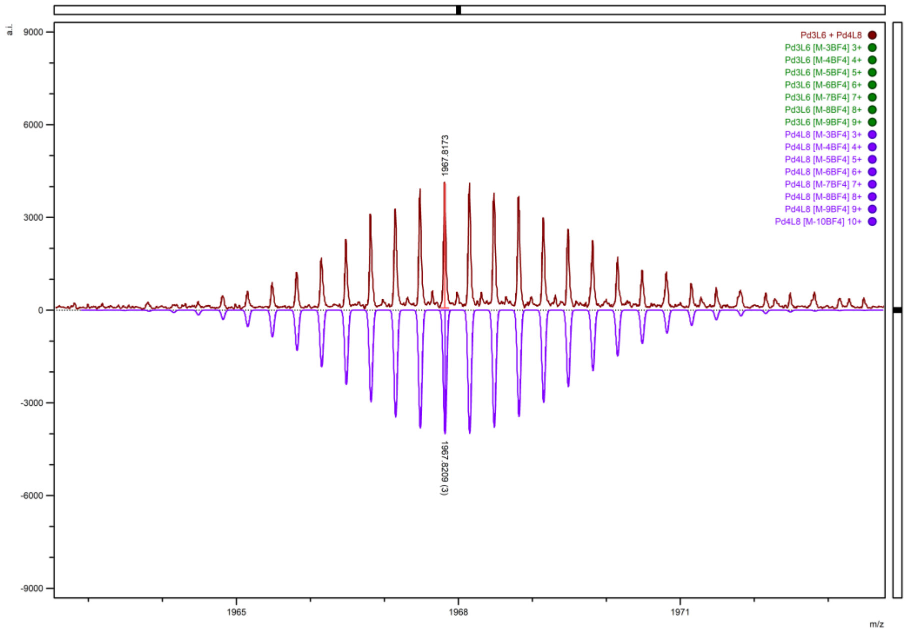

Figure S32. Observed charged species Pd_4**L**$^{free}{}_8[M\text{-}3BF_4^-]^{3+}$ (red) and simulated isotopic distribution (purple) in CH_3CN.

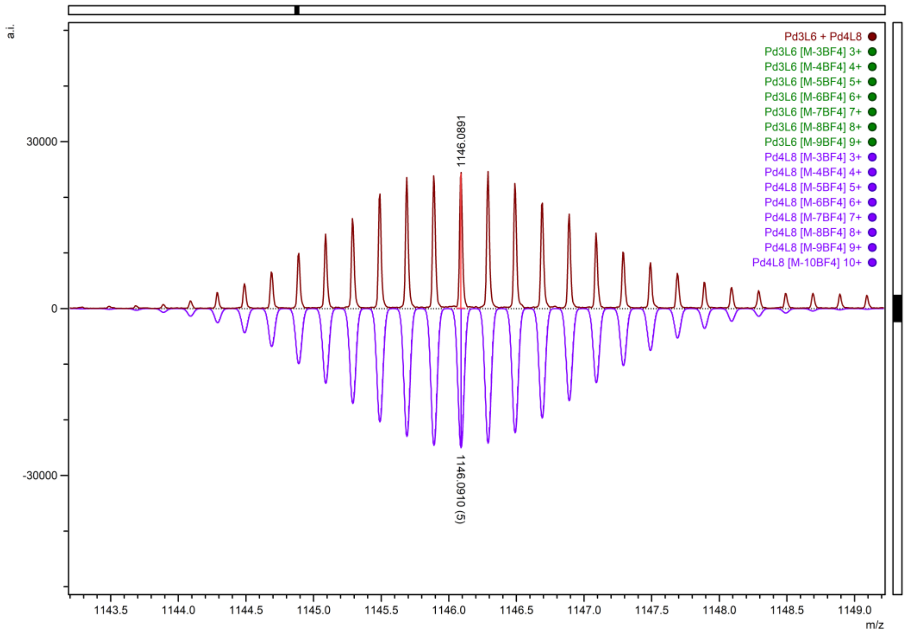

Figure S33. Observed charged species $Pd_4\mathbf{L}^{\mathbf{free}}{}_8[M-5BF_4^-]^{5+}$ (red) and simulated isotopic distribution (purple) in CH_3CN.

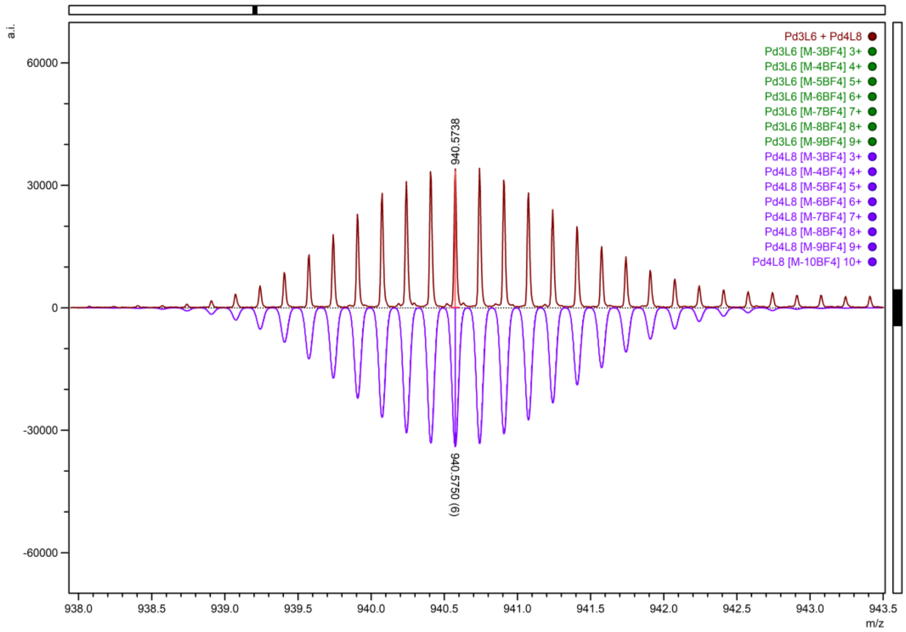

Figure S34. Observed charged species $Pd_4\mathbf{L}^{\mathbf{free}}{}_8[M-6BF_4^-]^{6+}$ (red) and simulated isotopic distribution (purple) in CH_3CN.

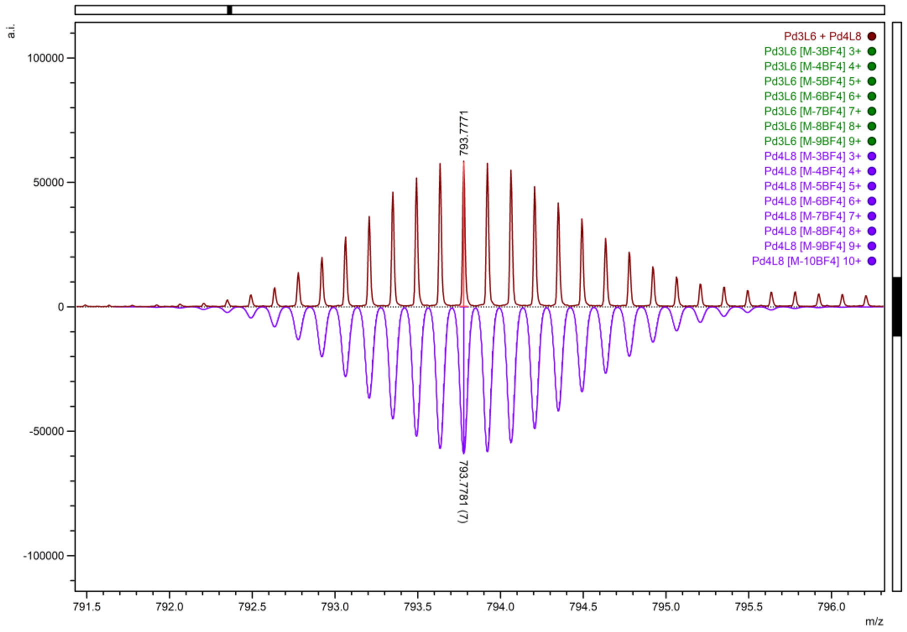

Figure S35. Observed charged species $Pd_4\mathbf{L}^{\mathbf{free}}_8[M\text{-}7BF_4^-]^{7+}$ (red) and simulated isotopic distribution (purple) in CH_3CN.

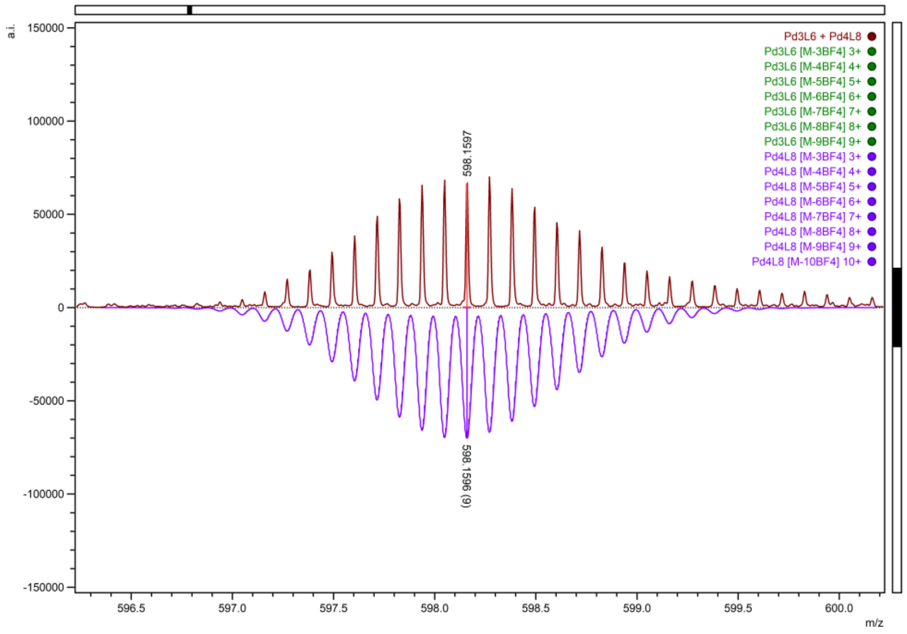

Figure S36. Observed charged species $Pd_4\mathbf{L}^{\mathbf{free}}_8$ $[M\text{-}9BF_4^-]^{9+}$ (red) and simulated isotopic distribution (purple) in CH_3CN.

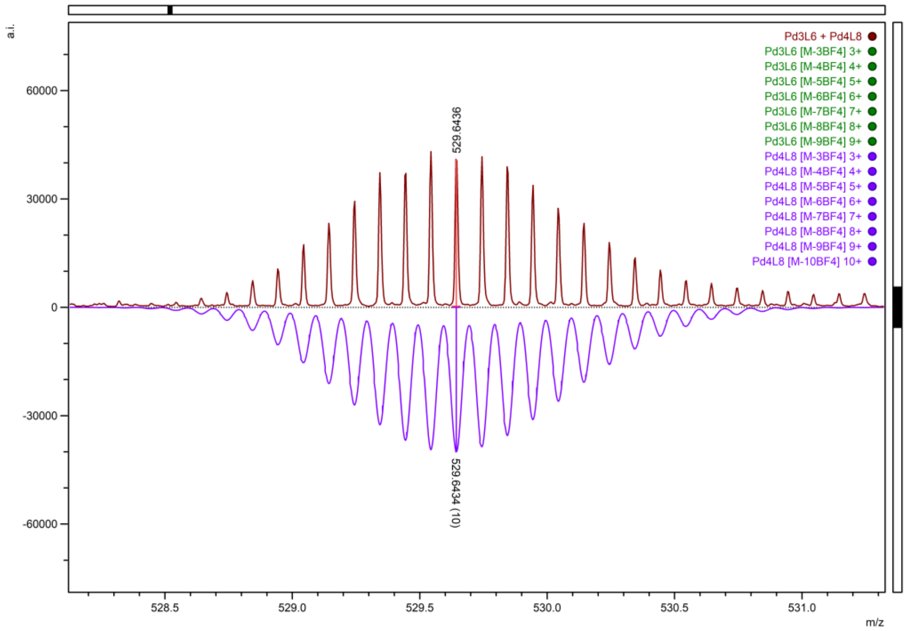

Figure S37. Observed charged species Pd_4**L**$^{\mathbf{free}}{}_8$ $[M-10BF_4^-]^{10+}$ (red) and simulated isotopic distribution (purple) in CH_3CN.

Synthesis of Au cage ($Pd_6L^{AuCl}_{12}$):

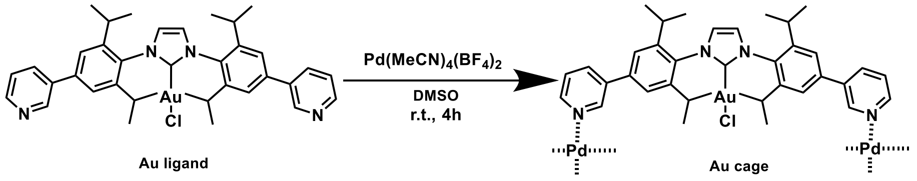

In a small vial, $\mathbf{L}^{\mathbf{AuCl}}$ (15.523 mg, 0.02 mmol) was dissolved in 1.8 mL DMSO-d_6, and then a solution containing $Pd(MeCN)_4(BF_4)_2$ (4.442 mg, 0.01 mmol) in 0.2 mL DMSO-d_6 was added, the solution was stirred at room temperature for 4 h to give a clear yellow solution. The solid product was collected by precipitaion in excess amount of diethyl ether with a yield of 82%. ^{1}H NMR (300 MHz, DMSO-d_6, ppm) δ 9.40 (s, 2H), 9.06 (s, 2H), 8.58 (s, 2H), 7.83 (s, 8H), 1.31 (s, 24H). ^{13}C NMR (126 MHz, DMSO-d_6, ppm) δ 175.59, 150.48, 148.75, 147.20, 139.74, 138.03, 136.22, 134.93, 127.34, 124.62, 122.95, 28.72, 23.29. ESI-MS：2162.6811 (calc. for $[Pd_6\mathbf{L}^{\mathbf{AuCl}}_{12}\cdot 5CD_3CN\cdot 2D_2O\cdot 19BF_4]^{5+}$ 2161.6150), 1786.8977 (calc. for $[Pd_6\mathbf{L}^{\mathbf{AuCl}}_{12}\cdot 5CD_3CN\cdot 2D_2O\cdot 18BF_4]^{6+}$ 1786.8450), 1509.9297 (calc. for $[Pd_6\mathbf{L}^{\mathbf{AuCl}}_{12}\cdot 4CD_3CN\cdot 17BF_4]^{7+}$ 1510.1415). DOSY NMR (DMSO-d_6, 298 K, log D = -10.10, R ≈ 2.75 nm).

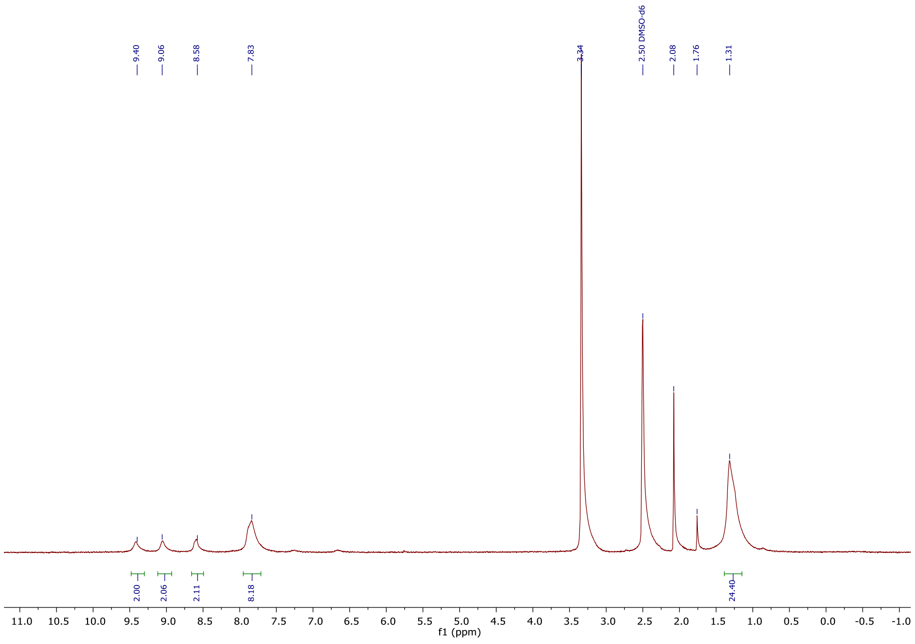

Figure S38. ^{1}H NMR spectrum (300 MHz, 298 K) of Au cage ($Pd_6\mathbf{L}^{\mathbf{AuCl}}_{12}$) in DMSO-$d_6$.

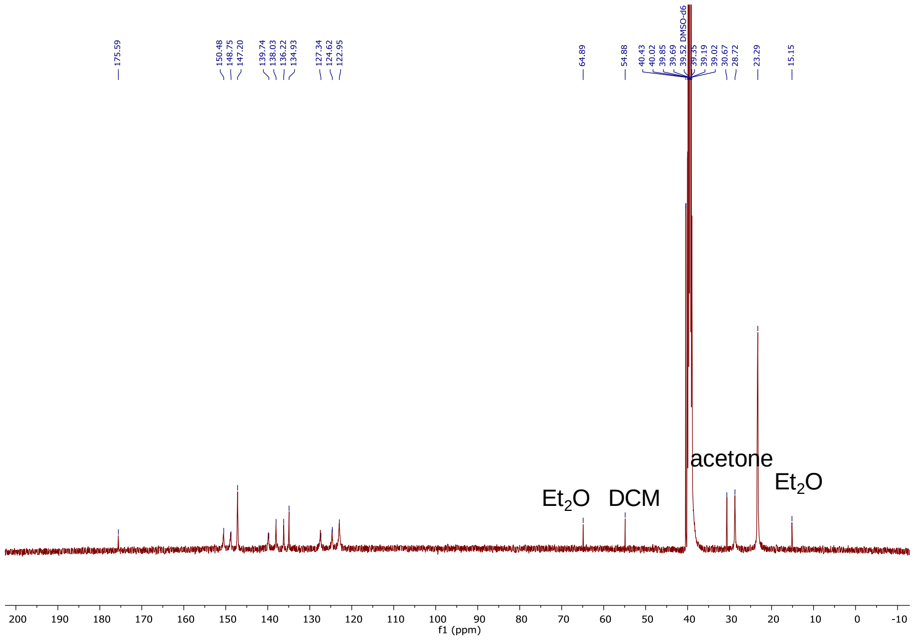

Figure S39. ^{13}C NMR spectrum (126 MHz, 298 K) of Au cage (Pd$_6$**L**$^{\mathbf{AuCl}}$$_{12}$) in DMSO-*d*$_6$.

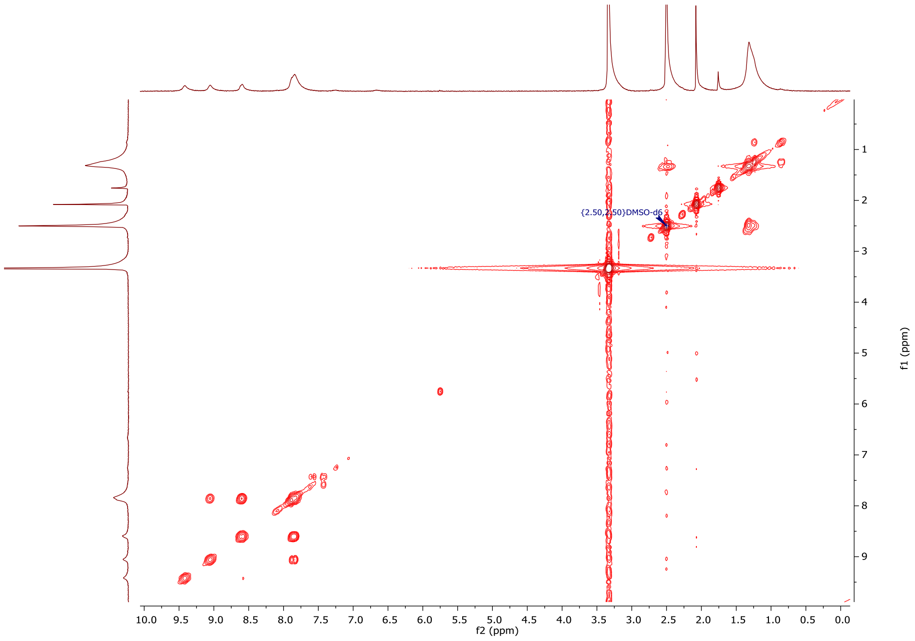

Figure S40. ^{1}H-^{1}H COSY spectrum (400 MHz, 298 K) of Au cage (Pd$_6$**L**$^{\mathbf{AuCl}}$$_{12}$) in DMSO-*d*$_6$.

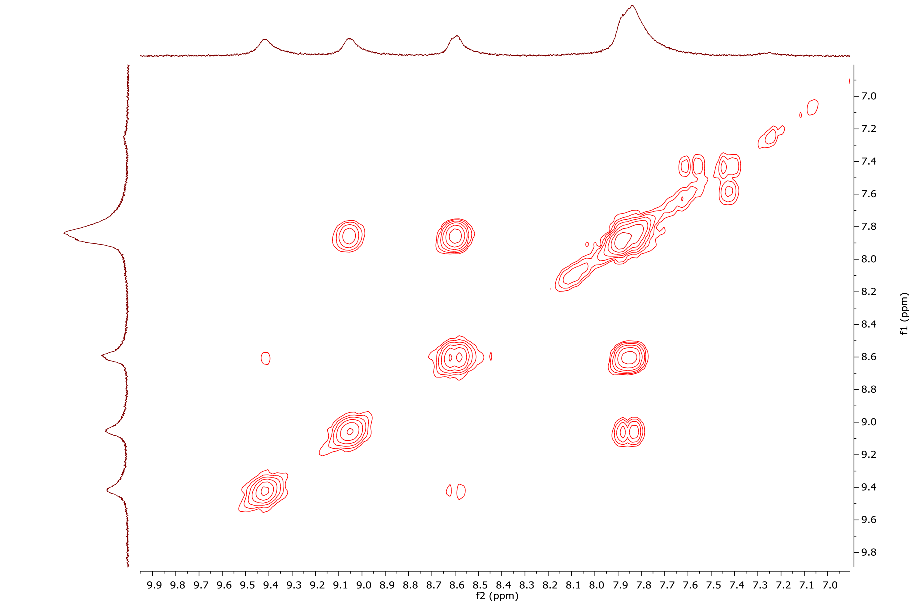

Figure S41. Aromatic area of ^{1}H-^{1}H COSY spectrum (400 MHz, 298 K) of Au cage ($Pd_6\mathbf{L}^{\mathbf{AuCl}}{}_{12}$) in DMSO-$d_6$.

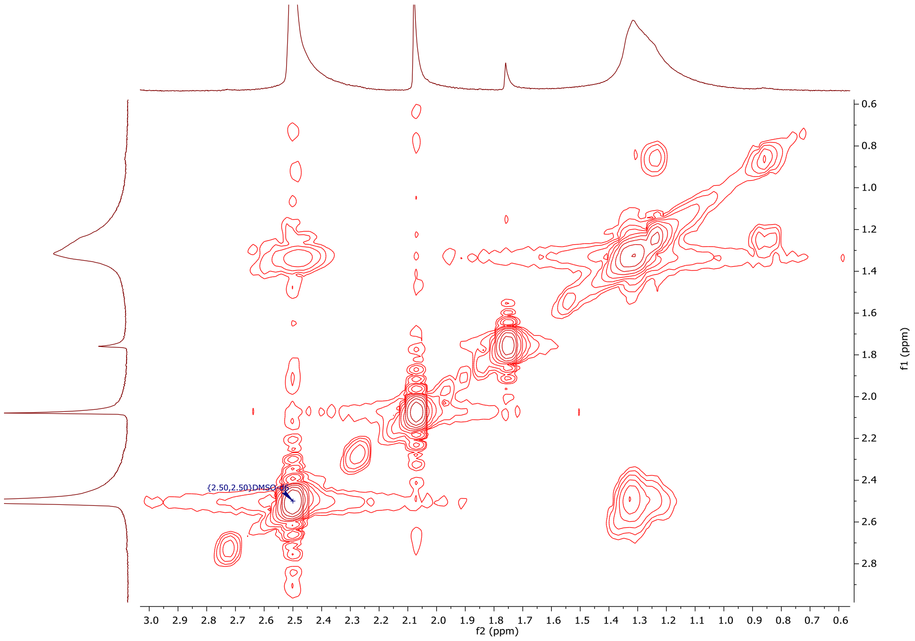

Figure S42. Aliphatic area of ^{1}H-^{1}H COSY spectrum (400 MHz, 298 K) of Au cage ($Pd_6\mathbf{L}^{\mathbf{AuCl}}{}_{12}$) in DMSO-$d_6$.

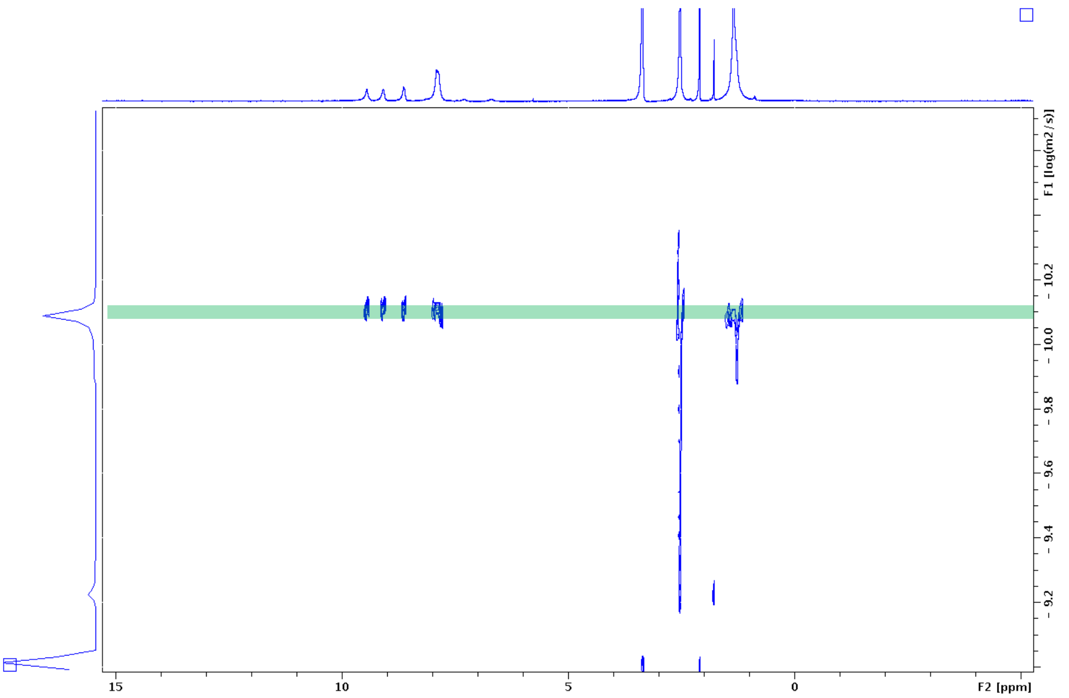

Figure S43. Overlapped ^{1}H DOSY spectrum (300 MHz, 298 K) of Pd_6**L**$^{\mathbf{AuCl}}{}_{12}$ in DMSO-d_6.

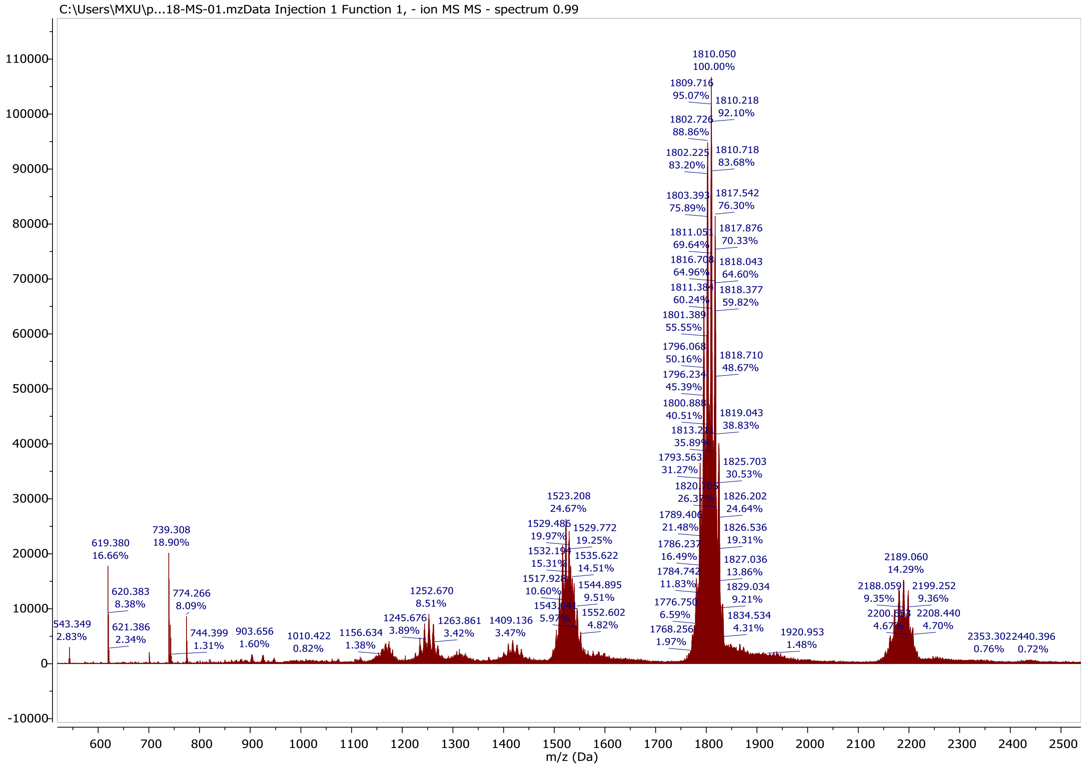

Figure S44. Mass spectrum of Pd_6**L**$^{\mathbf{AuCl}}{}_{12}$.

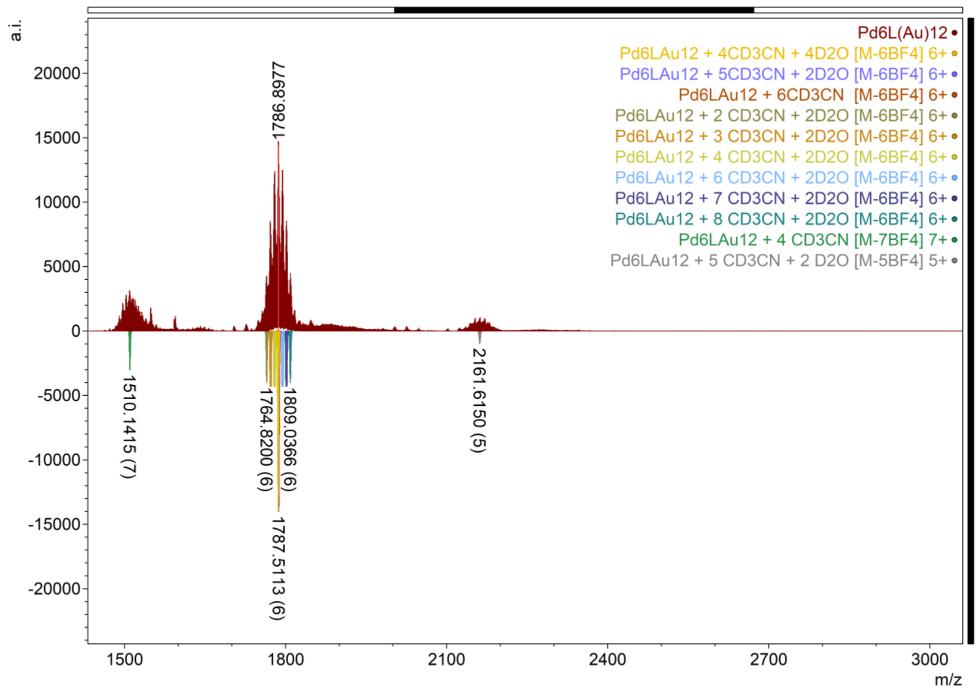

Figure S45. Full mass spectrum of $Pd_6\mathbf{L}^{AuCl}{}_{12}$ and simulated isotopic distribution of high charged species in CD_3CN.

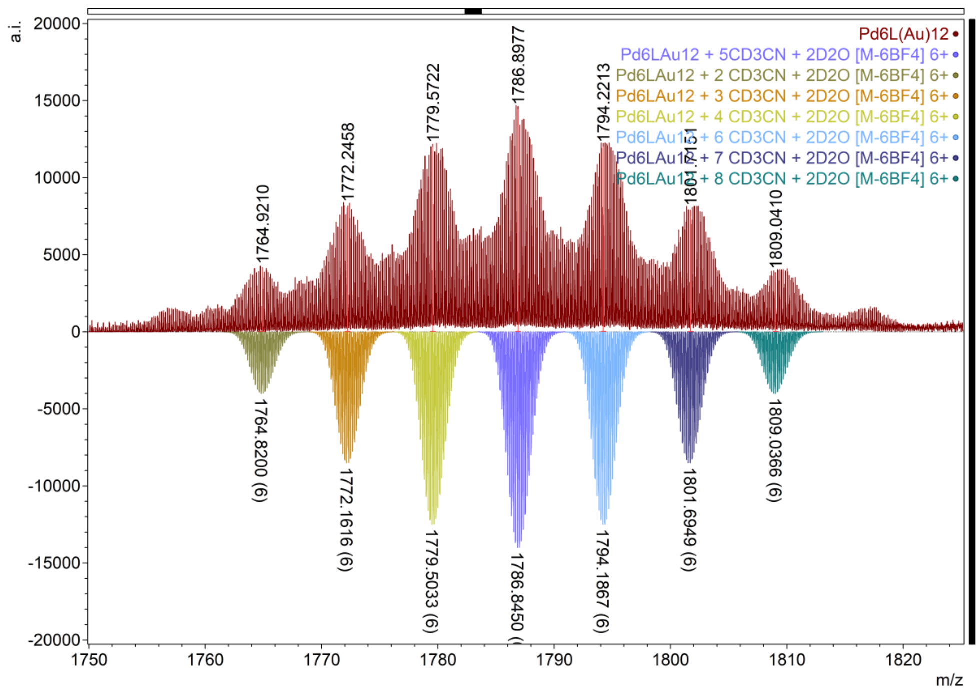

Figure S46. Mass spectrum of high charged species (+6) of $Pd_6\mathbf{L}^{AuCl}{}_{12}$ and simulated isotopic distribution containing different solvent in CD_3CN.

S3. Calculation

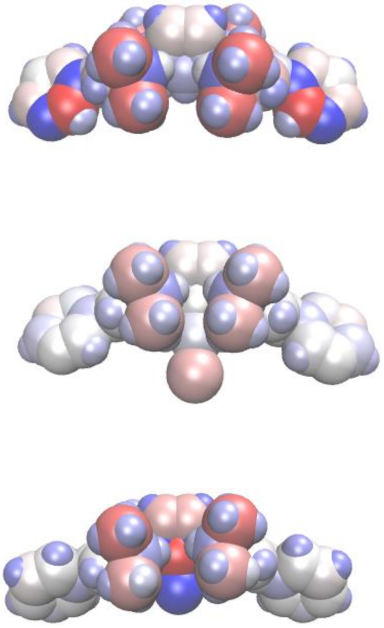

Figure S47. Charge structure of the ligands in different coordination states. Shown top to bottom: Charge assignments for $Pd_n\mathbf{L}^{\mathbf{free}}_{2n}$, $Pd_n\mathbf{L}^{\mathbf{AuCl}}_{2n}$ and $Pd_n\mathbf{L}^{\mathbf{Au}}_{2n}$ linkers with red signifying positive charge and blue negative. The coordination of the gold chloride had significant impacts on the charge density of the pyridyl groups, with the $Pd\mathbf{L}^{\mathbf{free}}$ linker featuring stronger polarization than the gold-coordinated congeners, which may produce significant interactions at the coordination nodes of the supramolecular construct.

S4. Catalysis and Kinetics studies

For all the catalytic studies, NHC-AuCl was purchased from Sigma Aldrich and used without further purification.

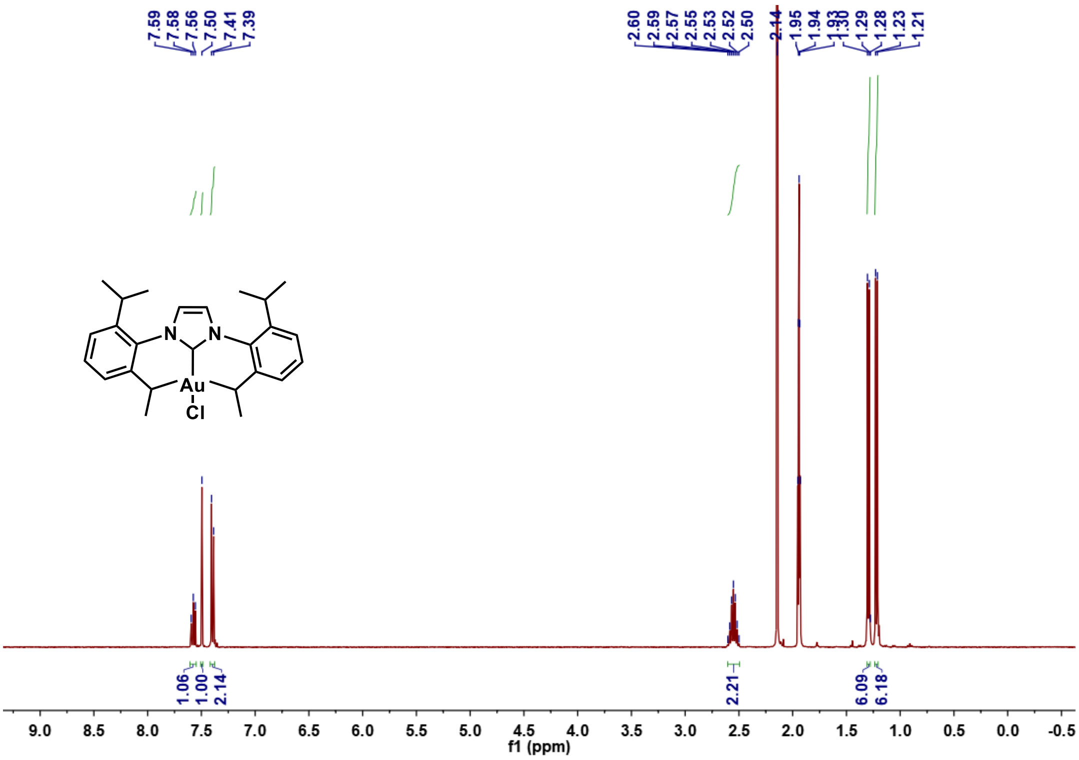

Figure S48. ^{1}H NMR spectrum (400 MHz, 298 K) of NHC-AuCl in CD_3CN.

Cyclization of allenol 1

All the catalytic reactions were carried out in CD_3CN at room temperature for 25 h under Ar. 1,3,5-trimethoxybenzene was used as an internal standard. Stock solutions of $AgBF_4$, NHC-AuCl, NHC-Au^+, Pd_3**L**$^{\mathbf{free}}_6$, Pd_6**L**$^{\mathbf{AuCl}}_{12}$, Pd_6**L**$^{\mathbf{Au}}_{12}$, internal standard and allenol (substrate **1**) were prepared in CD_3CN, respectively. Activated NHC-Au^+ species and Pd_6**L**$^{\mathbf{Au}}_{\mathbf{12}}$ were generated by in situ activation upon addition one equivalent of $AgBF_4$ in CD_3CN and stirred at room temperature for 12 h. The solution was then filtered for catalytic reactions. The certain volumes of each stock solution were taken directly and mixed in a NMR tube for each entry in **Table S1** (pre-dried CD_3CN as solvent) and **Table S2** (hydrous CD_3CN as solvent). The reaction of each entry contained catalyst (0.0015 mmol), 1,3,5-trimethoxybenzene (0.005 mmol) and substrate **1** (0.03 mmol) with a total volume of 0.6 mL. Each reaction was carried out for 2-3 times and gave reproducible results (error limit estimated to be 5%).

Reaction conditions of each effector in each reaction :

1. n(NHC-AuCl) = 0.0015 mmol
2. n(NHC-Au^+) = 0.0015 mmol
3. n(Pd_6**L**$^{\mathbf{AuCl}}_{\mathbf{12}}$) = 0.0015 mmol (based on the ligands)
4. n(Pd_6**L**$^{\mathbf{Au}}_{\mathbf{12}}$) = 0.0015 mmol (based on the ligands)
5. n(Pd_3**L**$^{\mathbf{free}}_{\mathbf{6}}$) = 0.0015 mmol (based on the ligands)
6. n(Pd_3**L**$^{\mathbf{free}}_{\mathbf{6}}$ + $AgBF_4$) = 0.0015 mmol (based on the ligands)
7. n($AgBF_4$) = 0.0015 mmol
8. n(1,3,5-trimethoxybenzene) = 0.005 mmol
9. n(**1**) = 0.03 mmol

Table S1. Control experiments of gold-catalyzed cyclization of allenol **1** under anhydrous conditions.

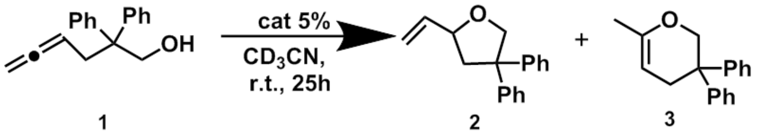

Entry	conditions	**2** [%]	**3** [%]
1	$AgBF_4$	0	0
2	NHC-AuCl	0	0
3	NHC-Au^+	30	0
4	$Pd_3\mathbf{L}^{\mathbf{free}}_6$	0	0
5	$Pd_3\mathbf{L}^{\mathbf{free}}_6$+$AgBF_4$	0	0
6	$Pd_6\mathbf{L}^{\mathbf{AuCl}}_{12}$	0	0
7	$Pd_6\mathbf{L}^{\mathbf{Au}}_{12}$	57	0

Table S2. Control experiments of gold-catalyzed cyclization of allenol **1** under hydrous conditions.

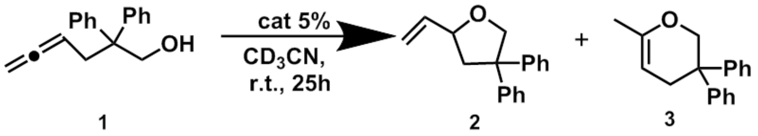

Entry	Conditions	**2** [%]	**3** [%]
1	NHC-Au^+	13	0
2	$Pd_6\mathbf{L}^{\mathbf{Au}}_{12}$	57	0

The known products in cyclization of allenol **1** (**Tables S1** and **S2**) were monitored by ^1H NMR and identified by direct comparison to the literature, **2**,[5] **3**.[5]

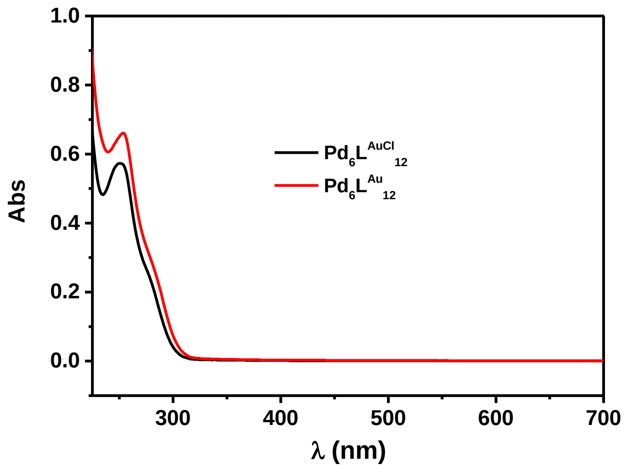

Figure S49. Normalized UV-Vis spectra of $Pd_6\mathbf{L}^{\mathbf{AuCl}}_{12}$ and $Pd_6\mathbf{L}^{\mathbf{Au}}_{12}$ in CH_3CN at room temperature. According to the literature,[4] if the gold sites are not separated in cage, the sites would experience d^{10}-d^{10} aurophilic interactions which leads to a broad shoulder at about 315 nm in UV-Vis spectrum. Since no shoulder at 315 nm was found in Figure 49, it suggests that the gold sites are separated in $Pd_6\mathbf{L}^{\mathbf{AuCl}}_{12}$ and $Pd_6\mathbf{L}^{\mathbf{Au}}_{12}$.

Circular catalytic reactions of cyclization of allenol 1 in kinetic analysis

To evaluate the stability and reactivity of the $Pd_6\mathbf{L}^{\mathbf{Au}}_{12}$ and NHC-Au^+, a circular reaction upon addition of two batches of substrate was operated. All the kinetic reactions were carried out at room temperature under argon conditions in pre-dried and wet CD_3CN, respectively. Reaction conditions of each effector in each reaction were as the same as those in **Table S2**. The second batch of substrate (allenol **1**, 0.03 mmol) was added at 49 h in each reaction. All the reactions were monitored by ^{1}H NMR.

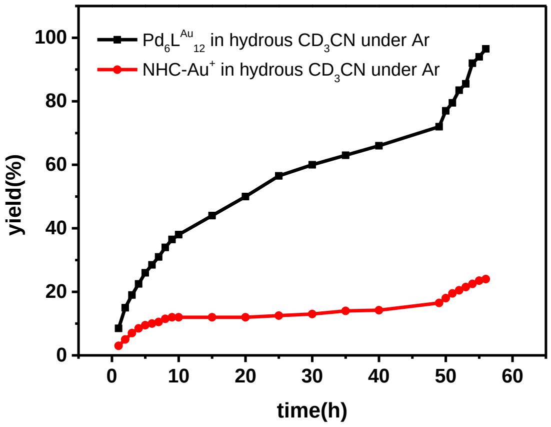

Figure S50. Reaction profiles for the yield of product **2** in hydrous CD_3CN under Ar.

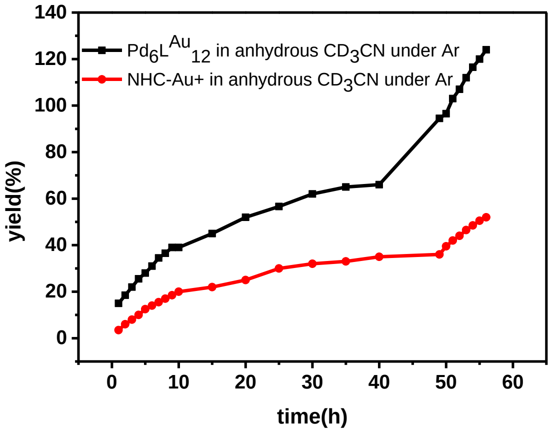

Figure S51. Reaction profiles for the yield of product **2** in anhydrous CD_3CN under Ar.

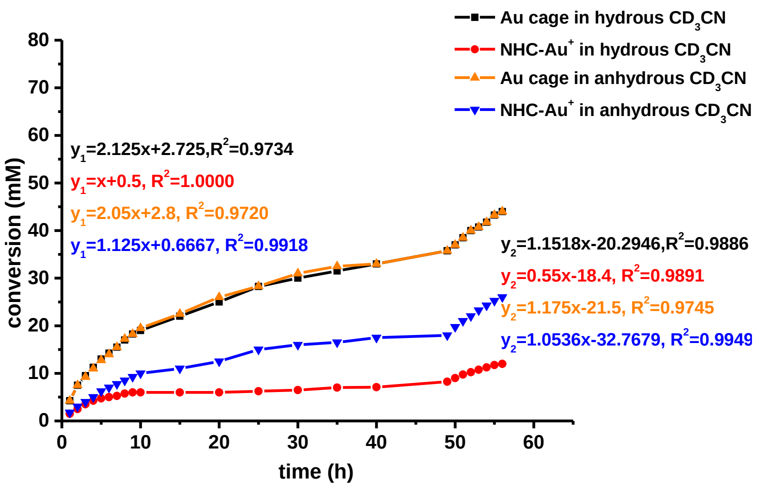

Figure S52. Reaction profiles for the conversion of substrate **1** (mM) in CD_3CN under Ar (monitored by ^1H NMR).

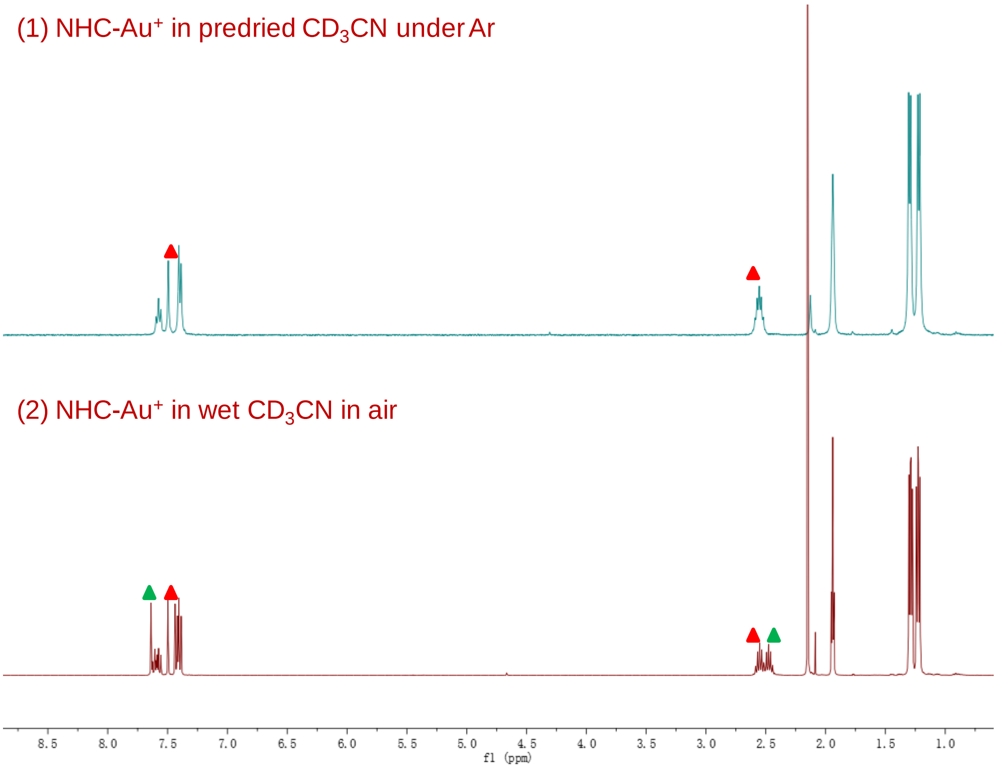

Figure S53. ^1H NMR (400 MHz, 298 K) of NHC-Au^+ in pre-dried CD_3CN (1) and wet CD_3CN (2).

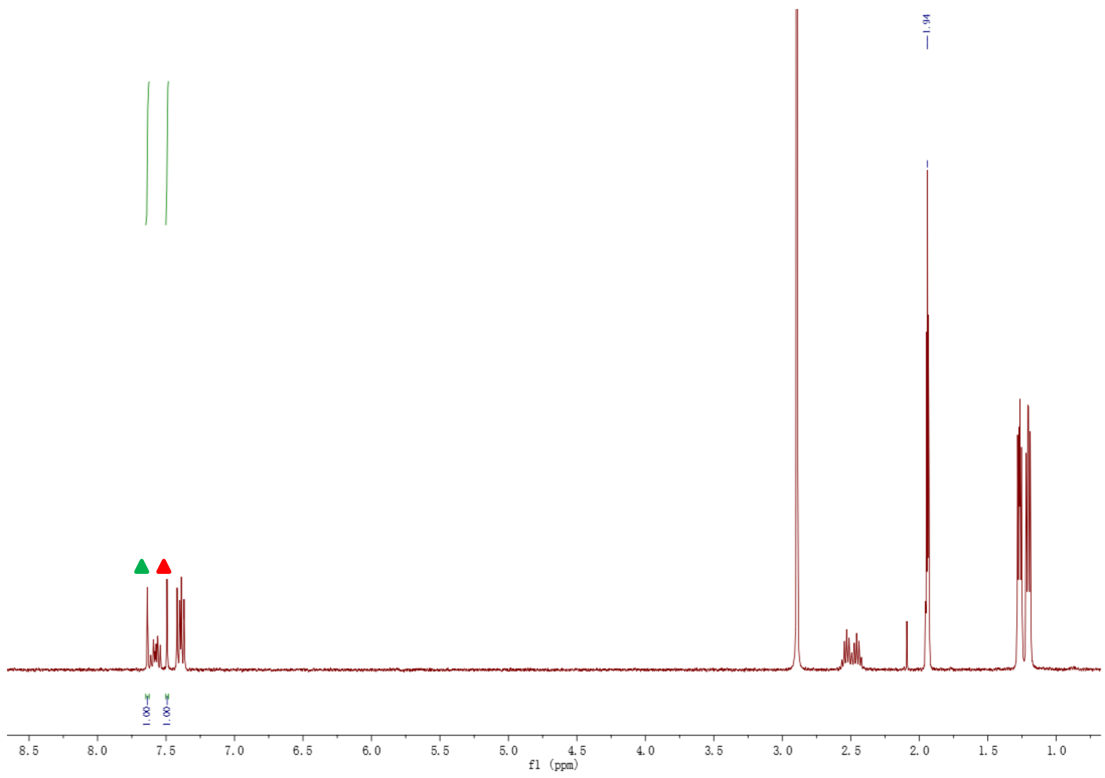

Figure S54. ^{1}H NMR (400 MHz, 298 K) of NHC-Au^{+} mixing with D_2O in wet CD_3CN at rt for 24 h.

Cyclization of hex-4-ynoic acid 4

All the catalytic reactions were carried out in a mixture of solvents (DMSO-d_6/CD_2Cl_2 = 1:3) at room temperature for 10 min under N_2. 1,3,5-trimethoxybenzene was used as an internal standard. Stock solutions of $AgBF_4$, NHC-AuCl, NHC-Au^+, $Pd_3\mathbf{L}^{\mathbf{free}}_6$, $Pd_6\mathbf{L}^{\mathbf{AuCl}}_{12}$, and $Pd_6\mathbf{L}^{\mathbf{Au}}_{12}$ were prepared in DMSO-d_6. Activated NHC-Au^+ species and $Pd_6\mathbf{L}^{\mathbf{Au}}_{12}$ were generated by in situ activation upon addition of one equivalent of $AgBF_4$ in DMSO-d_6 and stirred overnight. The solution was then filtered for catalytic reactions. Stock solutions of substrate (hex-4-ynoic acid **4**) and internal standard were made in CD_2Cl_2, respectively. The certain volumes of stock solutions were taken directly and mixed in a NMR tube for every entry in **Table S3**. The reaction of each entry contained catalyst (0.0015 mmol), 1,3,5-trimethoxybenzene (0.005 mmol) and substrate **4** (0.03 mmol) with a total volume of 0.6 mL. Each reaction was carried out for 2-3 times and gave reproducible results (error limit estimated to be 5%).

Reaction conditions of each effector in each reaction :

1. n(NHC-AuCl) = 0.0015 mmol
2. n(NHC-Au^+) = 0.0015 mmol
3. n($Pd_6\mathbf{L}^{\mathbf{AuCl}}_{12}$) = 0.0015 mmol (based on the ligands)
4. n($Pd_6\mathbf{L}^{\mathbf{Au}}_{12}$) = 0.0015 mmol (based on the ligands)
5. n($Pd_3\mathbf{L}^{\mathbf{free}}_6$) = 0.0015 mmol (based on the ligands)
6. n($Pd_3\mathbf{L}^{\mathbf{free}}_6$ + $AgBF_4$) = 0.0015 mmol (based on the ligands)
7. n($AgBF_4$) = 0.0015 mmol
8. n(1,3,5-trimethoxybenzene) = 0.005 mmol
9. n(**4**) = 0.03 mmol

Table S3. Control experiments of gold-catalyzed cyclization of hex-4-ynoic acid **4** under anhydrous conditions.

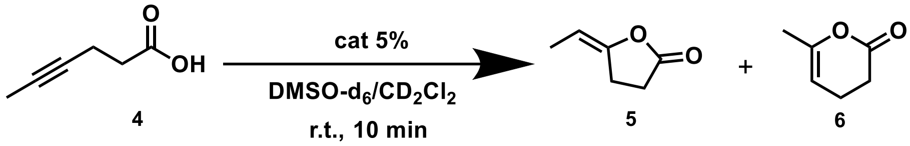

Entry	Conditions	Conv.[%]	**5**[%]	**6**[%]	**5**/**6**
1	$AgBF_4$	0	0	0	-
2	NHC-AuCl	0	0	0	-
3	NHC-Au^+	100	40	60	0.7
4	$Pd_3\mathbf{L}^{\mathbf{free}}_6$	0	0	0	-
5	$Pd_3\mathbf{L}^{\mathbf{free}}_6$+$AgBF_4$	0	0	0	-
6	$Pd_6\mathbf{L}^{\mathbf{AuCl}}_{12}$	0	0	0	-
7	$Pd_6\mathbf{L}^{\mathbf{Au}}_{12}$	100	54	45	1.2

The known products in cyclization of hex-4-ynoic acid **4** were monitored by ^{1}H NMR and identified by direct comparison to the literature, **5**,[6] **6**.[6]

Kinetic analysis using the Michaelis-Menten mechanism

The kinetic experiments of cyclizations of allenol and hex-4-ynoic acid were monitored by ^{1}H NMR in anhydrous solvents under Ar. The uncatalyzed reactions fit well to the pseudo first order rate equation to obtain the uncatalyzed rate constants k'_{uncat} (2.11×10^{-8} s^{-1}) for allenol, and k''_{uncat} (2.18×10^{-7} s^{-1}) for hex-4-ynoic acid. The ratio of k'_{cage}/k'_{uncat} was found as 6.11×10^{4}, and k''_{cage}/k''_{uncat} was found as 6.19×10^{4}.

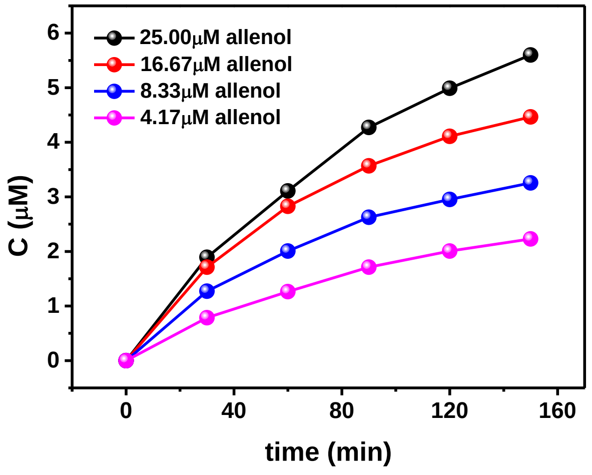

Figure S55. Kinetics with varying concentrations of allenol (4.17 μM, 8.33 μM, 16.67 μM, 25.00 μM) for Pd_6**L**$^{\mathbf{Au}}_{12}$ cage (0.83 μM, based on the ligand).

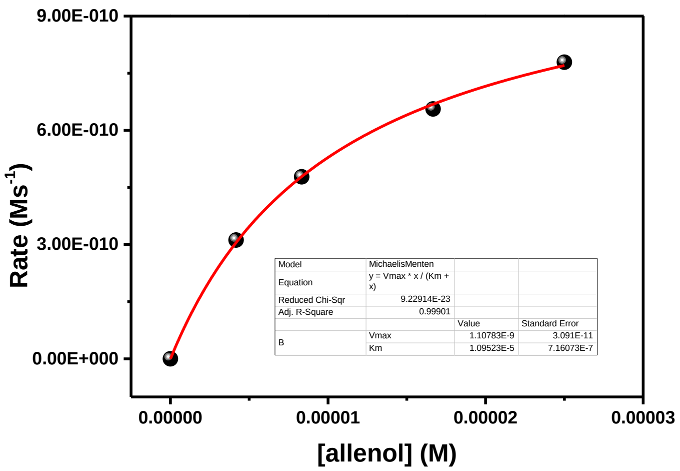

Figure S56. Steady-state kinetic analyses of allenol cyclizations with varying concentrations of substrate (4.17 μM, 8.33 μM, 16.67 μM, 25.00 μM) using the Michaelis-Menten model for $Pd_6\mathbf{L}^{\mathbf{Au}}{}_{\mathbf{12}}$ (0.83 μM, based on the ligand).

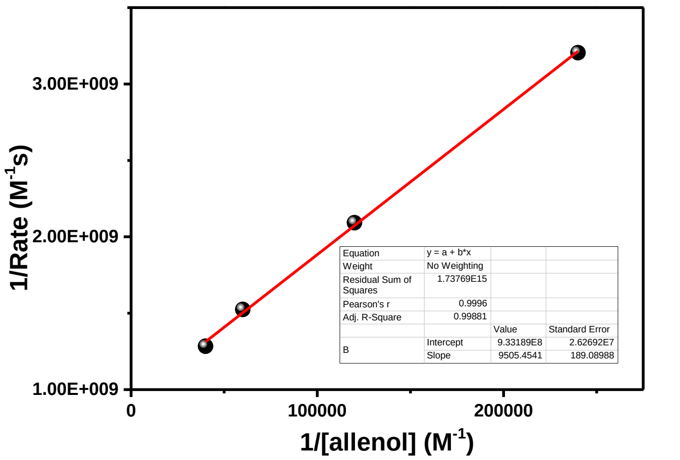

Figure S57. Lineweaver-Burk plot in cyclization of allenol for $Pd_6\mathbf{L}^{\mathbf{Au}}{}_{\mathbf{12}}$. The k'_{cage} was found to be 1.29×10^{-3} s^{-1}.

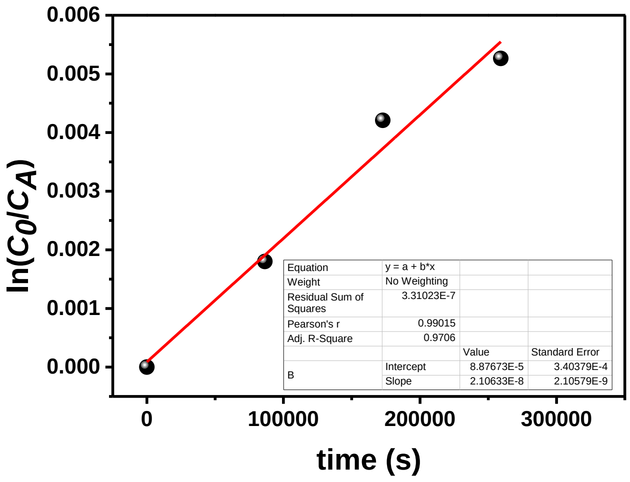

Figure S58. Measurement of the background (uncatalyzed) first order of cyclization reaction of allenol. The uncatalyzed rate constants k'_{uncat} was obtained (2.11×10^{-8} s^{-1}) for allenol. The ratio of k'_{cage}/k'_{uncat} was found as 6.11×10^{4}.

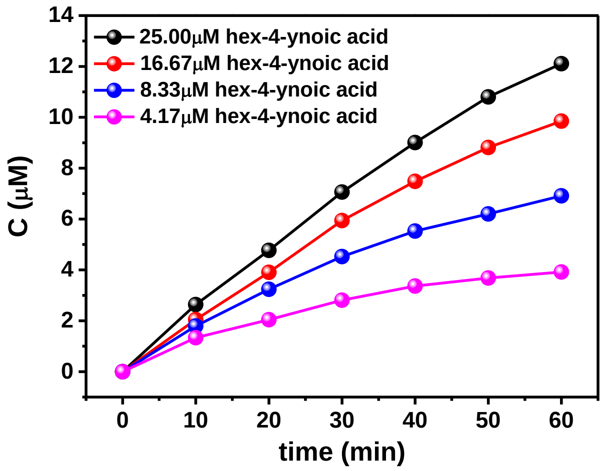

Figure S59. Kinetics of the total conversions of hex-4-ynoic acid with varying concentrations of substrate (4.17 μM, 8.33 μM, 16.67 μM, 25.00 μM) for $Pd_6\mathbf{L}^{Au}_{12}$ cage (0.42 μM, based on the ligand).

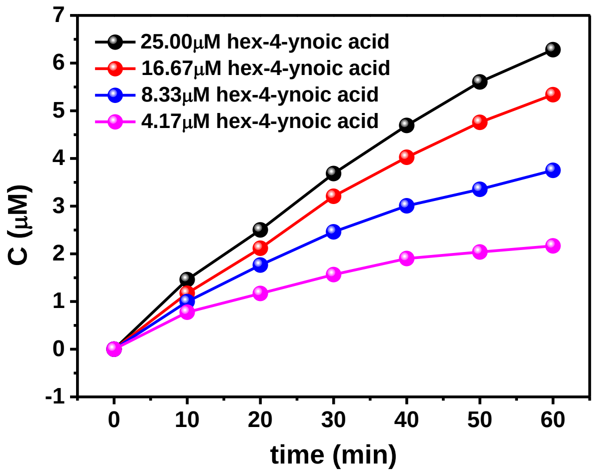

Figure S60. Kinetics of the yields of 5-membered ring (product **5**) with varying concentrations of hex-4-ynoic acid (4.17 μM, 8.33 μM, 16.67 μM, 25.00 μM) for Pd_6**L**^{Au}$_{12}$ (0.42 μM, based on the ligand).

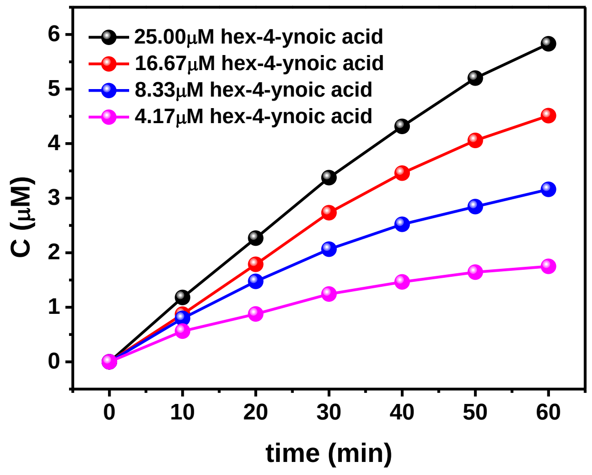

Figure S61. Kinetics of the yields of 6-membered ring (product **6**) with varying concentrations of hex-4-ynoic acid (4.17 μM, 8.33 μM, 16.67 μM, 25.00 μM) for Pd_6**L**^{Au}$_{12}$ (0.42 μM, based on the ligand).

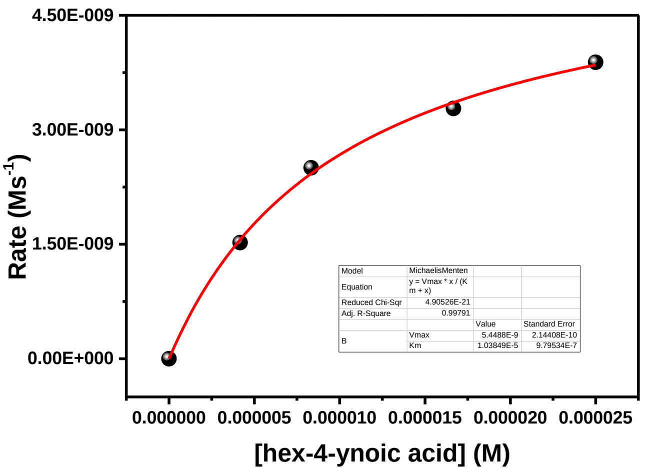

Figure S62. Steady-state kinetic analyse of hex-4-ynoic acid cyclization using the Michaelis-Menten model for $Pd_6\mathbf{L}^{\mathbf{Au}}{}_{\mathbf{12}}$ (0.42 μM, based on the ligand).

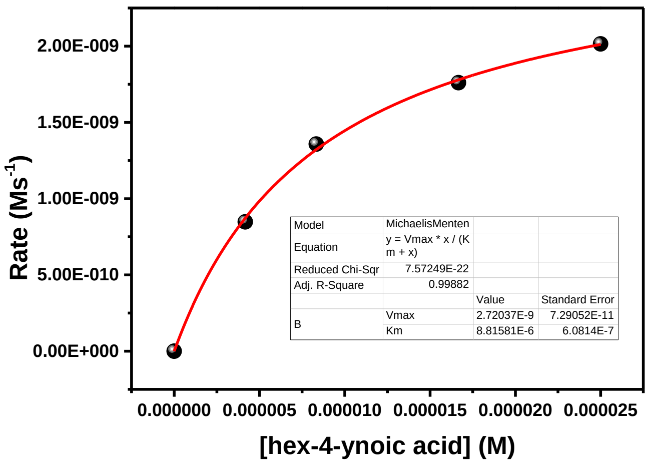

Figure S63. Steady-state kinetic analyse of the yield of 5-membered ring (product **5**) in cyclization of hex-4-ynoic acid using the Michaelis Menten model for $Pd_6\mathbf{L}^{\mathbf{Au}}{}_{12}$ (0.42 μM, based on the ligand).

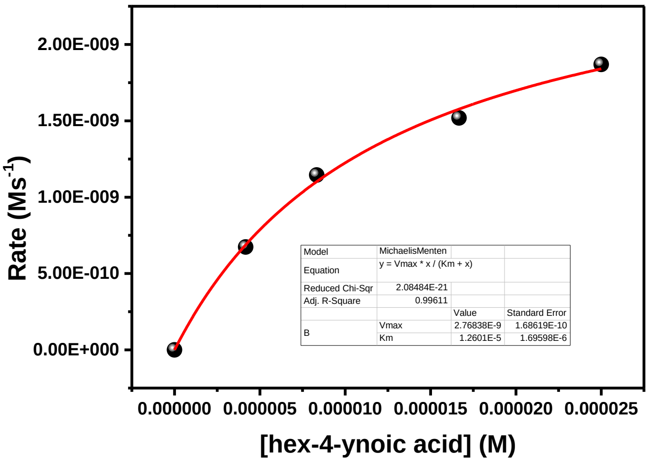

Figure S64. Steady-state kinetic analyse of the yield of 6-membered ring (product **6**) in cyclization of hex-4-ynoic acid using the Michaelis-Menten model for $Pd_6\mathbf{L}^{\mathbf{Au}}_{12}$ (0.42 μM, based on the ligand).

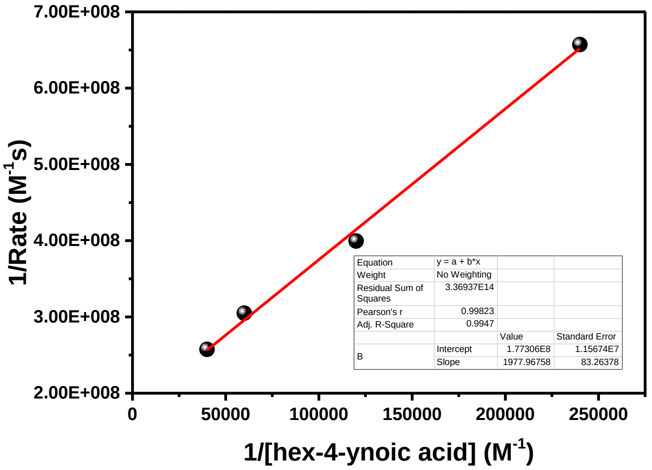

Figure S65. Lineweaver-Burk plot of the conversion of hex-4-ynoic acid in cyclization for $Pd_6\mathbf{L}^{\mathbf{Au}}_{12}$ (0.42 μM, based on the ligand). The k''_{cage} was found to be 1.35×10^{-2} s^{-1}.

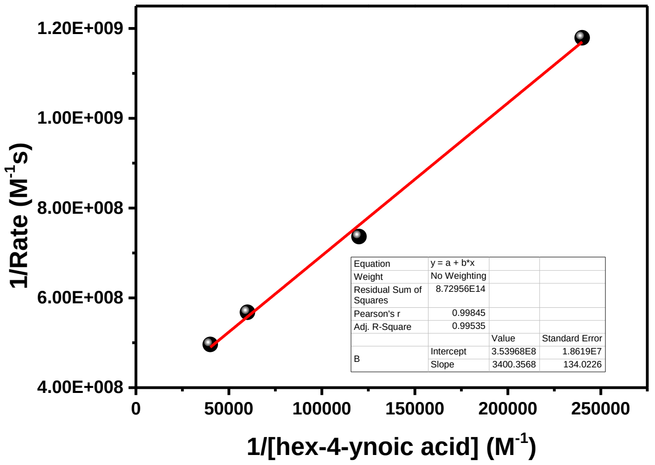

Figure S66. Lineweaver-Burk plot of the yield of 5-membered ring (product **5**) in cyclization for $Pd_6\mathbf{L}^{\mathbf{Au}}_{12}$ (0.42 μM, based on the ligand). ($k_{5\text{-}membered\ ring} \approx 7 \times 10^{-3}$ s^{-1}, $K_m \approx 9.6 \times 10^{-6}$ M, $V_{max} \approx 3 \times 10^{-9}$).

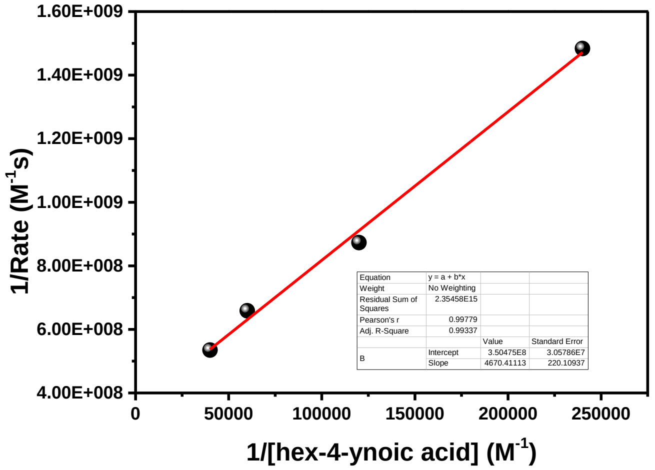

Figure S67. Lineweaver-Burk plot of the yield of 6-membered ring (product **6**) in cyclization for $Pd_6\mathbf{L}^{\mathbf{Au}}_{12}$ (0.42 μM, based on the ligand). ($k_{6\text{-}membered\ ring} \approx 7 \times 10^{-3}$ s^{-1}, $K_m \approx 1.3 \times 10^{-5}$ M, $V_{max} \approx 3 \times 10^{-9}$).

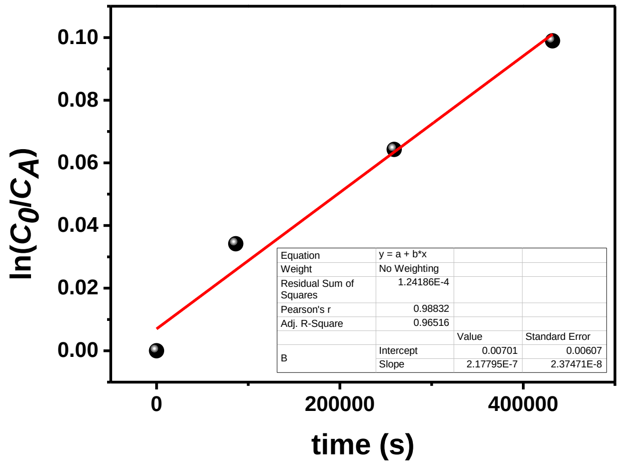

Figure S68. Measurement of the background (uncatalyzed) first order of cyclization reaction of hex-4-ynoic acid. The uncatalyzed rate constants k''_{uncat} (2.18×10^{-7} s^{-1}) for hex-4-ynoic acid. The ratio of k''_{cage}/k''_{uncat} was found as 6.19×10^{4}.

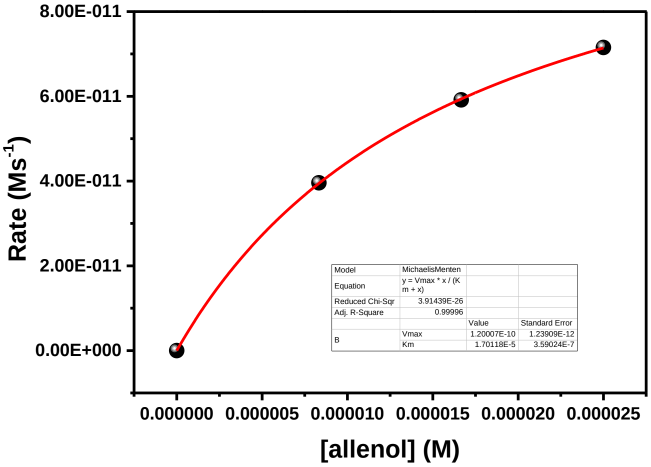

Figure S69. Steady-state kinetic analyses of allenol cyclizations with varying concentrations of substrate (8.33 µM, 16.67 µM, 25.00 µM) using the Michaelis-Menten model for NHC-Au^{+} (0.83 µM).

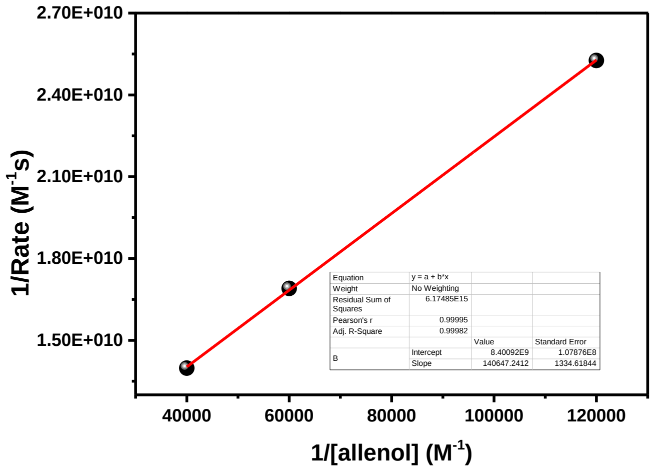

Figure S70. Lineweaver-Burk plot in cyclization of allenol for NHC-Au$^+$. The $k'_{NHC\text{-}Au}$ was found to be 1.43 $\times 10^{-4}$ s^{-1}.

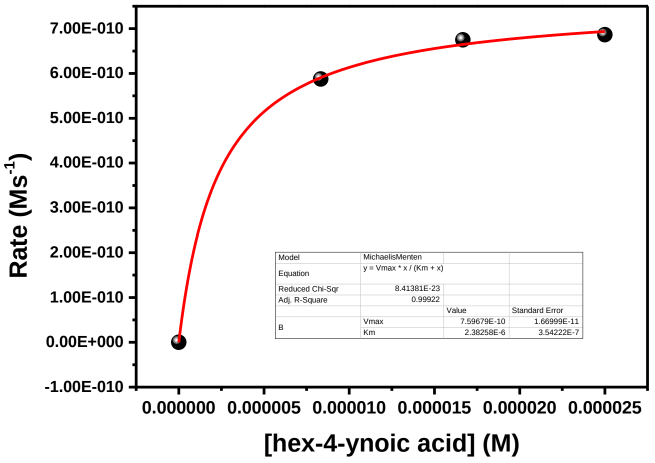

Figure S71. Steady-state kinetic analyses of hex-4-ynoic acid cyclizations with varying concentrations of substrate (8.33 μM, 16.67 μM, 25.00 μM) using the Michaelis-Menten model for NHC-Au$^+$ (0.42 μM).

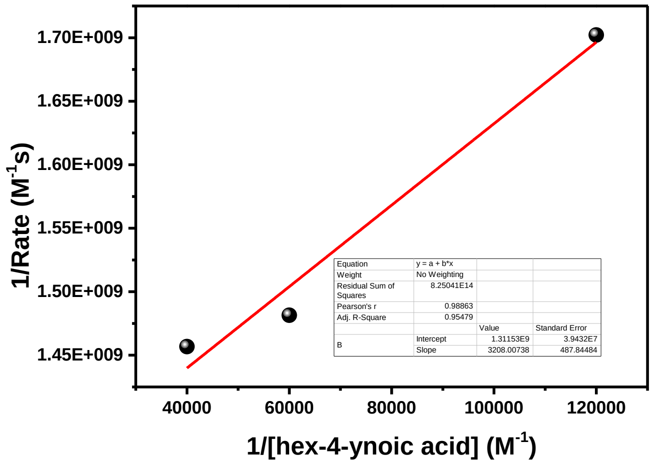

Figure S72. Lineweaver-Burk plot in cyclization of hex-4-ynoic acid for NHC-Au^+. The $k''_{NHC\text{-}Au}$ was found to be 1.83 $\times 10^{-3}$ s^{-1}.

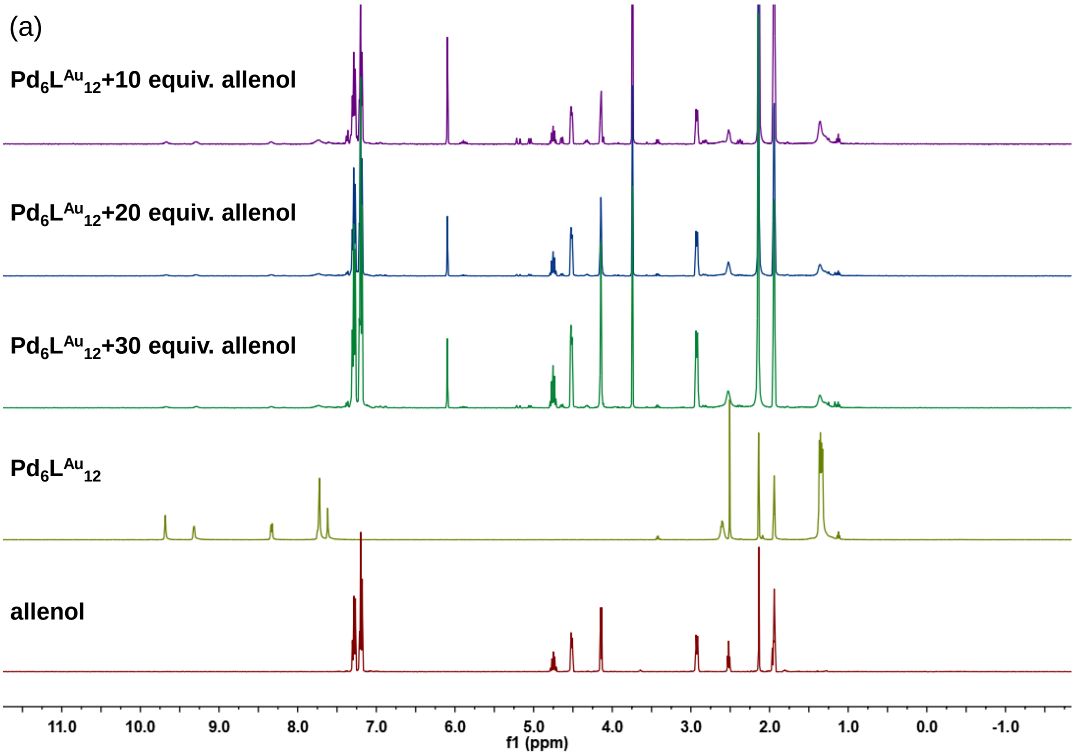

(a)
Pd6LAu12+10 equiv. allenol
Pd6LAu12+20 equiv. allenol
Pd6LAu12+30 equiv. allenol
Pd6LAu12
allenol
11.0
10.0
9.0
8.0
7.0
6.0
5.0
4.0
3.0
2.0
1.0
0.0
-1.0
f1 (ppm)

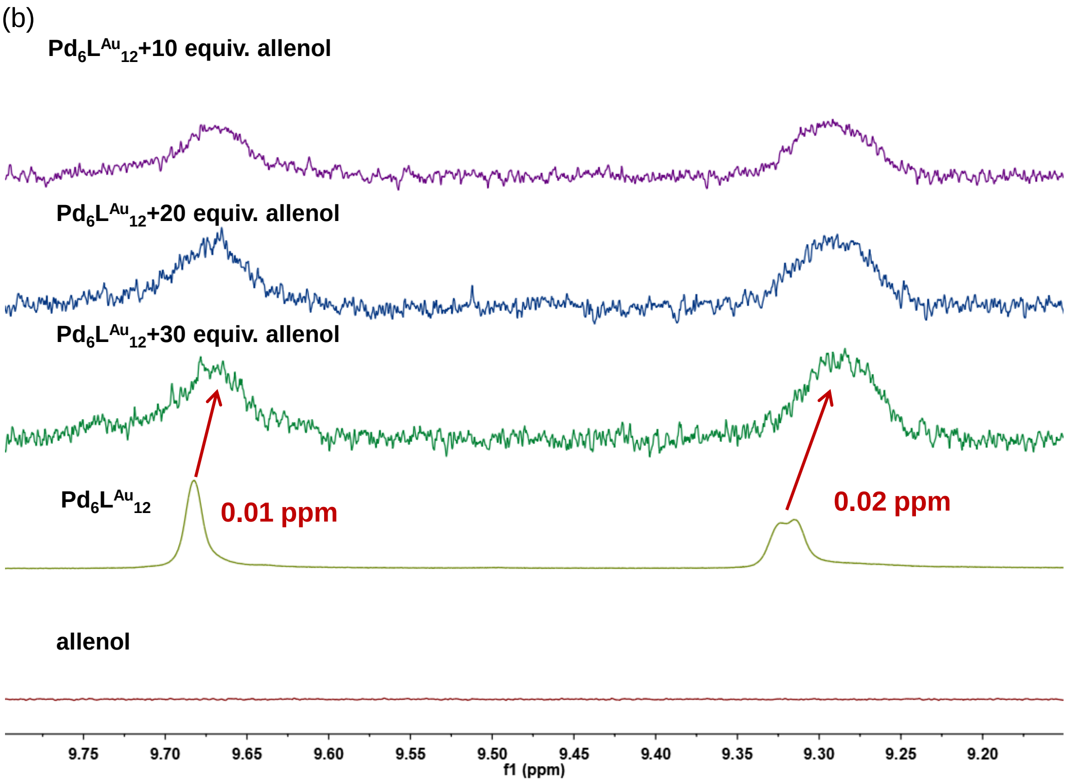

(b)
Pd6LAu12+10 equiv. allenol
Pd6LAu12+20 equiv. allenol
Pd6LAu12+30 equiv. allenol
Pd6LAu12
0.01 ppm
0.02 ppm
allenol
9.75
9.70
9.65
9.60
9.55
9.50
9.45
9.40
9.35
9.30
9.25
9.20
f1 (ppm)

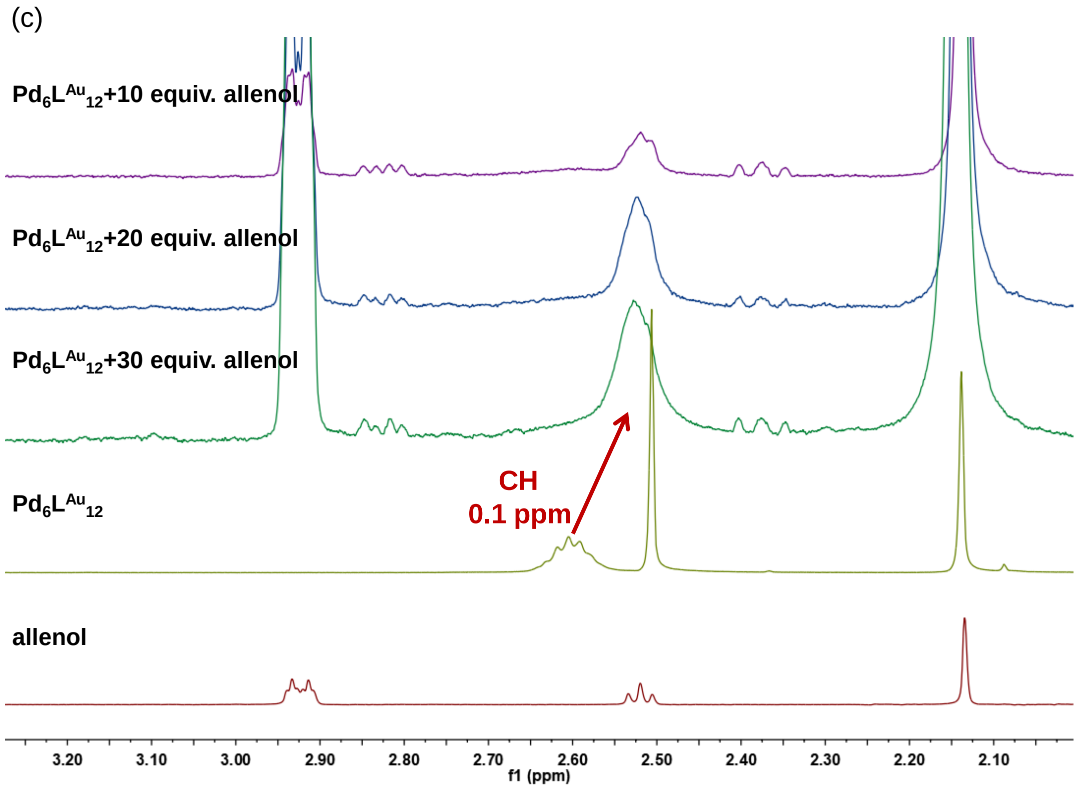

Figure S73. The $Pd_6\mathbf{L}^{\mathbf{Au}}_{12}$-catalyzed cyclization of allenol (different equivalents) at room temperature for 30 min. (a) Overlapped the full ^1H NMR (400 MHz, 298 K) spectra of the $Pd_6\mathbf{L}^{\mathbf{Au}}_{12}$, allenol and the catalyzed reactions in CD_3CN. (b) Zoom in the aromatic area of the spectra. (c) Zoom in the aliphatic area of the spectra. Reaction conditions: The pre-activated $Pd_6L^{Au}_{12}$ was generated by abstracting Cl using an equal equivalent of $AgBF_4$. Each reaction contains the $Pd_6L^{Au}_{12}$ (0.50 μmol) and different equivalents (10, 20, 30 equiv.) of allenol in CD_3CN (600 μL). The reactions were monitored after 30 min based on ^1H NMR by using 1,3,5-trimethoxybenzene as an internal standard.

(a)

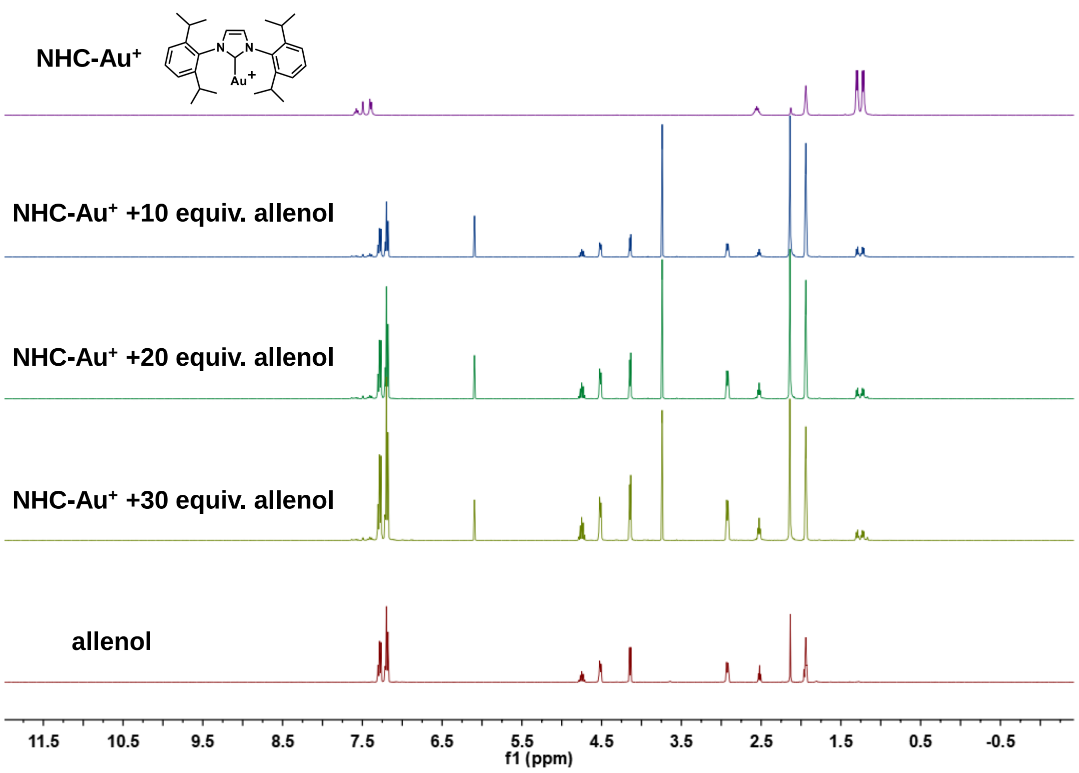
NHC-Au+
NHC-Au+ +10 equiv. allenol
NHC-Au+ +20 equiv. allenol
NHC-Au+ +30 equiv. allenol
allenol
11.5
10.5
9.5
8.5
7.5
6.5
5.5
4.5
3.5
2.5
1.5
0.5
-0.5
f1 (ppm)

(b)

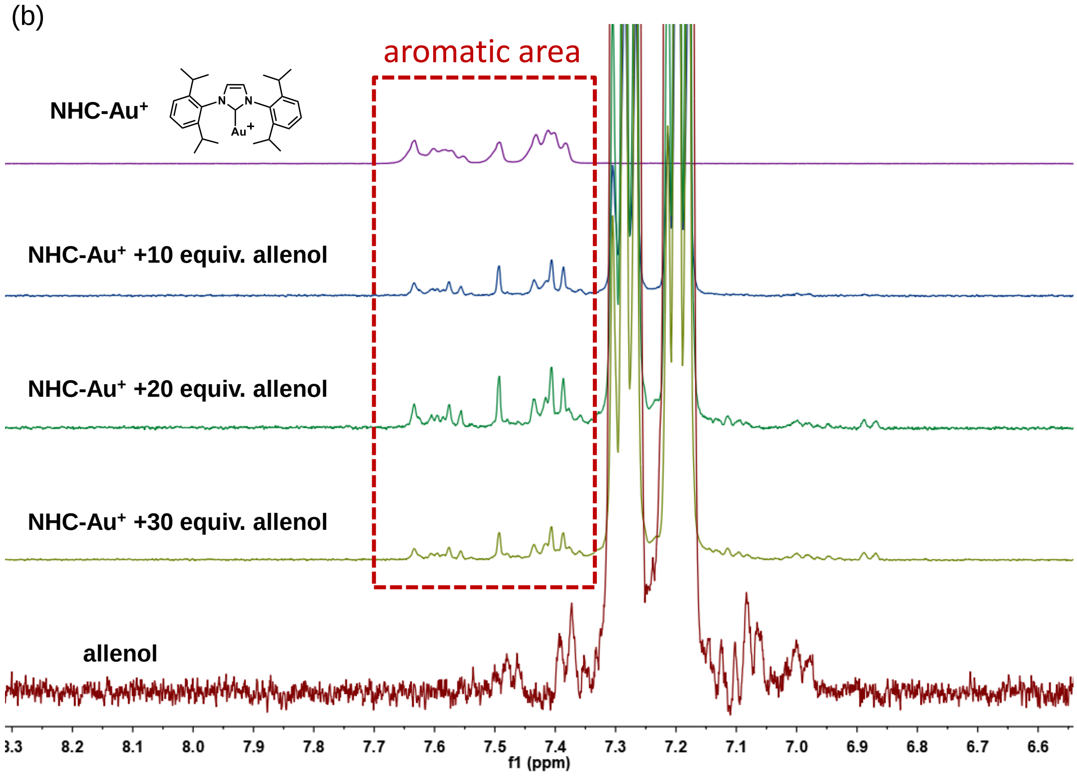
aromatic area
NHC-Au+
NHC-Au+ +10 equiv. allenol
NHC-Au+ +20 equiv. allenol
NHC-Au+ +30 equiv. allenol
allenol
8.2
8.1
8.0
7.9
7.8
7.7
7.6
7.5
7.4
7.3
7.2
7.1
7.0
6.9
6.8
6.7
6.6
f1 (ppm)

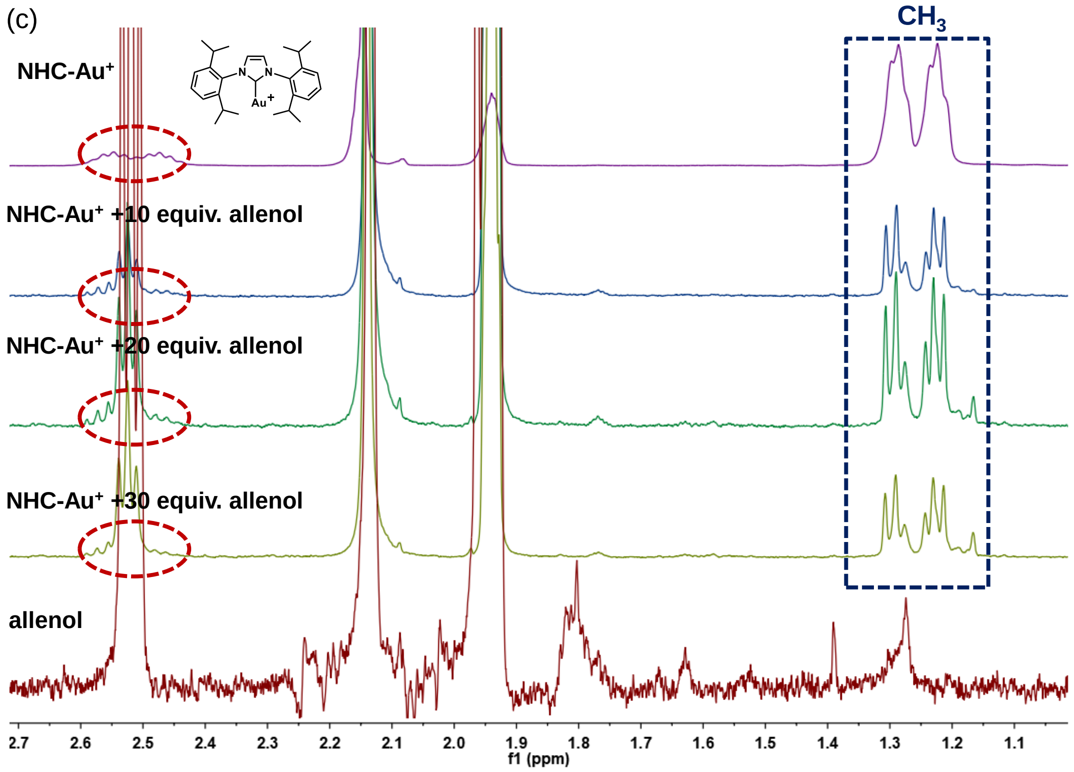

Figure S74. Overlapped the full ^{1}H NMR (400 MHz, 298 K) spectra of NHC-Au$^+$, allenol and the catalyzed reactions of allenol. (a) Overlapped the full ^{1}H NMR (400 MHz, 298 K) spectra of NHC-Au$^+$, allenol and the catalyzed reactions in CD_3CN. (b) Zoom in the aromatic area of the spectra. NHC-Au$^+$ dimer forms in the system due to the water (2.13 ppm) from the CD_3CN. The aromatic area of NHC-Au$^+$ show no chemical shift (in red rectangle). (c) Zoom in the aliphatic area of the spectra. Protons of CH (in red circle) and CH_3 (in blue rectangle) show no chemical shift. Reaction conditions: The pre-activated NHC-Au$^+$ was generated by abstracting Cl using an equal equivalent of $AgBF_4$. Each reaction contains NHC-Au$^+$ (0.50 μmol) and different equivalents (10, 20, 30 equiv.) of allenol in CD_3CN (600 μL). The reactions were monitored after 30 min based on ^{1}H NMR by using 1,3,5-trimethoxybenzene as the internal standard.

S5. Crystallography

X-ray intensities of the complexes were collected on a Bruker D8 VENTURE diffractometer with graphite-monochromated Mo-Kα (λ = 0.71073 Å) using the SMART and SAINT programs.[7,8] The structures were solved by direct methods and refined on $\boldsymbol{F^2}$ by full-matrix least-squares methods with SHELXTL-2018.[9]

For free ligand (**L**$^{\mathbf{free}}$): $C_{37}H_{43}N_4BF_4$, M = 630.56, orthorhombic, space group $P2_12_12_1$, colorless needle, a = 9.4122(4) Å, b = 15.2241(6) Å, c = 24.3198(9) Å， V = 3484.8(2) Å^3, Z = 4, Dc = 1.202 g cm^{-3}, μ(Mo-Kα) = 0.085 mm^{-1}, T = 200(2) K. 6133 Unique reflections, [R_{int} = 0.0890], final R_1 [with I > 2σ(I)] = 0.0639, wR_2 (all data) = 0.1706. CCDC No. 2144003.

In the structural refinement of the free ligand (**L**$^{\mathbf{free}}$), the non-hydrogen atoms were refined anisotropically and the hydrogen atoms were fixed geometrically at calculated distances and allowed to ride on the parent non-hydrogen atoms, the A alert in checkcif is caused by the close packing pattern between adjacent molecules.

Table S4. Crystal data and structure refinement for **L^free^**.

**Identification code**	**L^free^**
Empirical formula	$C_{37}H_{43}BF_4N_4$
Formula weight	630.56
Temperature	200(2) K
Wavelength	0.71073 Å
Crystal system, space group	Orthorhombic, $P2_12_12_1$
Unit cell dimensions	a = 9.4122(4) Å alpha = 90 deg.
	b = 15.2241(6) Å beta = 90 deg.
	c = 24.3198(9) Å gamma = 90 deg.
Volume	3484.8(2) $Å^3$
Z, Calculated density	4, 1.202 Mg/m^3
Absorption coefficient	0.085 mm^{-1}
F(000)	1336
Crystal size	0.1 x 0.09 x 0.08 mm
Theta range for data collection	2.143 to 24.997 deg.
Limiting indices	-11<=h<=11, -18<=k<=18, -28<=l<=28
Reflections collected/unique	113683/6133 [$R_{(int)}$ = 0.0890]
Completeness to theta = 24.997	99.9%
Absorption correction	None
Refinement method	Full-matrix least-squares on F^2
Data/restraints/parameters	6133/0/424
Goodness-of-fit on F^2	1.213
Final R indices [I>2sigma(I)]	R_1 = 0.0639, wR_2 = 0.1631
R indices (all data)	R_1 = 0.0771, wR_2 = 0.1706
Absolute structure parameter	0.1(3)
Extinction coefficient	0.010(2)
Largest diff. peak and hole	0.498 and -0.412 e.$Å^{-3}$
CCDC No.	2144003

Table S5. Bond lengths [Å] and angles [deg] for **L^free^**.

C(2)-C(5)	1.323(6)	C(18)-H(18)	0.9500
C(2)-C(1)	1.328(7)	C(19)-C(20)	1.349(6)
C(2)-H(2A)	0.9500	C(19)-H(19)	0.9500
F(1)-B(1)	1.378(7)	C(20)-H(20)	0.9500
C(1)-N(1)	1.393(7)	C(21)-C(26)	1.390(6)
C(1)-H(1)	0.9500	C(21)-C(22)	1.388(6)
B(1)-F(3)	1.315(7)	C(22)-C(23)	1.389(6)
B(1)-F(2)	1.330(7)	C(22)-C(30)	1.516(6)
B(1)-F(4)	1.347(7)	C(23)-C(24)	1.389(6)
N(2)-C(18)	1.329(5)	C(23)-H(23)	0.9500
N(2)-C(19)	1.377(6)	C(24)-C(25)	1.391(6)
N(2)-C(9)	1.456(5)	C(24)-C(34)	1.483(6)
N(1)-C(3)	1.382(6)	C(25)-C(26)	1.391(6)
N(3)-C(18)	1.336(5)	C(25)-H(25)	0.9500
N(3)-C(20)	1.377(6)	C(26)-C(27)	1.524(6)

N(3)-C(21)	1.452(5)	C(27)-C(29)	1.522(7)
C(3)-C(4)	1.396(7)	C(27)-C(28)	1.527(8)
C(3)-H(3)	0.9500	C(27)-H(27)	1.0000
C(5)-C(4)	1.395(6)	C(28)-H(28A)	0.9800
C(5)-H(5)	0.9500	C(28)-H(28B)	0.9800
C(36)-C(37)	1.310(8)	C(28)-H(28C)	0.9800
C(36)-C(33)	1.338(6)	C(29)-H(29A)	0.9800
C(36)-H(36A)	0.9500	C(29)-H(29B)	0.9800
C(4)-C(6)	1.477(6)	C(29)-H(29C)	0.9800
C(6)-C(11)	1.386(6)	C(30)-C(31)	1.490(9)
C(6)-C(7)	1.399(6)	C(30)-C(32)	1.510(8)
C(7)-C(8)	1.380(6)	C(30)-H(30)	1.0000
C(7)-H(7)	0.9500	C(31)-H(31A)	0.9800
C(9)-C(8)	1.388(6)	C(31)-H(31B)	0.9800
C(9)-C(10)	1.387(6)	C(31)-H(31C)	0.9800
C(8)-C(12)	1.525(6)	C(32)-H(32A)	0.9800
C(10)-C(11)	1.397(6)	C(32)-H(32B)	0.9800
C(10)-C(15)	1.510(6)	C(32)-H(32C)	0.9800
C(11)-H(11)	0.9500	C(33)-C(34)	1.377(7)
C(12)-C(14)	1.515(8)	C(33)-H(33)	0.9500
C(12)-C(13)	1.526(7)	C(34)-C(35)	1.389(7)
C(12)-H(12)	1.0000	C(35)-N(4)	1.384(6)
C(13)-H(13A)	0.9800	C(35)-H(35)	0.9500
C(13)-H(13B)	0.9800	N(4)-C(37)	1.381(8)
C(13)-H(13C)	0.9800	C(37)-H(37)	0.9500
C(14)-H(14A)	0.9800	C(17)-H(17A)	0.9800
C(14)-H(14B)	0.9800	C(17)-H(17B)	0.9800
C(14)-H(14C)	0.9800	C(17)-H(17C)	0.9800
C(15)-C(17)	1.509(9)	C(16)-H(16A)	0.9800
C(15)-C(16)	1.519(9)	C(16)-H(16B)	0.9800
C(15)-H(15)	1.0000	C(16)-H(16C)	0.9800
C(5)-C(2)-C(1)	117.9(4)	C(19)-C(20)-N(3)	107.3(4)
C(5)-C(2)-H(2A)	121.0	C(19)-C(20)-H(20)	126.4
C(1)-C(2)-H(2A)	121.0	N(3)-C(20)-H(20)	126.4
C(2)-C(1)-N(1)	123.4(4)	C(26)-C(21)-C(22)	123.1(3)
C(2)-C(1)-H(1)	118.3	C(26)-C(21)-N(3)	118.9(3)
N(1)-C(1)-H(1)	118.3	C(22)-C(21)-N(3)	118.0(3)
F(3)-B(1)-F(2)	109.5(6)	C(23)-C(22)-C(21)	117.2(4)
F(3)-B(1)-F(4)	105.5(5)	C(23)-C(22)-C(30)	119.7(4)
F(2)-B(1)-F(4)	110.2(6)	C(21)-C(22)-C(30)	123.2(4)
F(3)-B(1)-F(1)	114.6(5)	C(24)-C(23)-C(22)	122.4(4)
F(2)-B(1)-F(1)	107.6(4)	C(24)-C(23)-H(23)	118.8
F(4)-B(1)-F(1)	109.4(5)	C(22)-C(23)-H(23)	118.8
C(18)-N(2)-C(19)	109.4(3)	C(23)-C(24)-C(25)	118.0(4)
C(18)-N(2)-C(9)	124.1(4)	C(23)-C(24)-C(34)	120.5(4)
C(19)-N(2)-C(9)	126.3(3)	C(25)-C(24)-C(34)	121.5(4)
C(3)-N(1)-C(1)	117.5(5)	C(24)-C(25)-C(26)	122.0(4)
C(18)-N(3)-C(20)	108.9(3)	C(24)-C(25)-H(25)	119.0
C(18)-N(3)-C(21)	125.7(3)	C(26)-C(25)-H(25)	119.0

C(20)-N(3)-C(21)	125.3(3)	C(21)-C(26)-C(25)	117.3(4)
N(1)-C(3)-C(4)	120.4(5)	C(21)-C(26)-C(27)	122.2(3)
N(1)-C(3)-H(3)	119.8	C(25)-C(26)-C(27)	120.4(4)
C(4)-C(3)-H(3)	119.8	C(26)-C(27)-C(29)	112.6(4)
C(2)-C(5)-C(4)	124.4(5)	C(26)-C(27)-C(28)	110.6(4)
C(2)-C(5)-H(5)	117.8	C(29)-C(27)-C(28)	110.1(4)
C(4)-C(5)-H(5)	117.8	C(26)-C(27)-H(27)	107.8
C(37)-C(36)-C(33)	118.1(5)	C(29)-C(27)-H(27)	107.8
C(37)-C(36)-H(36A)	120.9	C(28)-C(27)-H(27)	107.8
C(33)-C(36)-H(36A)	120.9	C(27)-C(28)-H(28A)	109.5
C(5)-C(4)-C(3)	116.3(4)	C(27)-C(28)-H(28B)	109.5
C(5)-C(4)-C(6)	121.7(4)	H(28A)-C(28)-H(28B)	109.5
C(3)-C(4)-C(6)	122.0(4)	C(27)-C(28)-H(28C)	109.5
C(11)-C(6)-C(7)	118.4(4)	H(28A)-C(28)-H(28C)	109.5
C(11)-C(6)-C(4)	121.0(4)	H(28B)-C(28)-H(28C)	109.5
C(7)-C(6)-C(4)	120.6(4)	C(27)-C(29)-H(29A)	109.5
C(8)-C(7)-C(6)	121.9(4)	C(27)-C(29)-H(29B)	109.5
C(8)-C(7)-H(7)	119.0	H(29A)-C(29)-H(29B)	109.5
C(6)-C(7)-H(7)	119.0	C(27)-C(29)-H(29C)	109.5
C(8)-C(9)-C(10)	123.9(4)	H(29A)-C(29)-H(29C)	109.5
C(8)-C(9)-N(2)	117.6(4)	H(29B)-C(29)-H(29C)	109.5
C(10)-C(9)-N(2)	118.5(4)	C(31)-C(30)-C(22)	110.4(5)
C(7)-C(8)-C(9)	117.1(4)	C(31)-C(30)-C(32)	110.4(5)
C(7)-C(8)-C(12)	120.3(4)	C(22)-C(30)-C(32)	112.8(4)
C(9)-C(8)-C(12)	122.6(4)	C(31)-C(30)-H(30)	107.7
C(9)-C(10)-C(11)	116.5(4)	C(22)-C(30)-H(30)	107.7
C(9)-C(10)-C(15)	122.8(4)	C(32)-C(30)-H(30)	107.7
C(11)-C(10)-C(15)	120.6(4)	C(30)-C(31)-H(31A)	109.5
C(6)-C(11)-C(10)	122.0(4)	C(30)-C(31)-H(31B)	109.5
C(6)-C(11)-H(11)	119.0	H(31A)-C(31)-H(31B)	109.5
C(10)-C(11)-H(11)	119.0	C(30)-C(31)-H(31C)	109.5
C(14)-C(12)-C(8)	110.6(4)	H(31A)-C(31)-H(31C)	109.5
C(14)-C(12)-C(13)	111.0(4)	H(31B)-C(31)-H(31C)	109.5
C(8)-C(12)-C(13)	111.5(4)	C(30)-C(32)-H(32A)	109.5
C(14)-C(12)-H(12)	107.9	C(30)-C(32)-H(32B)	109.5
C(8)-C(12)-H(12)	107.9	H(32A)-C(32)-H(32B)	109.5
C(13)-C(12)-H(12)	107.9	C(30)-C(32)-H(32C)	109.5
C(12)-C(13)-H(13A)	109.5	H(32A)-C(32)-H(32C)	109.5
C(12)-C(13)-H(13B)	109.5	H(32B)-C(32)-	109.5

		H(32C)	
H(13A)-C(13)-H(13B)	109.5	C(36)-C(33)-C(34)	123.9(5)
C(12)-C(13)-H(13C)	109.5	C(36)-C(33)-H(33)	118.0
H(13A)-C(13)-H(13C)	109.5	C(34)-C(33)-H(33)	118.0
H(13B)-C(13)-H(13C)	109.5	C(35)-C(34)-C(33)	116.4(4)
C(12)-C(14)-H(14A)	109.5	C(35)-C(34)-C(24)	122.1(4)
C(12)-C(14)-H(14B)	109.5	C(33)-C(34)-C(24)	121.5(4)
H(14A)-C(14)-H(14B)	109.5	C(34)-C(35)-N(4)	120.5(5)
C(12)-C(14)-H(14C)	109.5	C(34)-C(35)-H(35)	119.7
H(14A)-C(14)-H(14C)	109.5	N(4)-C(35)-H(35)	119.7
H(14B)-C(14)-H(14C)	109.5	C(37)-N(4)-C(35)	117.3(5)
C(17)-C(15)-C(10)	110.4(5)	C(36)-C(37)-N(4)	123.5(5)
C(17)-C(15)-C(16)	110.1(5)	C(36)-C(37)-H(37)	118.2
C(10)-C(15)-C(16)	113.1(5)	N(4)-C(37)-H(37)	118.2
C(17)-C(15)-H(15)	107.7	C(15)-C(17)-H(17A)	109.5
C(10)-C(15)-H(15)	107.7	C(15)-C(17)-H(17B)	109.5
C(16)-C(15)-H(15)	107.7	H(17A)-C(17)-H(17B)	109.5
N(2)-C(18)-N(3)	107.8(3)	C(15)-C(17)-H(17C)	109.5
N(2)-C(18)-H(18)	126.1	H(17A)-C(17)-H(17C)	109.5
N(3)-C(18)-H(18)	126.1	H(17B)-C(17)-H(17C)	109.5
C(20)-C(19)-N(2)	106.7(4)	C(15)-C(16)-H(16A)	109.5
C(20)-C(19)-H(19)	126.7	C(15)-C(16)-H(16B)	109.5
N(2)-C(19)-H(19)	126.7	H(16A)-C(16)-H(16B)	109.5
C(19)-C(20)-N(3)	107.3(4)		

For Au ligand (**L**^{AuCl}): $C_{37}H_{42}N_4AuCl$, M = 775.16, orthorhombic, space group $\underline{P}2_12_12_1$, colorless block, a = 9.2790(4) Å, b = 15.5601(6) Å, c = 24.1767(10) Å, V = 3490.7(2) Å^3, Z = 4, *Dc* = 1.475 g cm^{-3}, μ(Mo-Kα) = 4.321 mm^{-1}, T = 200(2) K. 6013 Unique reflections, [R_{int} = 0.0208], final R_1 [with I > 2σ(I)] = 0.0584, wR_2 (all data) = 0.1795. CCDC No. 2144004.

In the structural refinement of the Au ligand (**L**^{AuCl}), except the Cl atom with quite low thermal parameter coordinated at the Au atom, other non-hydrogen atoms were refined anisotropically. The hydrogen atoms were fixed geometrically at calculated distances and allowed to ride on the parent non-hydrogen atoms.

Table S6. Crystal data and structure refinement for **L**^{AuCl}.

**Identification code**	**L**^{AuCl}
Empirical formula	$C_{37}H_{42}AuClN_4$
Formula weight	775.16
Temperature	200(2) K
Wavelength	0.71073 Å
Crystal system, space group	Orthorhombic, $\underline{P}2_12_12_1$
Unit cell dimensions	a = 9.2790(4) Å alpha = 90 deg.
	b = 15.5601(6) Å beta = 90 deg.
	c = 24.1767(10) Å gamma = 90 deg.
Volume	3490.7(2) Å^3
Z, Calculated density	4, 1.475 Mg/m^3
Absorption coefficient	4.321 mm^{-1}
F(000)	1552
Theta range for data collection	2.351 to 24.999 deg.
Limiting indices	-11<=h<=8, -18<=k<=18, -28<=l<=28
Reflections collected/unique	21530/6013 [$R_{(int)}$ = 0.0208]
Completeness to theta = 24.999	99.2%
Refinement method	Full-matrix least-squares on F^2
Data/restraints/parameters	6013/12/384
Goodness-of-fit on F^2	1.056
Final R indices [I>2sigma(I)]	R_1 = 0.0584, wR_2 = 0.1784
R indices (all data)	R_1 = 0.0593, wR_2 = 0.1795
Absolute structure parameter	0.18(2)
Extinction coefficient	n/a
Largest diff. peak and hole	7.438 and -1.393 e.Å^{-3}
CCDC No.	2144004

Table S7. Selected bond lengths [Å] and angles [deg] for $\mathbf{L}^{\mathbf{AuCl}}$.

Au(1)-C(20)	1.974(17)
Au(1)-Cl(1)	2.376(2)
C(20)-Au(1)-Cl(1)	177.8(4)
N(3)-C(20)-Au(1)	128.8(12)
N(2)-C(20)-Au(1)	127.2(11)

For Au cage ($Pd_6\mathbf{L}^{\mathbf{AuCl}}_{\mathbf{12}}$): $C_{517}H_{643}Au_{12}B_{12}F_{48}Cl_{12}N_{63}O_{28}Pd_6$, M = 12657.02, trigonal, space group $R\bar{3}$, colorless block, a = 30.373(3) Å, c = 77.172(13) Å, V = 61655(15) Å^3, Z = 3, *Dc* = 1.023 g cm^{-3}, μ(Mo-Kα) = 2.352 mm^{-1}, T = 200(2) K. 24104 Unique reflections, [R_{int} = 0.0990], final R_1 [with I > 2σ(I)] = 0.0991, wR_2 (all data) = 0.3620. CCDC No. 2144005.

In the structural refinement of the Au cage ($Pd_6\mathbf{L}^{\mathbf{AuCl}}_{\mathbf{12}}$), except some disordered atoms and partly occupied solvent molecules, other non-hydrogen atoms were refined anisotropically. Hydrogen atoms on the ligand backbone were fixed geometrically at calculated distances and allowed to ride on the parent non-hydrogen atoms. One of the Au atoms and the coordinated Cl atom on it, some carbon atoms on the isopropyl moieties, several F atoms in the BF_4^- counter ions, were disordered into two parts with the ***s.o.f.*** *(site occupied factors)* being fixed as 0.5, respectively. To assist the stability of refinements, several restrains were applied: the C-C bond distances in some isopropyl moieties, and respective bond distances in the partly occupied solvent molecules were fixed at suitable values. Thermal parameters on adjacent atoms in some of isopropyl moieties, one of the BF_4^- counter ion, and solvent molecules were restrained to be similar, respectively. The A alert in checkcif was caused by the presence of lots of highly disordered solvent molecule which could not be located.

Table S8. Crystal data and structure refinement for $Pd_6\mathbf{L}^{AuCl}_{12}$.

**Identification code**	$Pd_6\mathbf{L}^{AuCl}_{12}$
Empirical formula	$C_{517}H_{643}Au_{12}B_{12}Cl_{12}F_{48}N_{63}O_{28}Pd_6$
Formula weight	12657.02
Temperature	200(2) K
Wavelength	0.71073 Å
Crystal system, space group	Trigonal, $R\bar{3}$
Unit cell dimensions	a = 30.373(3) Å alpha = 90 deg.<br>b = 30.373(3) Å beta = 90 deg.<br>c = 77.172(13) Å gamma = 120 deg.
Volume	61655(15) $Å^3$
Z, Calculated density	3, 1.023 Mg/m^3
Absorption coefficient	2.352 mm^{-1}
F(000)	18990
Crystal size	0.230 x 0.200 x 0.200 mm
Theta range for data collection	1.341 to 24.999 deg.
Limiting indices	-36<=h<=36, -36<=k<=36, -91<=l<=91
Reflections collected/unique	545606/24104 [$R_{(int)}$ = 0.0990]
Completeness to theta = 24.999	99.9%
Refinement method	Full-matrix least-squares on F^2
Data/restraints/parameters	24104/617/1038
Goodness-of-fit on F^2	1.047
Final R indices [I>2sigma(I)]	R_1 = 0.0991, wR_2 = 0.3268
R indices (all data)	R_1 = 0.1278, wR_2 = 0.3620
Extinction coefficient	n/a
Largest diff. peak and hole	4.784 and -1.242 e. $Å^{-3}$
CCDC No.	2144005

Table S9. Selected bond lengths [Å] and angles [deg] for $Pd_6\mathbf{L}^{\mathbf{AuCl}}_{12}$.

Au(1)-C(120)	1.945(15)	Au(2')-C(220)	2.162(15)
Au(1)-Cl(2)	2.378(2)	Au(2')-Cl(1)	2.337(11)
Au(2)-C(220)	1.815(16)	Pd(1)-N(8)#1	2.006(6)
Au(2)-Cl(1')	2.433(10)	Pd(1)-N(1)	2.013(6)
Au(2)-Cl(1)	2.713(11)	Pd(1)-N(5)	2.020(6)
Au(2')-Cl(1')	2.120(10)	Pd(1)-N(4)#2	2.022(6)
C(120)-Au(1)-Cl(2)	177.8(3)	Au(2')-Cl(1')-Au(2)	7.5(3)
C(220)-Au(2)-Cl(1')	178.9(5)	C(105)-N(1)-Pd(1)	122.3(5)
C(220)-Au(2)-Cl(1)	165.9(5)	C(101)-N(1)-Pd(1)	118.8(6)
Cl(1')-Au(2)-Cl(1)	13.4(4)	C(137)-N(4)-Pd(1)#6	120.9(5)
Cl(1')-Au(2')-C(220)	165.5(6)	C(136)-N(4)-Pd(1)#6	118.8(6)
Cl(1')-Au(2')-Cl(1)	16.2(4)	C(205)-N(5)-C(201)	119.5(7)
C(220)-Au(2')-Cl(1)	176.0(5)	C(205)-N(5)-Pd(1)	121.1(5)
N(8)#1-Pd(1)-N(1)	177.8(2)	C(201)-N(5)-Pd(1)	119.4(5)
N(8)#1-Pd(1)-N(5)	89.2(3)	C(236)-N(8)-Pd(1)#7	120.7(5)
N(1)-Pd(1)-N(5)	91.0(3)	C(237)-N(8)-Pd(1)#7	121.0(5)
N(8)#1-Pd(1)-N(4)#2	89.2(3)	N(2)-C(120)-Au(1)	128.2(8)
N(1)-Pd(1)-N(4)#2	90.7(3)	N(3)-C(120)-Au(1)	127.7(9)
N(5)-Pd(1)-N(4)#2	178.4(3)	N(6)-C(220)-Au(2)	129.7(9)
Cl(1')-Cl(1)-Au(2')	63.0(15)	N(7)-C(220)-Au(2)	126.5(9)
Cl(1')-Cl(1)-Au(2)	58.4(15)	N(6)-C(220)-Au(2')	131.2(8)
Au(2')-Cl(1)-Au(2)	4.8(3)	N(7)-C(220)-Au(2')	125.6(9)
Cl(1)-Cl(1')-Au(2')	100.8(18)	Au(2)-C(220)-Au(2')	7.4(3)
Cl(1)-Cl(1')-Au(2)	108.2(18)		

S6. References

1. G. Kumar, R. Guda, A. Husain, R. Bodapati, S. K. Das, *Inorg. Chem.*, 2017, **56**, 5017–5025.
2. J. L. Arbour, H. S. Rzepa, A. J. P. White, K. K. M. Hii, *Chem. Commun.*, 2009, 7125–7127.
3. A. Fürstner, S. F. Dipl.-Chem., O. Larionov, Y. Takahashi, T. Kubota, J. Kobayashi, *Chem.-Eur. J.*, 2009, **15**, 4011–4029.
4. R. Gramage-Doria, J. Hessels, S. H. A. M. Leenders, O. Tröppner, M. Dürr, I. Ivanović-Burmazović, J. N. H. Reek, *Angew. Chem., Int. Ed.*, 2014, 53, 13380–13384.
5. D. M. T. Chan, T. B. Marder, D. Milstein, N. J. Taylor, *J. Am. Chem. Soc.*, 1987, **109**, 6385–6388.
6. D. A. Poole III, E. O. Bobylev, S. Mathew, J. N. H. Reek, *Chem. Sci.*, 2020, **11**, 12350–12357.
7. SMART. *Data collection software* (*Version 5.629*) (Bruker AXS Inc., Madison, WI, 2003).
8. SAINT. *Data reduction software* (*Version 6.45*) (Bruker AXS Inc., Madison, WI, 2003).
9. G. M. Sheldrick, Crystal structure refinement with SHELXL. *Acta Cryst.*, 2015, **C71**, 3–8.
